# Talaropeptides A-D: Structure and Biosynthesis of Extensively *N*-methylated Linear Peptides From an Australian Marine Tunicate-Derived *Talaromyces* sp.

**DOI:** 10.3389/fchem.2018.00394

**Published:** 2018-09-04

**Authors:** Pradeep Dewapriya, Zeinab G. Khalil, Pritesh Prasad, Angela A. Salim, Pablo Cruz-Morales, Esteban Marcellin, Robert J. Capon

**Affiliations:** ^1^Institute for Molecular Bioscience, The University of Queensland, St Lucia, QLD, Australia; ^2^Australian Institute for Bioengineering and Nanotechnology, The University of Queensland, St Lucia, QLD, Australia; ^3^Joint BioEnergy Institute, Emeryville, CA, United States

**Keywords:** marine-derived, fungus, *Talaromyces*, talaropeptide, NRPS, linear peptide, secondary metabolite, natural product

## Abstract

An Australian marine tunicate-derived fungus, *Talaromyces* sp. CMB-TU011 was subjected to a program of analytical microbioreactor (MATRIX) cultivations, supported by UHPLC-QTOF profiling, to reveal conditions for producing a new class of extensively *N*-methylated 11-12 residue linear peptides, talaropeptides A-D (**2**-**5**). The structures for **2**-**5**, inclusive of absolute configurations, were determined by a combination of detailed spectroscopic and chemical (e.g., C_3_ and C_18_ Marfey's) analyses. We report on the biological properties of **2**-**5**, including plasma stability, as well as antibacterial, antifungal and cell cytotoxicity. The talaropeptide mega non-ribosomal peptide synthetase (NRPS) is described, as second only in size to that for the fungus-derived immunosuppressant cyclosporine (an 11-residue extensively *N*-methylated cyclic peptide).

## Introduction

**Figure 1:**
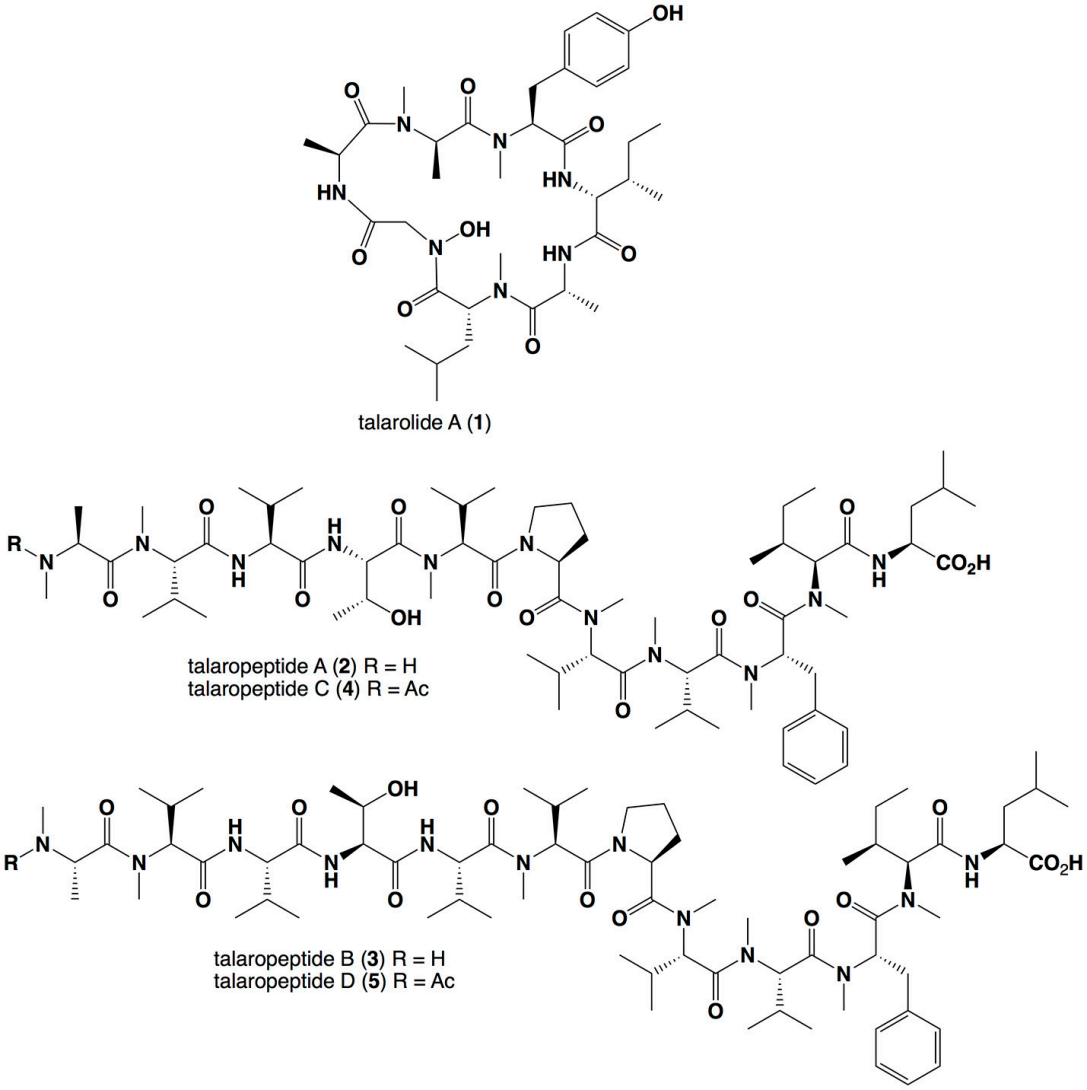
Structures for the *Talaromyces* sp. CMB-TU011 peptides **1**–**5**.

In an earlier study, we described the structure elucidation of a first-in-class cyclic hexapeptide containing a rare hydroxamate residue, talarolide A (**1**) (Figure 1), isolated from an Australian marine tunicate-derived fungus, *Talaromyces* sp. CMB-TU011 (Dewapriya et al., 2017). In an effort to optimize the production of **1**, we now report on a 24-well microbioreactor cultivation analysis (known in-lab as the MATRIX), using a combination of 11 different media and 3 phases (i.e., solid agar, as well as static and shaken broth). *In situ* solvent extraction on individual MATRIX culture wells yielded 33 extracts, which were subjected to UHPLC-DAD and UHPLC-QTOF-MS/MS analysis. While this study successfully revealed optimal conditions for the production of **1**, including new analogs (work-in-progress), it also revealed conditions where talarolide production was fully suppressed in favor of a new class of extensively *N*-methylated linear peptides. This report provides an account of the production, isolation and characterisation of these new peptides, talaropeptides A-D (**2**-**5**), where structure elucidation inclusive of absolute configurations was achieved by a combination of detailed spectroscopic and chemical analyses. We also take the opportunity to report on the biological properties of **2**-**5**, and document the mega non-ribosomal peptide synthetase (NRPS) responsible for the biosynthesis of talaropeptides.

## Materials and methods

### General experimental details

Specific rotations ([α]_D_) were measured on a JASCO P-1010 polarimeter in a 100 × 2 mm cell at room temperature. NMR spectra were acquired on a Bruker Avance 600 MHz spectrometer with either a 5 mm PASEL ^1^H/D–^13^C Z-Gradient probe or 5 mm CPTCI ^1^H/^19^F-^13^C/^15^N/DZ-Gradient cryoprobe, controlled by TopSpin 2.1 software. In all cases, NMR spectra were acquired at 25°C (unless otherwise specified) in hexadeuterated dimethylsulfoxide (DMSO-*d*_6_) and tetradeuterated methanol (methanol-*d*_4_) with referencing to residual ^1^H (δ_H_ 2.50 and δ_H_ 3.31, respectively) or ^13^C (δ_C_ 39.51 and δ_C_ 49.15, respectively) NMR resonances. HPLC-DAD-ESIMS data were acquired on an Agilent 1100 series separation module equipped with an Agilent 1100 series HPLC/MSD mass detector and Agilent diode array detector. Semi-preparative and preparative HPLCs were performed using Agilent 1100 series HPLC instruments with corresponding detectors, fraction collectors, and software. HRESI(+)MS spectra were obtained on a Bruker micrOTOF mass spectrometer by direct injection in MeCN at 3 μL/min using sodium formate clusters as an internal calibrant. UHPLC-QTOF analysis was performed on UHPLC-QTOF instrument comprising an Agilent 1290 Infinity II UHPLC equipped with a Zorbax C_8_ column (2.1 mm × 50 mm, 1.8 μm particles), running with H_2_O/MeCN inclusive of 0.1% formic acid coupled to an Agilent 6545 Q-TOF. MS/MS analysis was performed on the same instrument for ions detected in the full scan at an intensity above 1,000 counts at 10 scans/s, with an isolation width of 4 ~*m*/*z* using a fixed collision energy and a maximum of 3 selected precursors per cycle. *N*α-(2,4-dinitro-5-fluorophenyl)-l-alaninamide (l-FDAA, synonym 1-fluoro-2-4-dinitrophenyl-5-l-alanine amide) and *N*α-(2,4-dinitro-5-fluorophenyl)-d-alanine amide (d-FDAA, synonym 1-fluoro-2-4-dinitrophenyl-5-d-alanine amide) were purchased from NovaBiochem. Amino acids and standards were purchased from NovaBiochem, BAChem Biosciences, Sigma, Fluka, or Merck.

### Fungus isolation and taxonomy

*Talaromyces* sp. CMB-TU011 was isolated from an unidentified tunicate collected near Tweed Heads, NSW, Australia, and taxonomically identified as previously reported (Dewapriya et al., 2017).

### Analytical (MATRIX) cultivation of *Talaromyces* sp. CMB-TU011

*Talaromyces* sp. CMB-TU011 was cultured in a 24-well microbioreactor plate (Khalil et al., 2014) using a combination of 11 culture media and 3 phases (i.e., solid agar, and liquid static and liquid shaken) known in-lab as the MATRIX (Table S1). Briefly, a sterile loop was used to transfer mycelia/spores from an agar plate cultivation to 24-well microbioreactor plate (2.5 mL agar for solid cultures, 1.5 mL of broth for liquid cultures). The microbioreactor plates were sealed with air permeable membranes and incubated at 26.5°C for 10 days (190 rpm for shaken broth). The resulting 33 cultures were extracted *in situ* with EtOAc (2.0 mL/well), with the decanted solvent filtered and dried under N_2_. Secondary metabolite production was then analyzed by HPLC-DAD-ESIMS and UHPLC-QTOF.

### Scale-up cultivation of *Talaromyces* sp. CMB-TU011 and isolation of 2-5

Agar cubes (~1 cm^2^) recovered from 7 day CMB-TU011 agar plate cultures (3.3% artificial sea salt containing M1 medium) were used to inoculate 10 flasks (500 mL) charged with YES broth (160 mL). Individual flasks were covered with air permeable sterile cotton plugs, and incubated under static conditions for 10 days at 26.5°C, after which the combined broths/mycelia were extracted with EtOAc (4 × 500 mL) to yield a crude extract (1.65 g) which was partitioned between hexane (200 mL) and 1% aqueous MeOH (50 mL) and dried *in vacuo* to yield hexane (495 mg) and MeOH (1.15 g) solubles. The MeOH solubles were further fractionated by gel chromatography (Sephadex® LH-20, MeOH) into 20 fractions, which were selectively combined on the basis of HPLC-DAD-ESIMS analysis (Zorbax SB-C_8_ column, analytical gradient 90% H_2_O/MeCN – 100% MeCN inclusive of an isocratic 0.05% formic acid) to yield a fraction of interest (49 mg) that was resolved by optimized semi-preparative HPLC (Zorbax SB C_3_ column (9.4 mm × 25 cm), 40% MeCN/H_2_O elution at 3.0 mL/min inclusive of an isocratic 0.01% TFA modifier) to yield talaropeptide A (**2**) (*t*_R_ = 7.11 min, 1.3 mg), talaropeptide B (**3**) (*t*_R_ = 8.78 min, 1.3 mg), talaropeptide C (**4**) (*t*_R_ = 16.89 min, 1.8 mg), and talaropeptide D (**5**) (*t*_R_ = 22.96 min, 2.8 mg) (Supplementary Scheme S1).

*Talaropeptide A (2):* white powder; [α]${{}_{\text{D}}}^{22}$ −135.8 (c 0.04, MeOH); 1D and 2D NMR (600 MHz, DMSO-*d*_6_) see Table 1 and Supplementary Material; HRESI(+)MS *m*/*z* 1254.8435 [(M+H)^+^] (calcd for C_65_H_112_N_11_O_13_, 1254.8436).

**Table 1:** 1D NMR (DMSO-*d*_6_) data for talaropeptide A (**2**).

**#**	**δ_H_ m (*J* in Hz)**	**δ_C_**
***N*****-Me-L*****-*****Ala**^1^		
1		171.4
2	5.36, m	48.8
3	1.09^a^	14.3
*N*-Me	2.96, s	30.3^f^
***N*****-Me-L*****-*****Val**^2^		
1		167.5
2	4.82, m	59.0
3	2.10^b^	27.1
4	0.87^c^	18.9^g^
5	0.71, d (*6.4*)	18.1^h^
*N*-Me	2.87, s	29.5^i^
**L*****-*****Val**^3^		
1		171.0
2	4.42, m	56.2
3	1.97, m	29.4^i^
4, 5	0.80^d^	18.1^h^
*N*-H	8.30, d (*8.6*)	
**L*****-*****Thr**^4^		
1		^nd^
2	4.15, m	55.1
3	3.80, m	66.6
4	1.10^a^	19.3^g^
4-OH	5.84, d (*4.6*)	
*N*-H	^nd^	
***N*****-Me-L*****-*****Val**^5^		
1		^nd^
2	4.68^e^	61.3
3	2.10^b^	26.3^j^
4	0.87^c^	18.9^g^
5	0.82^d^	19.2^g^
*N*-Me	3.04, s	30.4^f^
**L*****-*****Pro**^6^		
1		172.1
2	4.69^e^	56.3
3	a 2.09^b^	28.5^k^
	b 1.50, m	
4	a 1.92, m	24.4^l^
	b 1.79, m	
5	a 3.70, m	47.0
	b 3.53, m	
***N*****-Me-L*****-*****Val**^7^		
1		169.3
2	4.80, m	57.5
3	2.15, m	26.2^j^
4, 5	0.76, m	18.0^h^
*N*-Me	2.87, s	29.7^i^
***N*****-Me-L*****-*****Val**^8^		
1		168.5
2	4.91, d (*10.5*)	57.7
3	2.10^b^	26.3^j^
4	0.73, d (*6.5*)	19.1^g^
5	0.56, d (*6.5*)	17.1
*N*-Me	2.18, s	28.6^k^
***N*****-Me-L*****-*****Phe**^9^		
1		170.0
2	5.77, t (*7.6*)	53.1
3	2.94, m	34.3
4		137.3
5/9	7.71, m	129.1
6/8	7.22, m	128.0
7	7.17, m	126.3
*N*-Me	2.85, s	29.9^i^
***N*****-Me-L*****-*****Ile**^10^		
1		169.7
2	4.69^e^	59.4
3	1.94, m	32.3
4	a 1.20, m	24.3^l^
	b 0.90, m	
5	0.78^d^	10.0
6	0.86^c^	15.1
*N*-Me	2.94, s	30.4^f^
**L*****-*****Leu**^11^		
1		173.7
2	4.11, m	50.3
3	a 1.56, m	39.2
	b 1.48, m	
4	1.57, m	24.2^l^
5	0.88^c^	22.8
6	0.80^d^	21.3
*N*-H	8.17, d (*7.4*)	
OH	^nd^	

a−e *Assignments with the same superscript are overlapping*.

f−l *Assignments with the same superscript may be interchanged*.

nd *resonances not detected*.

*Talaropeptide B (3):* white powder; [α]${{}_{\text{D}}}^{22}$-110.0 (c 0.05, MeOH); 1D and 2D NMR (600 MHz, methanol-*d*_4_) see Table 2 and Supplementary Material; HRESI(+)MS *m*/*z* 1375.8920 [M+Na]^+^ (calcd for C_70_H_120_N_12_O_14_Na, 1375.8939).

**Table 2:** 1D NMR (methanol-*d*_4_) data for talaropeptide B (**3**).

**#**	**δ_H_ m (*J* in Hz)**	**δ_C_**
***N*****-Me-L*****-*****Ala**^1^		
1		174.3
2	5.43, m	51.4
3	1.27, d (*6.9*)	14.8
*N*-Me	3.14, s	31.7^j^
***N*****-Me-L*****-*****Val**^2^		
1		170.1
2	4.98, d (*10.8*)	61.6
3	2.24^a^	28.6
4	0.97^b^	19.8^k^
5	0.82, d (*6.6*)	19.4^l^
*N*-Me	3.01, s	31.1^m^
**L*****-*****Val**^3^		
1		173.6^n^
2	4.63, d (*7.6*)	56.2
3	2.05^c^	31.7^j^
4	0.92^d^	18.6°
5	0.93^e^	18.9°
**L*****-*****Thr**^4^		
1		173.6^n^
2	4.82^f^	56.6
3	4.01, m	68.9
4	1.19, d (*6.3*)	20.3^k^
**L*****-***Val*		
1		^nd^
2	3.50, br s	60.3
3	2.08, m	32.4
4	0.93^e^	19.3^l^
5	0.96^b^	17.9
***N*****-Me-L*****-*****Val**^5^		
1		171.9
2	4.69, d (*11.0*)	63.6
3	2.23^a^	28.0
4	0.89^g^	19.9^k^
5	0.80, d (*6.5*)	19.2^l^
*N*-Me	3.22, s	31.9^j^
**L*****-*****Pro**^6^		
1		174.7
2	4.80^f^	58.6
3	a 2.21^h^	30.2^p^
	b 1.66, m	
4	a 2.04^c^	25.8^q^
4	b 1.91, m	
5	3.71, m	49.0
***N*****-Me-L*****-*****Val**^7^		
1		171.6
2	4.91, d (*10.6*)	60.2
3	2.27, m	28.2
4	0.89^g^	20.5
5	0.86^i^	18.6°
*N*-Me	3.02, s	31.1^m^
***N*****-Me-L*****-*****Val**^8^		
1		171.0^r^
2	5.03, d (*10.5*)	60.1
3	2.22^h^	28.4
4	0.83, d (*6.7*)	20.2^k^
5	0.67, d (*6.7*)	18.2
*N*-Me	2.26, s	30.3^p^
***N*****-Me-L*****-*****Phe**^9^		
1		172.8
2	5.94, dd (*10.3, 5.4)*	55.4
3	3.04, m	35.9
4		138.3
5/9	7.21, m	130.5
6/8	7.28, m	129.7
7	7.24, m	128.2
*N*-Me	2.98, s	31.5^j^
***N*****-Me-L*****-*****Ile**^10^		
1		171.1^r^
2	4.76, d (*11.4*)	62.6
3	2.09, m	33.4
4	a 1.31, m	25.9^q^
	b 1.02, m	
5	0.87^i^	10.7
6	0.94, d (*6.6*)	16.0
*N*-Me	3.04, s	31.6^j^
**L*****-*****Leu**^11^		
1		^nd^
2	4.34, br s	54.5
3	a 1.64, m	43.1
	b 1.55, m	
4	1.59, m	26.5
5	0.92^d^	23.8
6	0.91^d^	22.6

a−i *Assignments with the same superscript are overlapping*.

j−r *Assignments with the same superscript may be interchanged*.

nd *resonances not detected*.

*Talaropeptide C (4):* white powder; [α]${{}_{\text{D}}}^{22}$ −151.6 (c 0.05), MeOH); 1D and 2D NMR (600 MHz, methanol-*d*_4_) see Table 3 and Supplementary Material; HRESI(+)MS *m*/*z* 1318.8376 [(M+Na)^+^] (calcd for C_67_H_113_N_11_O_14_Na, 1318.8361).

**Table 3:** 1D NMR (methanol-*d*_4_) data for talaropeptide C (**4**).

**#**	**δ_H_ m (*J* in Hz)**	**δ_C_**
***N*****-Me-L*****-*****Ala**^1^		
1		174.3^h^
2	5.44, m	51.4
3	1.27, d (*6.9*)	14.8
*N*-Me	3.14, s	31.7^i^
*N*-COCH_3_		173.6^j^
*N*-COCH_3_	1.99, s	22.3^k^
***N*****-Me-L*****-*****Val**^2^		
1		170.1
2	4.98, d (*10.9*)	61.5
3	2.24^a^	28.6
4	0.97, d (*6.6*)	19.9^l^
5	0.82, d (*6.6*)	19.3
*N*-Me	3.01, s	31.1^m^
**L*****-*****Val**^3^		
1		173.7^j^
2	4.63, d (*7.6*)	56.2
3	2.05^b^	31.8^i^
4	0.91^c^	19.9^l^
5	0.93^c^	18.9^n^
**L*****-*****Thr**^4^		
1		174.0
2	4.76^d^	56.6
3	3.99, m	68.9
4	1.17, d (*6.3*)	20.0^l^
***N*****-Me-L*****-*****Val**^5^		
1		172.0
2	4.69, d (*11.0*)	63.6
3	2.24^a^	28.0
4	0.89^e^	19.9^l^
5	0.82, d (*6.5*)	19.1^n^
*N*-Me	3.20, s	31.8^i^
**L*****-*****Pro**^6^		
1		174.8
2	4.80, dd (*8.4, 4.9*)	58.6
3	a 2.21^f^	30.2°
	b 1.66, m	
4	a 2.05^b^	25.8^p^
	b 1.92, m	
5	3.71, m	48.9
***N*****-Me-L*****-*****Val**^7^		
1		171.7
2	4.91, d (*10.6*)	60.2
3	2.28, m	28.2^q^
4	0.90^e^	20.6
5	0.86^g^	18.6
*N*-Me	3.02, s	31.0^m^
***N*****-Me-L*****-*****Val**^8^		
1		171.1^r^
2	5.03, d (*10.6*)	60.1
3	2.20^f^	28.3^q^
4	0.84, d (*6.7*)	20.3
5	0.67, d (*6.7*)	18.2
*N*-Me	2.27, s	30.3°
***N*****-Me-L*****-*****Phe**^9^		
1		172.9
2	5.94, dd (*9.8,5.7)*	55.4
3	3.05, m	35.9
4		138.2
5/9	7.21, m	130.5
6/8	7.28, m	129.7
7	7.24, m	128.2
*N*-Me	2.98, s	31.5^i^
***N*****-Me-L*****-*****Ile**^10^		
1		171.1^r^
2	4.75^d^	62.5
3	2.08, m	33.6
4	a 1.32, m	25.9^p^
	b 1.00, m	
5	0.87^g^	10.7
6	0.96, d (*6.6*)	16.0
*N*-Me	3.05, s	31.7^i^
**L*****-*****Leu**^11^		
1		^nd^
2	4.36, br s	^nd^
3	1.62, m	42.4
4	1.61, m	26.5
5	0.93^c^	23.7
6	0.92^c^	22.3^k^

a−g *Assignments with the same superscript are overlapping*.

h−r *Assignments with the same superscript may be interchanged*.

nd *resonances not detected*.

*Talaropeptide D (5):* white powder; [α]_D_22 −182.9 (c 0.05), MeOH); 1D and 2D NMR (600 MHz, methanol-*d*_4_) see Table 4 and Supplementary Material; HRESI(+)MS *m*/*z* 1417.9052 [(M+Na)^+^] (calcd for C_72_H_122_N_12_O_15_Na, 1417.9045).

**Table 4:** 1D NMR (methanol-*d*_4_) data for talaropeptide D (**5**).

**#**	**δ_H_ m (*J* in Hz)**	**δ_C_**
***N*****-Me-L*****-*****Ala**^1^		
1		174.3
2	5.45, m	51.3
3	1.27, d (*6.9*)	14.8
*N*-Me	3.14, s	31.8^g^
N-COCH_3_		173.6^h^
*N*-COCH_3_	1.99, s	22.5^i^
***N*****-Me-L*****-*****Val**^2^		
1		170.1
2	4.98, d (*10.9*)	61.5
3	2.24, m	28.6
4	0.98, d (*6.5*)	19.9^j^
5	0.82, d (*6.5*)	19.3^k^
*N*-Me	3.01, s	31.1^l^
**L*****-*****Val**^3^		
1		173.7^h^
2	4.63, d (*7.6*)	56.2
3	2.05^a^	31.8^g^
4	0.92^b^	19.8^j^
5	0.93^b^	18.9^m^
**L*****-*****Thr**^4^		
1		173.7^h^
2	4.80^c^	56.4
3	4.01, m	68.8
4	1.16, d (*6.3*)	20.0^j^
**L*****-***Val*		
1		173.9
2	4.21, d (*7.2*)	60.4
3	2.03^a^	31.9^g^
4	0.93^b^	18.8^m^
5	0.92^b^	19.8^j^
***N*****-Me-L*****-*****Val**^5^		
1		172.0
2	4.69, d (*11.0*)	63.6
3	2.21^d^	28.0
4	0.89, d (*6.5*)	19.8^j^
5	0.80, d (*6.5*)	19.2^k^
*N*-Me	3.20, s	31.8^g^
**L*****-*****Pro**^6^		
1		174.8
2	4.80^c^	58.6
3	a 2.21^d^	30.2^n^
	b 1.66^e^	
4	a 2.05^a^	25.8°
	b 1.92, m	
5	3.71, m	48.9
***N*****-Me-L*****-*****Val**^7^		
1		171.7
2	4.91, d (*10.6*)	60.2
3	2.28, m	28.2^p^
4	0.90^b^	18.6^m^
5	0.86^f^	20.6
*N*-Me	3.02, s	31.0^l^
***N*****-Me-L*****-*****Val**^8^		
1		171.0^q^
2	5.03, d (*10.6*)	60.2
3	2.20^d^	28.3^p^
4	0.83, d (*6.7*)	20.3
5	0.67, d (*6.7*)	18.2
*N*-Me	2.27, s	30.3^n^
***N*****-Me-L*****-*****Phe**^9^		
1		172.9
2	5.94, dd (*9.8,5.7)*	55.4
3	3.05, m	35.9
4		138.3
5/9	7.21, m	130.5
6/8	7.28, m	129.7
7	7.24, m	128.2
*N*-Me	2.98, s	31.6^g^
***N*****-Me-L*****-*****Ile**^10^		
1		171.1^q^
2	4.76, d (*11.2*)	62.6
3	2.08, m	33.3
4	a 1.32, m	25.9°
	b 1.00, m	
5	0.87^f^	10.7
6	0.95, d (*6.5*)	16.0
*N*-Me	3.05, s	31.7^g^
**L*****-*****Leu**^11^		
1		^nd^
2	4.32, br s	54.7
3	a 1.65^e^	43.2
	b 1.55, m	
4	1.59, m	26.5
5	0.92^b^	23.8
6	0.91^b^	22.5^i^

a−f *Assignments with the same superscript are overlapping*.

g−q *Assignments with the same superscript may be interchanged*.

nd *resonances not detected*.

### Marfey's analyses

Analyses were carried out following the published method (Vijayasarathy et al., 2016). Briefly, an aliquot (50 μg) of each talaropeptide in 6 M HCl (100 μL) was heated to 100°C in a sealed vial for 12 h, after which the hydrolysate was concentrated to dryness at 40°C under a stream of dry N_2_. The hydrolysate was then treated with 1 M NaHCO_3_ (20 μL) and l-FDAA (1-fluoro-2,4-dinitrophenyl-5-l-alanine amide) as a 1% solution in acetone (40 μL) at 40 °C for 1 h, after which the reaction was neutralized with 1 M HCl (20 μL) and filtered (0.45 μm PTFE) to generate an analyte.

*C*_3_ *Marfey's analysis*. An aliquot (10 μL) of each analyte was subjected to HPLC-DAD-MS analysis (Agilent Zorbax SB-C_3_ column, 5 μm, 4.6 × 150 mm, 50°C, with a 1 mL/min, 55 min linear gradient elution from 15–60% MeOH/H_2_O with a 5% isocratic modifier of 1% formic acid in MeCN) with amino acid content assessed by DAD (340 nm) and ESI(±)MS monitoring, supported by SIE (single ion extraction) methodology, with comparison to authentic standards.

*C*_18_ *Marfey's analysis*. An aliquot (10 μL) of each analyte was subjected to HPLC-DAD analysis for (Agilent Zorbax SB-C_18_ HPLC column, 5 μm, 4.6 × 150 mm, 50 °C, with a 1 mL/min, 50 min isocratic elution of 21% MeOH/H_2_O for *N*-Me-Ala and 34 % MeOH/H_2_O for *N*-Me-Phe, with a 5% isocratic modifier of 1% formic acid in MeCN) with amino acid content assessed by DAD (340 nm), with comparison to authentic standards.

### Genome mining of *Talaromyces* sp. CMB-TU011

Genomic DNA from *Talaromyces* sp. CMB-TU011 was extracted using a standard chloroform protocol (Nikodinovic et al., 2003). The extracted DNA was fragmented using a Covaris focused ultrasonicator and the resulting fragments (~ 1 KB) were used for library construction using a Thrulex DNA-Seq kit (Rubicon Genomics). The library was sequenced using a Next Seq platform in the paired-end (2 × 150) format to yield a total of 6,674,290 reads (1 GB). The raw reads were filtered and trimmed using Trimmomatic v0.36 (Bolger et al., 2014) to yield a total of 5,821,558 high quality reads (0.873 GB), which were assembled using Velvet 1.2.10 (Zerbino and Birney, 2008), Abyss v.2.0.3 (Simpson et al., 2009) and SPAdes v3.11.1 (Bankevich et al., 2012) assemblers with a window of Kmers between 41 and 121, with iterations every 10 units. The best assembly (Velvet with Kmer = 51) was annotated for natural products biosynthetic gene clusters using the Fungal implementation of AntiSMASH 4.0 (Blin et al., 2017). The output was manually curated and domain annotation was improved using pFAM (Finn et al., 2016) and the NCBI Conserved Domain Search tool (Marchler-Bauer et al., 2017). Adenylation domain specificity was predicted using the LSI based A-domain functional predictor (Baranašić et al., 2014). Manual sequence curation was done using the Artemis Genome Browser (Rutherford et al., 2000).

### Antibacterial assay

The bacterium to be tested was streaked onto a tryptic soy agar plate and was incubated at 37°C for 24 h. One colony was then transferred to fresh tryptic soy broth (15 mL) and the cell density was adjusted to 10^4^-10^5^ CFU/mL. The compounds to be tested were dissolved in DMSO and diluted with H_2_O to give 600 μM stock solution (20% DMSO), which was serially diluted with 20% DMSO to give concentrations from 600 μM to 0.2 μM in 20% DMSO. An aliquot (10 μL) of each dilution was transferred to a 96-well microtiter plate and freshly prepared microbial broth (190 μL) was added to each well to give final concentrations of 30−0.01 μM in 1% DMSO. The plates were incubated at 37°C for 24 h and the optical density of each well was measured spectrophotometrically at 600 nm using POLARstar Omega plate (BMG LABTECH, Offenburg, Germany). Each test compound was screened against the Gram-negative bacteria *Escherichia coli* ATCC 11775 and *Pseudomonas aeruginosa* ATCC 10145 and the Gram-positive bacteria *Staphylococcus aureus* ATCC 25923 and *Bacillus subtilis* ATCC 6051. Rifampicin was used as a positive control (40 μg/mL in 10% DMSO). The IC_50_ value was calculated as the concentration of the compound or antibiotic required for 50% inhibition of the bacterial cells using Prism 7.0 (GraphPad Software Inc., La Jolla, CA).

### Antifungal assay

The fungus *Candida albicans* ATCC 10231 was streaked onto a Sabouraud agar plate and was incubated at 37°C for 48 h. One colony was then transferred to fresh Sabouraud broth (15 mL) and the cell density adjusted to 10^4^-10^5^ CFU/mL. Test compounds were dissolved in DMSO and diluted with H_2_O to give a 600 μM stock solution (20% DMSO), which was serially diluted with 20% DMSO to give concentrations from 600 to 0.2 μM in 20% DMSO. An aliquot (10 μL) of each dilution was transferred to a 96-well microtiter plate and freshly prepared fungal broth (190 μL) was added to each well to give final concentrations of 30–0.01 μM in 1% DMSO. The plates were incubated at 37°C for 24 h and the optical density of each well was measured spectrophotometrically at 600 nm using POLARstar Omega plate (BMG LABTECH, Offenburg, Germany). Amphotericin B was used as a positive control (30 μg/ml in 10% DMSO). Where relevant, IC_50_ value were calculated as the concentration of the compound or antifungal drug required for 50% inhibition of the fungal cells using Prism 7.0 (GraphPad Software Inc., La Jolla, CA).

### Cytotoxicity assay

Adherent cell SW620 (human colorectal carcinoma) and NCI-H460 (human lung carcinoma) cells were cultured in Roswell Park Memorial Institute (RPMI) 1640 medium. All cells were cultured as adherent mono-layers in flasks supplemented with 10% fetal bovine serum, l–glutamine (2 mM), penicillin (100 unit/mL), and streptomycin (100 μg/mL), in a humidified 37°C incubator supplied with 5% CO_2_. Briefly, cells were harvested with trypsin and dispensed into 96-well microtiter assay plates at 3,000 cells/well, after which they were incubated for 18 h at 37°C with 5% CO_2_ (to allow cells to attach as adherent mono-layers). Test compounds were dissolved in 20% DMSO in PBS (v/v) and aliquots (10 μL) applied to cells over a series of final concentrations ranging from 10 nM to 30 μM. After 48 h incubation at 37°C with 5% CO_2_ an aliquot (20 μL) of 3-(4,5-dimethylthiazol-2-yl)-2,5-diphenyltetrazolium bromide (MTT) in phosphate buffered saline (PBS, 5 mg/mL) was added to each well (final concentration 0.5 mg/mL), and microtiter plates were incubated for a further 4 h at 37°C with 5% CO_2_. After final incubation, the medium was aspirated and precipitated formazan crystals dissolved in DMSO (100 μL/well). The absorbance of each well was measured at 580 nm with a PowerWave XS Microplate Reader from Bio-Tek Instruments Inc. Where relevant, IC_50_ values were calculated using Prism 7.0, as the concentration of analyte required for 50% inhibition of cancer cell growth (compared to negative controls). Negative control was 1% aqueous DMSO, while positive control was doxorubicin (30 μM). All experiments were performed in duplicate.

### Plasma stability assay

An aliquot of talaropeptide D (**5**) (10 μL, 1 mM in DMSO) was added to rat plasma (190 μL) pooled from >3 different rats, and heated to 37°C in a circulating water bath. Aliquots (20 μL) were taken at time points 0, 60, 120, and 180 min and added to MeCN (80 μL). Samples were centrifuged at 13,000 g for 3 min, and the supernatants concentrated to dryness under N_2_. After resuspending in MeOH (30 μL), and aliquot (1 μL) was analyzed by UHPLC-QTOF (MS), to detect and quantify residual talaropeptide D (**5**).

## Results

### Production and isolation

UHPLC-QTOF analysis of MATRIX cultivations (i.e., microbioreactor well, Figure S1) revealed that YES static broth cultivation was optimum for the production of talaropeptides (Figures S2–S4). Reversed phase HPLC-DAD-ESIMS analysis of a 10 day YES static broth cultivation of CMB-TU011 revealed complete suppression of talarolide A (**1**) biosynthesis, in favor of four new higher molecular weight *putative* peptides, eluting in the order *m/z* 1254.8 (**2**), 1353.8 (**3**), 1318.8 (**4**), and 1417.9 (**5**) (Figure S6). Subsequent studies confirmed the production of **2**–**5** in YES static broth flask cultivations (80 mL broths in 250 mL flasks), with a >10-fold increase in production in flasks sealed with an air permeable cotton plug, as opposed to an air impermeable screw cap (Figure S5). Based on these results, scaled up production (160 mL broth in 10 × 500 mL flasks) successfully yielded a crude EtOAc extract (1.65 g), which was subjected to solvent trituration to yield hexane (495 mg) and MeOH (1.15 g) solubles. With analytical HPLC-DAD-ESIMS localizing **2-5** in the MeOH solubles, this material was subjected to gel chromatography (Sephadex LH-20, MeOH) followed by semi-preparative reversed phase HPLC chromatography, to yield talaropeptides A (**2**, 1.3 mg), B (**3**, 1.3 mg), C (**4**, 1.8 mg), and D (**5**, 2.8 mg) (Figure 1).

### Talaropeptide A (2)

**Figure 2:**
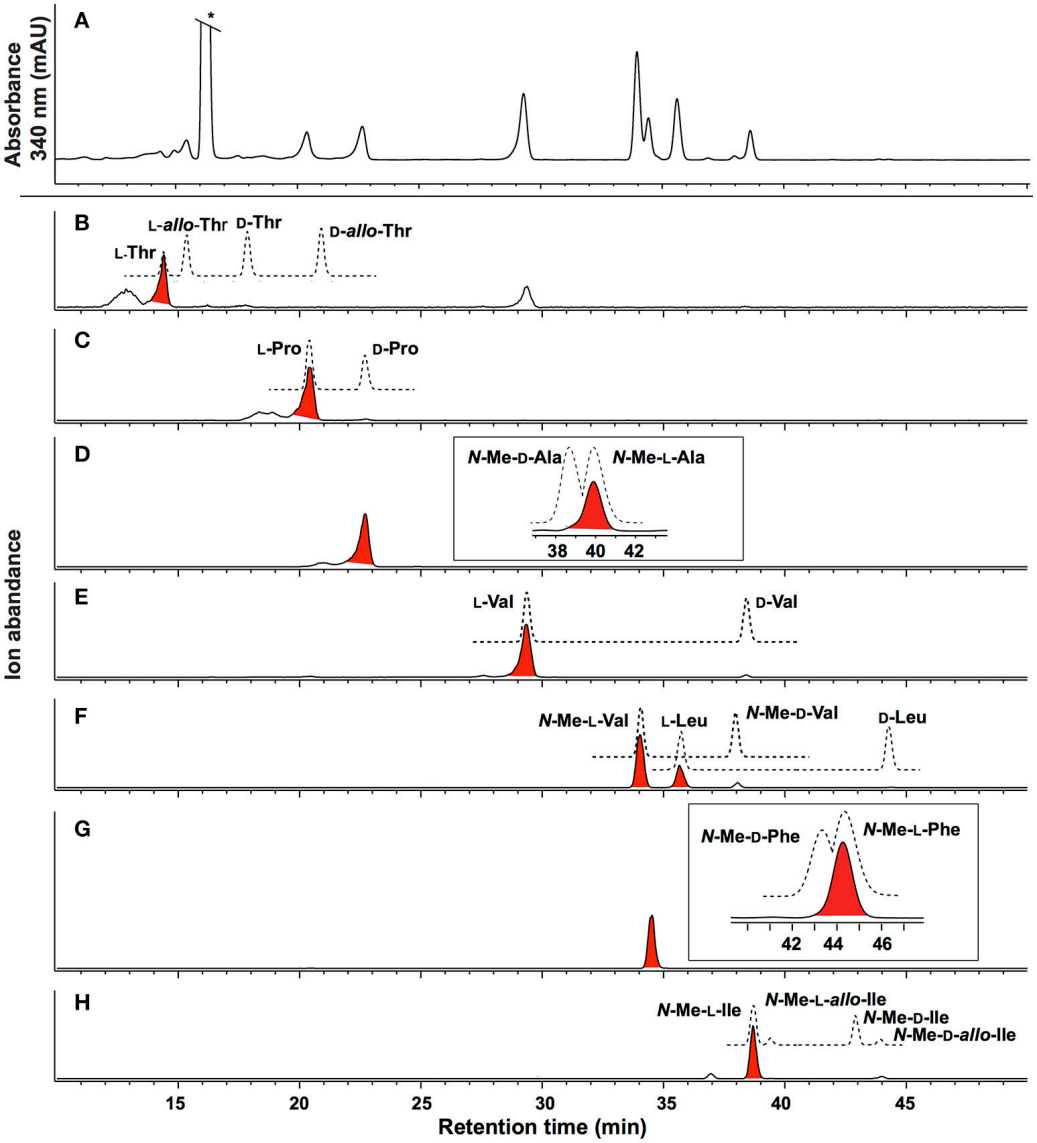
Marfeys analysis for talaropeptide A (**2**). **(A)** C_3_ HPLC-DAD (340 nm) chromatogram of l-FDAA derivatised hydrolysate of 2. **(B–H)** C_3_ HPLC-MS-SIE chromatograms for selected l-FDAA derivatives of amino acid standards (broken lines) and the acid hydrolysate of 2 (red highlighted peaks). The inset in **(D)** and **(G)** show the C_18_ HPLC-DAD chromatogram. Traces reveal **(B)** l-Thr (SIE *m/z* 372), **(C)** l-Pro (SIE *m/z* 368), **(D)** *N*-Me-l-Ala (SIE *m/z* 356) **(E)** l-Val (SIE *m/z* 370) **(F)** *N*-Me-l-Val and l-Leu (SIE *m/z* 384), **(G)** *N*-Me-l-Phe (SIE *m/z* 432) and **(H)** *N*-Me-l-Ile (SIE *m/z* 398). *Residual Marfey's reagent.

**Figure 3:**
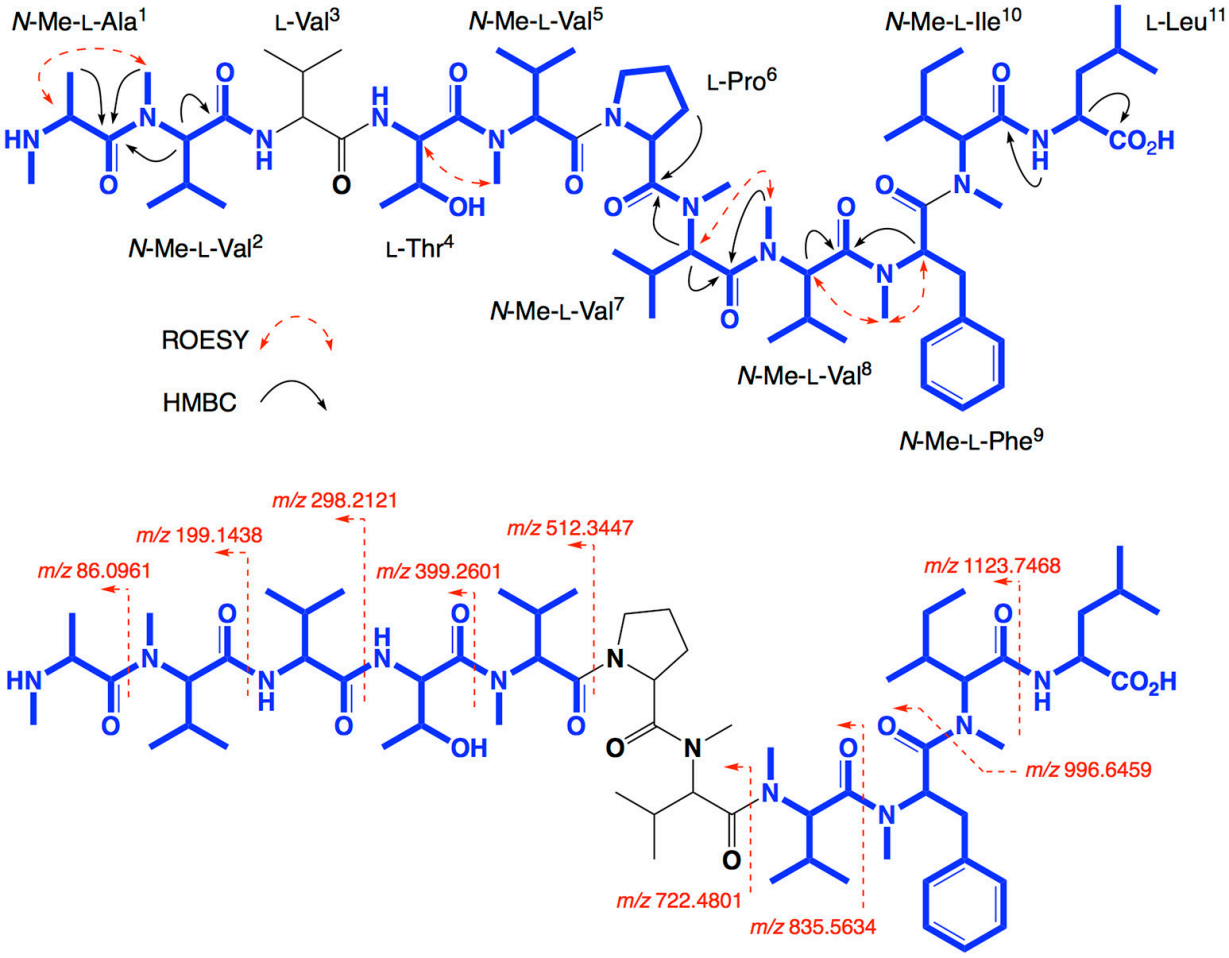
Selected 2D NMR ROESY and HMBC correlations, and MS/MS fragmentations for talaropeptide A (**2**)–partial sequences highlighted in blue.

HRESI(+)MS analysis of **2** returned a protonated molecular ion attributed to a molecular formula (C_65_H_111_N_11_O_13_, Δmmu−0.1) requiring 16 double bond equivalent (DBEs). Consistent with its *putative* peptide status, C_3_ and C_18_ Marfey's analyses (Figure 2), together with careful consideration of 1D and 2D NMR (DMSO-*d*_6_) data (Table 1, Figures S8–S13), confirmed the presence of 11 amino acid residues [L-Thr, L-Pro, L-Val, L-Leu, *N*-Me-L-Ala, *N*-Me-L-Val (×4), *N*-Me-L-Phe and *N*-Me-L-Ile]. Whereas the C_3_ Marfey's method proved very effective at discriminating most amino acids, and in particular l vs d *N*-Me-Ile and *N*-Me-*allo*-Ile (Figure 2H), the C_18_ Marfey's method was needed to discriminate l vs d *N*-Me-Ala (Figure 2D, inset). The presence of multiple (×4) *N*-Me-L-Val residues was apparent from the complex array of isopropyl methyl resonances in the ^1^H NMR data for **2** (Table 1). This assessment of the amino acid content in **2** accounted for all DBE and was indicative of a linear undecapeptide. Although overlapping 1D NMR resonances prevented assignment of the complete amino acid sequence, diagnostic 2D NMR HMBC and ROESY correlations did identify a number of partial sequences; (i) *N*-Me-L-Ala^1^-*N*-Me-L-Val^2^-*N*-H (e.g., an HMBC correlation from *N*-Me-L-Val^2^ to C-1 in *N*-Me-L-Ala^1^); (ii) L-Thr^4^-*N*-Me-L-Val^5^ (e.g., a ROESY correlation between H-2 in L-Thr^4^ and *N*-Me-L-Val^5^); (iii) L-Pro^6^-*N*-Me-L-Val^7^-*N*-Me-L-Val^8^-*N*-Me-L-Phe^9^ (e.g., HMBC correlations from H-2 in *N*-Me-L-Val^7^ to C-1 in L-Pro^6^, from *N*-Me-L-Val^8^ to C-1 in *N*-Me-L-Val^7^, and from H-2 in *N*-Me-L-Phe^9^ to C-1 in *N*-Me-L-Val^8^); (iv) *N*-Me-L-Ile^10^-L-Leu^11^ (e.g., an HMBC correlation from the *N*-H in L-Leu^11^ to C-1 in *N*-Me-L-Ile^10^) (see Figure 3). While the HMBC and ROESY data failed to link fragments (i–iv), or locate L-Val^3^, these issues were ultimately resolved by diagnostic UHPLC-QTOF (MS/MS) fragmentation patterns, which identified two consolidated partial sequences; (v) *N*-Me-L-Ala^1^-*N*-Me-L-Val^2^-L-Val^3^-L-Thr^4^-*N*-Me-L-Val^5^ and (vi) *N*-Me-L-Val^8^-*N*-Me-L-Phe^9^-*N*-Me-L-Ile^10^-L-Leu^11^ (Figure 3, Figure S14). Assembly of the partial sequences i-vi returned the complete structure for talaropeptide A (**2**) as shown (Figure 1).

### Talaropeptide B (3)

**Figure 4:**
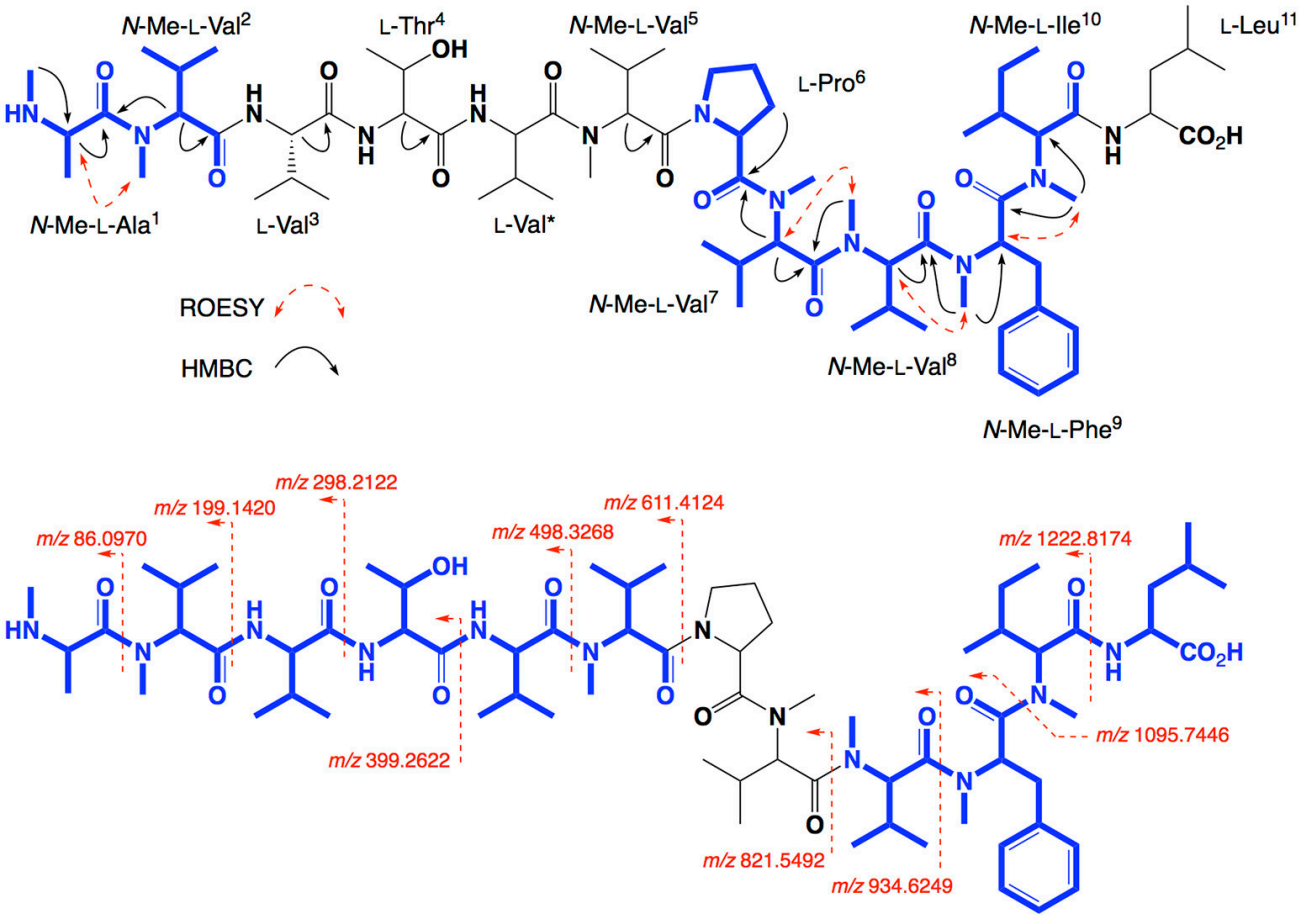
Selected 2D NMR correlations, and MS/MS fragmentations for talaropeptide B (**3**)–partial sequences highlighted in blue.

HRESI(+)MS analysis of **3** returned a sodiated molecular ion attributed to a molecular formula (C_70_H_120_N_12_O_14_, Δmmu−1.9) requiring 17 DBEs, suggestive of a Val homolog of **2**. The ^1^H NMR (DMSO-*d*_6_) spectrum of **3** (Figure S16) revealed resonances closely resembling **2**, however, the extra Val residue was not observed in HSQC (DMSO-*d*_6_) spectrum. Therefore, we re-acquired the NMR data in methanol-*d*_4_, which revealed resonances attributed to the additional Val residue (δ_H_ 3.50, δ_C_ 60.3) (Figure S19). This hypothesis was confirmed by C_3_ and C_18_ Marfey's analyses (Figure S23) and 1D and 2D NMR (methanol-*d*_4_) data (Table 2, Figures S17–S21), which confirmed the presence of 12 amino acid residues [L-Thr, L-Pro, L-Val (×2), L-Leu, *N*-Me-L-Ala, *N*-Me-L-Val (×4), *N*-Me-L-Phe and *N*-Me-L-Ile], accounting for all DBE and requiring that **3** be a linear dodecapeptide. Diagnostic 2D NMR HMBC and ROESY correlations identified key partial sequences; (i) *N*-Me-L-Ala^1^-*N*-Me-L-Val^2^ (e.g., an HMBC correlation from H-2 in *N*-Me-L-Val^2^ to C-1 in *N*-Me-L-Ala^1^); (ii) L-Pro^6^-*N*-Me-L-Val^7^-*N*-Me-L-Val^8^-*N*-Me-L-Phe^9^-*N*-Me-L-Ile^10^ (e.g., HMBC correlations from H-2 in *N*-Me-L-Val^7^ to C-1 in L-Pro^6^, from *N*-Me-L-Val^8^ to C-1 in *N*-Me-L-Val^7^, from *N*-Me-L-Phe^9^ to C-1 in *N*-Me-L-Val^8^, and from *N*-Me-L-Ile^10^ to C-1 in *N*-Me-L-Phe^9^) (Figure 4). Similarly, diagnostic UHPLC-QTOF (MS/MS) fragmentation patterns identified consolidated partial sequence; (iii) *N*-Me-L-Ala^1^-*N*-Me-L-Val^2^-L-Val^3^-L-Thr^4^-L-Val^*^-*N*-Me-Val^5^, and (iv) *N*-Me-L-Val^8^-*N*-Me-L-Phe^9^-*N*-Me-L-Ile^10^-L-Leu^11^ (Figure 4, Figure S22). Assembly of the partial sequences i-iv returned the complete structure for talaropeptide B (**3**) as shown (Figure 1), with the following caveat. As *N*-Me-Val^5^ and L-Leu^11^ are isomeric, their relative position cannot be determined by MS/MS fragmentation (or by way of overlapping 1D NMR resonances). To establish the regiochemistry of these amino acid residues, we draw on biosynthetic comparisons to the co-metabolite **2**, as well as knowledge of the talaropeptide biosynthetic gene cluster (see below).

### Talaropeptide C (4)

**Figure 5:**
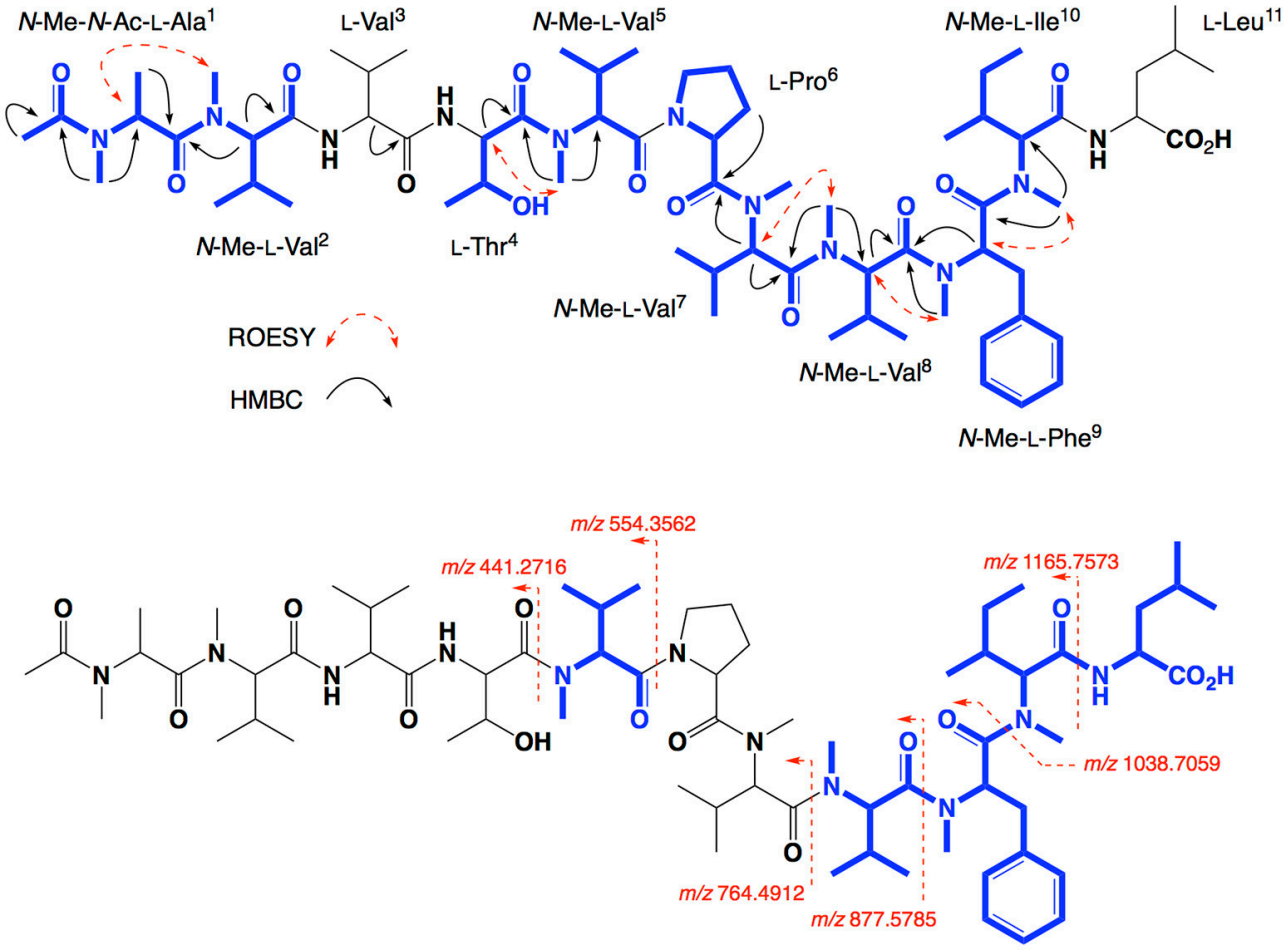
Selected 2D NMR correlations, and MS/MS fragmentations for talaropeptide C (**4**)–partial sequences highlighted in blue.

HRESI(+)MS analysis of **4** returned a sodiated molecular ion attributed to a molecular formula (C_67_H_113_N_11_O_14_, Δmmu +1.5) requiring 17 DBEs, suggestive of an acetylated homolog of **2** (i.e., +42 Da). Comparison of the ^1^H NMR (methanol-*d*_6_) data for **4** with **2** supported the latter hypothesis, with the only significant difference being the appearance of resonances attributed to an acetyl moiety (δ_H_ 1.99, δ_C_ 22.3), with an HMBC correlation from *N*-Me-L-Ala^1^ to *N*-COCH_3_ being diagnostic for an *N*-terminal acetamide moiety. C_3_ and C_18_ Marfey's analyses (Figure S31) together with 1D and 2D NMR (methanol-*d*_4_) data (Table 3, Figures S24–S29), confirmed the presence of 11 amino acid residues (L-Thr, L-Pro, L-Val, L-Leu, *N*-Me-L-Ala, *N*-Me-L-Val (×4), *N*-Me-L-Phe and *N*-Me-L-Ile), accounting for all DBE and requiring that **4** be a linear undecapeptide. Diagnostic 2D NMR HMBC and ROESY correlations identified key partial sequences; (i) *N*-Me-*N*-Ac-L-Ala^1^-*N*-Me-L-Val^2^ (e.g., an HMBC correlation from H-2 in *N*-Me-L-Val^2^ to C-1 in *N*-Me-*N*-Ac-L-Ala^1^); (ii) L-Thr^4^-*N*-Me-L-Val^5^ (e.g., an HMBC correlation from an *N*-Me-L-Val^5^ to C-1 in L-Thr^4^), and (iii) L-Pro^6^-*N*-Me-L-Val^7^-*N*-Me-L-Val^8^-*N*-Me-L-Phe^9^-*N*-Me-L-Ile^10^ (e.g., HMBC correlations from H-2 in *N*-Me-L-Val^7^ to C-1 in L-Pro^6^, and from *N*-Me-L-Val^8^ to C-1 in *N*-Me-L-Val^7^, and from *N*-Me-L-Phe^9^ to C-1 in *N*-Me-L-Val^8^, and from *N*-Me-L-Ile^10^ to C-1 in *N*-Me-L-Phe^9^) (Figure 5). Similarly, diagnostic UHPLC-QTOF (MS/MS) fragmentation patterns identified the consolidated partial sequence (iv) *N*-Me-L-Val^8^-*N*-Me-L-Phe^9^- *N*-Me-L-Ile^10^-L-Leu^11^ (Figure 5, Figure S30). Assembly of the partial sequences i–iv returned the complete structure for talaropeptide C (**4**) as shown (Figure 1).

### Talaropeptide D (5)

**Figure 6:**
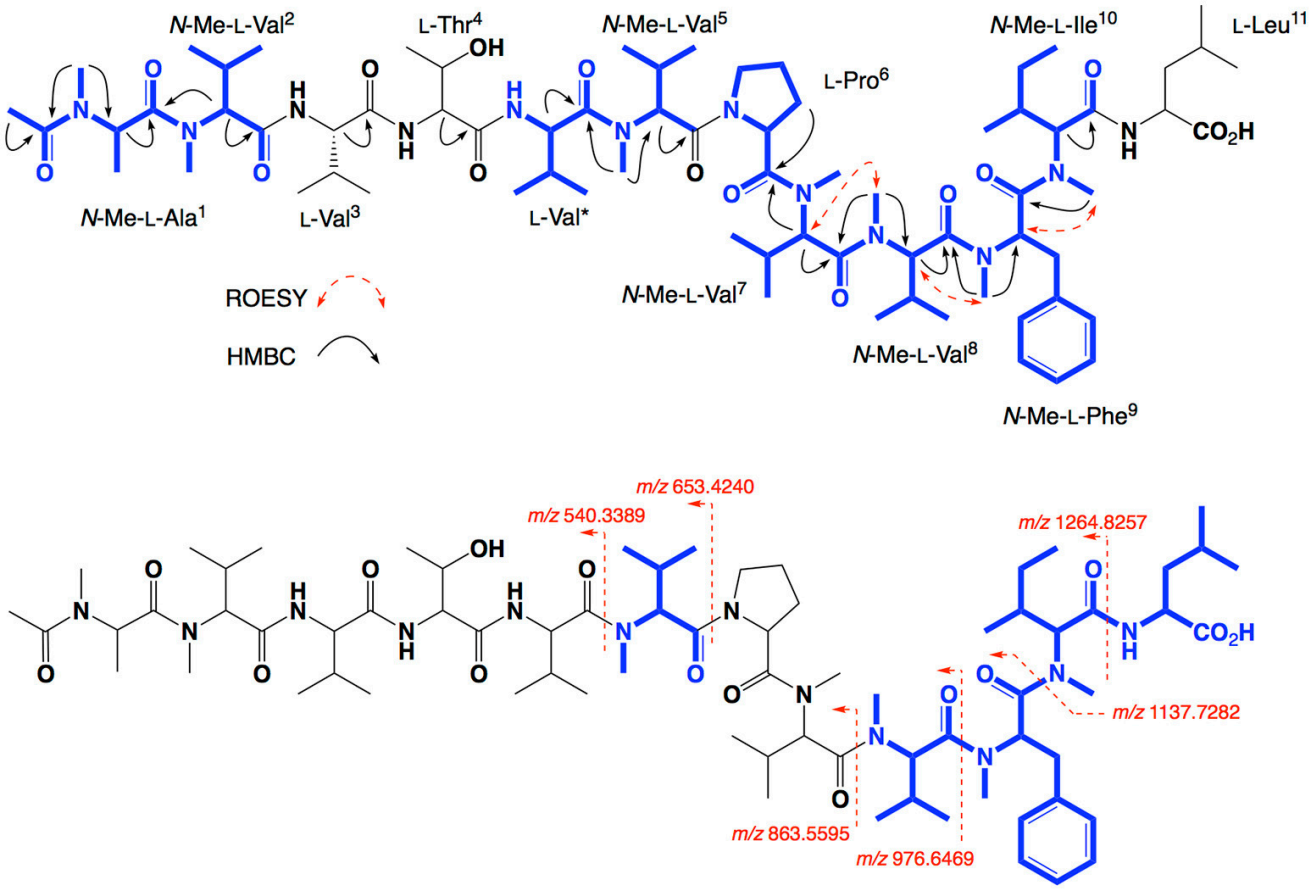
Selected 2D NMR correlations, and MS/MS fragmentations for talaropeptide D (**5**)–partial sequences highlighted in blue.

HRESI(+)MS analysis of **5** returned a sodiated molecular ion attributed to a molecular formula (C_72_H_122_N_12_O_15_, Δmmu +0.7) requiring 18 DBEs, suggestive of an acetylated homolog of **3** (i.e., +42 Da). Comparison of the ^1^H NMR (methanol-*d*_4_) data for **5** with **3** supported the latter hypothesis, with the only significant difference being the appearance of resonances attributed to an acetyl moiety (δ_H_ 1.99, δ_C_ 22.5), with HMBC correlations to the *N*-Me-L-Ala^1^ position it on the *N*-terminus. C_3_ and C_18_ Marfey's analyses (Figure S38) and 1D and 2D NMR (methanol-*d*_6_) data (Table 4, Figures S32–S36), confirmed the presence of 12 amino acid residues [L-Thr, L-Pro, L-Val (×2), L-Leu, *N*-Me-L-Ala, *N*-Me-L-Val (×4), *N*-Me-L-Phe and *N*-Me-L-Ile], accounting for all DBE and requiring that **5** be a linear dodecapeptide. Diagnostic 2D NMR HMBC and ROESY correlations identified key partial sequences; (i) *N*-Me-*N*-Ac-L-Ala^1^-*N*-Me-L-Val^2^ (e.g., HMBC correlations from *N*-Me-L-Ala^1^ to *N*-COCH_3_, and from H-2 in *N*-Me-L-Val^2^ to C-1 in *N*-Me-L-Ala^1^); (ii) L-Val^*^-*N*-Me-L-Val^5^ (e.g., an HMBC correlation from an *N*-Me-L-Val^5^ to C-1 in L-Val^*^), and (iii) L-Pro^6^-*N*-Me-L-Val^7^-*N*-Me-L-Val^8^-*N*-Me-L-Phe^9^-*N*-Me-L-Ile^10^ (e.g., HMBC correlations from H-2 in *N*-Me-L-Val^7^ to C-1 in L-Pro^6^, and from *N*-Me-L-Val^8^ to C-1 in *N*-Me-L-Val^7^, and from *N*-Me-L-Phe^9^ to C-1 in *N*-Me-L-Val^8^, and from an *N*-Me to C-1 in *N*-Me-L-Phe^9^) (see Figure 6). Similarly, diagnostic UHPLC-QTOF (MS/MS) fragmentation patterns identified the partial sequence (iv) *N*-Me-L-Val^8^-*N*-Me-L-Phe^9^-*N*-Me-L-Ile^10^-L-Leu^11^ (Figure 6, Figure S37). Assembly of the partial sequences i–iv returned the complete structure for talaropeptide D (**5**) as shown (Figure 1), with the following caveat. As the NMR and MS/MS data for **5** could not provide an experimental assignment of relative regiochemistry for the dipeptide fragment comprised of L-Val^3^ and L-Thr^4^, we draw on biosynthetic comparisons to the co-metabolite **3**, as well as knowledge of the talaropeptide biosynthetic gene cluster (see below).

### Talaropeptide biosynthesis

A genome sequence of *Talaromyces* sp. CMB-TU011 was obtained, with coverage of 31X length of 27.5 MB, and a GC content of 47 %, consistent with related species (Table 5). Natural product genome mining of this sequence identified 17 biosynthetic gene clusters (BGCs, Table S7) including three non-ribosomal peptide synthetases (NRPS). A very large intron-less mega synthase that includes 12 modules and 44 domains encoded in a single gene (45,892 bases, 15,297 amino acids) was identified as a plausible talaropeptide NRPS. Of note, this NRPS is only 3.2 kb smaller than the largest NRPS ever reported (49,104 bases, plu2670, 16,367 amino acids), being that documented for the extensively *N*-methylated and commercially important fungal cyclic undecapeptide cyclosporine from *Tolypocladium inflatum* (GenBank accesion: CAA82227, 15281 amino acids) (Weber et al., 1994).

**Table 5:** Draft genome sequence of *Talaromyces* sp. CMB-TU011.

Length	27,481,273
GC content	0.47%
Coverage	31X
Contigs	1652
Closest relative (ITS as marker)	*Talaromyces helicus* (99% sequence identity)
Biosynthetic gene clusters	17

**Figure 7:**
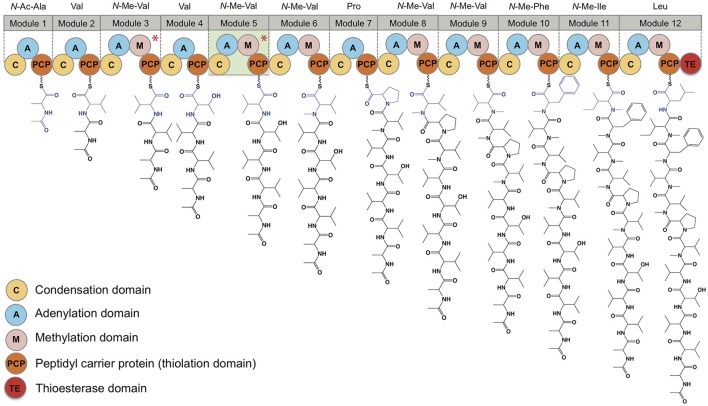
Domain organization of the talaropeptide synthase and biosynthetic logic of the talaropeptides. Biosynthesis of talaropeptide D (**5**) is depicted. Methylation domains marked with an asterisk are skipped during biosynthesis, while module 5 (highlight light green) is skipped during talaropeptides A (**2**) and C (**4**) biosynthesis.

The putative talaropeptide NRPS (Figure 7) exhibits an *N*-terminus condensation domain with a similar configuration to that of previously reported C domains associated with peptides incorporating *N*-terminal acyl moieties, consistent with the *N*-terminal *N*-acylation observed in talaropeptides C (**4**) and D (**5**). This domain might have been skipped during the biosynthesis of talaropeptide A (**2**) and B (**3**), or alternatively the *N*-Ac moiety may have been deleted after the biosynthesis (*i.e*. hydrolysed). A total of 12 adenylation domains were detected, in agreement with the number of amino acid residues found in talaropeptides B (**3**) and D (**5**). Predicted amino acid specificities for these domains are largely in agreement with those observed for **3** and **5**, except for modules 1 and 4 (Table 6). Seven methyl transferase domains were consistent with *N*-methylation sites in **2**-**5**, with exceptions for *N*-methylation of residues 1 and 2, which may be installed post NRPS assembly. Alternatively, the methyl transferase in module 3 appears to be inactive on its corresponding extension step (i.e., L-Val^3^), and may be responsible for methylation of the first two residues (i.e., *N*-Me-*N*-Ac-L-Ala^1^ and *N*-Me-L-Val^2^). The methylation domain at module 5 (L-Val^*^) appears to be inactive during the biosynthesis of talaropeptides B (**3**) and D (**5**), while the entire module 5 inactive in the biosynthesis of talaropeptides A (**2**) and C (**4**). Finally, a thioesterase domain was detected at the C-terminus of the talaropeptide NRPS, accounting for the release of the peptide product with a C-terminus carboxylic acid.

**Table 6:** Comparison of a predicted product for ORFX (talaropeptide synthase) with structure for talaropeptide D (**5**). Adenylation (A) domain specificity was calculated using the LSI based A-domain functional predictor.

**Module**	**Residue Prediction**	**Domain LSI score**	**Residues in 5**	**Comment**
1	*N*-Ac-L-Val	0.585	*N*-Ac-*N*-Me-L-Ala^1^	Adenylation domain promiscuity or new sequence motif. Post-NRPS methylation. Acylation (starter C domain) skipped for talaropeptides A (**2**) and B (**3**).
2	L-Val	0.493	*N*-Me-L-Val^2^	Post-NRPS methylation.
3	*N*-Me-L-Val	0.673	L-Val^3^	Skipped methylation domain.
4	L-Val	0.673	L-Thr^4^	Adenylation domain promiscuity or new sequence motif.
5	*N*-Me- L-Val	0.628	L-Val*	Skipped methylation domain. Module skipped for talaropeptides A (**2**) and C (**4**).
6	*N*-Me-L-Val	0.673	*N*-Me-L-Val^5^	Matched prediction.
7	L-Val/L-Pro	0.49/0.366	L-Pro^6^	Partially matched prediction.
8	*N*-Me-L-Val	0.673	*N*-Me-L-Val^7^	Matched prediction.
9	*N*-Me-L-Val	0.673	*N*-Me-L-Val^8^	Matched prediction.
10	*N*-Me-L-Phe	0.482	*N*-Me-L-Phe^9^	Matched prediction.
11	*N*-Me-L-Ile	0.657	*N*-Me-L-Ile^10^	Matched prediction.
12	L-Leu	0.739	L-Leu^11^	Matched prediction.

### Talaropeptide biological activity

Talaropeptides A-D (**2**-**5**) exhibited no growth inhibitory activity when tested (up to 30 μM) against human lung (NCI-H460) and colon (SW620) carcinoma cells, or when tested against the Gram-negative bacteria *Escherichia coli* ATCC 11775 and *Pseudomonas aeruginosa* ATCC 10145, the Gram-positive bacteria *Staphylococcus aureus* ATCC 25923 and *S. aureus* ATCC 9144, or the fungus *Candida albicans* ATCC 10231 (Supplementary Material). By contrast, talaropeptides A (**2**) and B (**3**) alone exhibited promising growth inhibitory activity (IC_50_ 1.5 and 3.7 μM) against the Gram-positive bacteria *Bacillus subtilis* ATCC 6633 (Figure S41). As might be predicted for an extensively *N*-methylated linear peptide, talaropeptide A (**2**) proved stable to rat plasma (i.e., proteases) (Figure S39).

## Discussion

Although fungi are well-known to produce cyclic peptides, linear peptides > 7 amino acid residues are comparatively rare (Komatsu et al., 2001; Boot et al., 2006). For example, excluding peptaibols such as the recently described trichodermides (Jiao et al., 2018), which are dominated by non-proteinogenic amino acids [e.g., α-aminoisobutyric acid (Aib) and d–isovaline (d–Iva)], only a handful linear peptides of > 7 amino acid residues have been reported from fungi. Interestingly, these reports feature peptides from marine-derived fungi, including the dodecapeptide dictyonamides A and B from a marine red alga-derived fungus (Komatsu et al., 2001), and *N*-methylated octapeptides RHM 1 and RHM 2 from a marine sponge-derived *Acremonium* (Boot et al., 2006). Also of note, no linear peptides have been reported from the genus *Talaromyces*.

The talaropeptides A-D (**2**-**5**) represent a new class of extensively *N*-methylated linear peptide natural product, and at the same time feature peptide amino acid sequences that are unprecedented in the scientific literature. That the talaropeptide pharmacophore lacks mammalian cell cytotoxicity, and exhibits highly selective antibacterial properties (albeit with modest potency), with a clear structure activity relationship requirement built around *N*-terminal acetylation, is intriguing.

From an ecological perspective, the link between antibacterial activity and acetylation suggests that control of *N*-acetylation, perhaps as a post-NRPS modification by hydrolysis of the acetyl group or by an unknown biosynthetic mechanism that lead to domain skipping, may bias production in favor of **2** and **3** as an antibacterial defense, or **4** and **5** as putative antibacterial prodrugs. In an ecological setting rich in microbial competitors, this putative biosynthetic mechanism of control may be mediated by inter-species or even inter-kingdom chemical communication.

The discovery that talaropeptide production was highly culture media and phase dependent (i.e., YES broth, static flask with an air permeable seal) raises the possibility that, the paucity of published fungal linear peptides may be due to a bias for cultivation conditions that disfavor linear peptides. Our application of systematic miniaturized microbioreactor approach to trialing cultivation conditions (i.e., MATRIX) provides a low cost, practical means to access this silent potential.

## Data availability statement

The raw data supporting the conclusions of this manuscript will be made available by the authors, without undue reservation, to any qualified researcher.

The GeneBank accession; Bankit212474, talarolide_synthase – MH479449.

## Author contributions

RC initiated and oversaw all research. PD performed all fungal cultivations, and isolated and characterized talaropeptides. PD, PP, AS, ZK, and RC performed data analysis and talaropeptide structure elucidations. ZK isolated fungal DNA, and together with PD carried out bioassays. PC-M and EM performed all genomic analyses, and identified the talaropeptide NRPS. RC and PD co-drafted the manuscript.

### Conflict of interest statement

The authors declare that the research was conducted in the absence of any commercial or financial relationships that could be construed as a potential conflict of interest.

## Supplementary material

Supplementary Material The includes fungal cultivation trial details, as well as tabulated and annotated 1D and 2D NMR data and spectra, C_3_ and C_18_ Marfey's and MS/MS analyses, and biological assay data for **2**–**5**.

## Abbreviations

UHPLC: ultra-high-performance liquid chromatography
HPLC: high-performance liquid chromatography
QTOF: quadrupole time of flight
ESI: electrospray ionization
SIE: single ion extraction
DAD: diode array detector
NRPS: non-ribosomal peptide synthetase
LSI: Latent semantic indexing
ORFX: orphan protein/enzyme.
