# Supplementary material for: Talaropeptides A-D: Structure and Biosynthesis of Extensively N-methylated Linear Peptides From an Australian Marine Tunicate-Derived Talaromyces sp
Source: Front Chem. 2018 Sep 4;6:394. doi: 10.3389/fchem.2018.00394 (PMC6131563; doi:10.3389/fchem.2018.00394)
Supplement: Supplementary Material — The includes fungal cultivation trial details, as well as tabulated and annotated 1D and 2D NMR data and spectra, C3 and C18 Marfey's and MS/MS analyses, and biological assay data for 2–5. [file Data_Sheet_1.pdf]

Supplementary Material

Talaropeptides A-D: Structure and biosynthesis of extensively *N*-methylated linear peptides from an Australian marine tunicate-derived *Talaromyces* sp.

Pradeep Dewapriya,[1] Zeinab G. Khalil,[1] Pritesh Prasad,[1] Angela A. Salim,[1] Pablo Cruz-Morales,[2,3] Esteban Marcellin[2] and Robert J. Capon[1*]

[1]Institute for Molecular Bioscience, The University of Queensland, St Lucia, QLD, Australia

[2]Australian Institute for Bioengineering and Nanotechnology, The University of Queensland, St Lucia, QLD, Australia

[3]Joint BioEnergy Institute, Emeryville, CA, USA.

*** Correspondence:** Professor Robert Capon: r.capon@uq.edu.au

1 Analytical (MATRIX) cultivation of *Talaromyces* sp. CMB-TU011

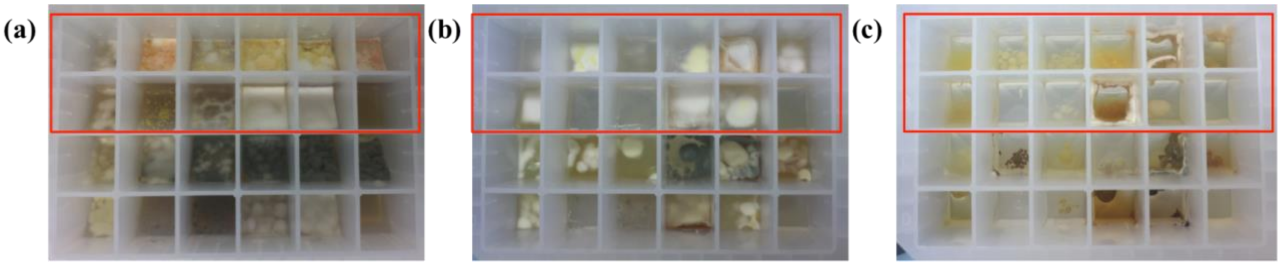

Figure S1. Photographs of 24-well microbioreactors (a) solid agar (b) broth static (c) broth shaken inoculated with *Talaromyces* sp. CMB-TU011 (highlighted in red).

Table S1. Different culture media and conditions used for the matrix analysis

	Culture media at 26 °C				
Solid agar		Broth Static		Broth Shaken (190 rpm)	
i	M1 agar	xii	M1 broth	xxiii	M1 broth
ii	ISP-2 agar	xii	ISP-2 broth	xxiv	ISP-2 broth
iii	ISP-4-agar	xiv	*PY broth	xxv	*PY broth
iv	Potato dextrose agar	xv	Potato dextrose broth	xxvi	Potato dextrose broth
v	YES agar	xvi	YES broth	xxvii	YES broth
vi	Sabouraud dextrose agar	xvii	Sabouraud dextrose broth	xxviii	Sabouraud dextrose broth
vii	Tryptic soy agar	xviii	Tryptic soy broth	xxix	Tryptic soy broth
viii	Basal agar	xix	Basal broth	xxx	Basal broth
ix	Czapeck agar	xx	Czapeck broth	xxxi	Czapeck broth
x	M2 agar	xxi	M2 broth	xxxii	M2 broth
xi	*PYG agar	xxii	*PYG broth	xxxiii	*PYG broth

*PY = peptone and yeast extract

*PYG = peptone, yeast extract and glucose

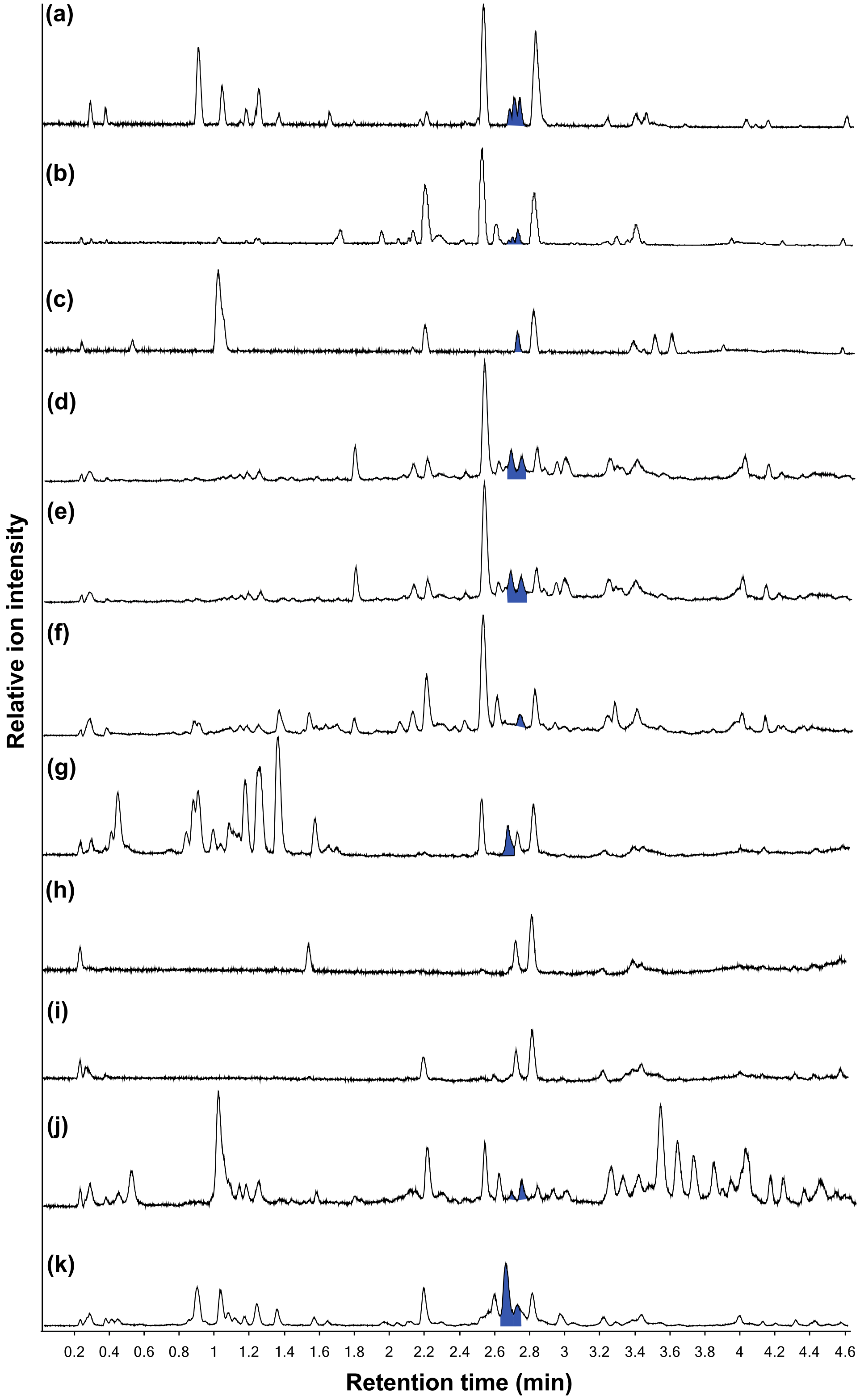

Figure S2. UHPLC-QTOF (MS) total ion chromatograms of the EtOAc crude extracts of CMB-TU011 cultured on solid agar media. (a) M1; (b) ISP-2; (c) ISP-4; (d) PDA; (e) YES; (f) SDA; (g) TSA; (h) Basal; (i) Czapeck; (j) M2 and (k) PYG.

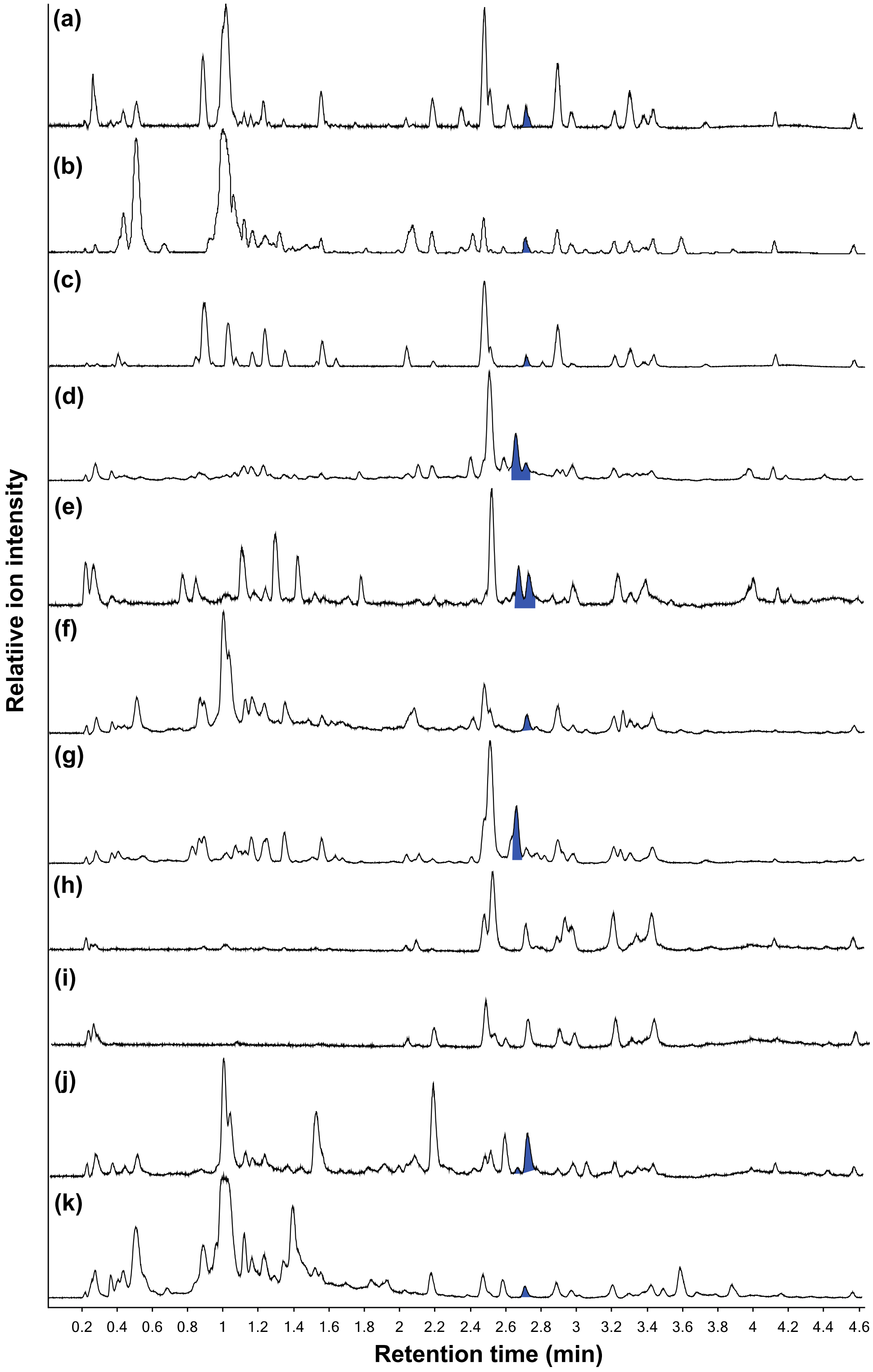

Figure S3. UHPLC-QTOF (MS) total ion chromatograms of the EtOAc crude extracts of CMB-TU011 cultured on liquid shaking condition. (a) M1; (b) ISP-2; (c) PY; (d) PDA; (e) YES; (f) SDA; (g) TSA; (h) Basal; (i) Czapeck; (j) M2 and (k) PYG

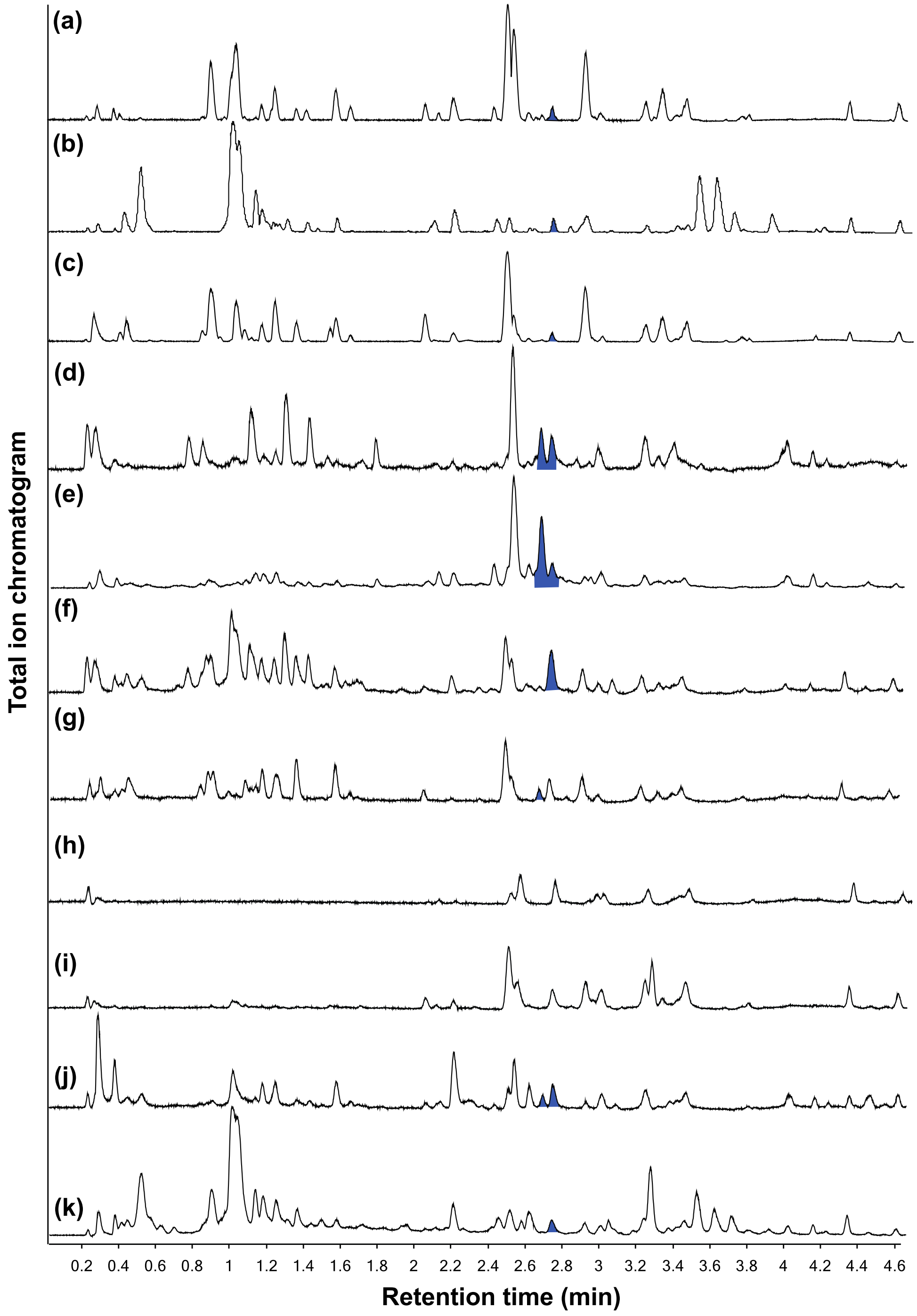

Figure S4. UHPLC-QTOF (MS) total ion chromatograms of the EtOAc crude extracts of CMB-TU011 cultured on liquid static. (a) M1; (b) ISP-2; (c) PY; (d) PDA; (e) YES; (f) SDA; (g) TSA; (h) Basal; (i) Czapeck; (j) M2 and (k) PYG.

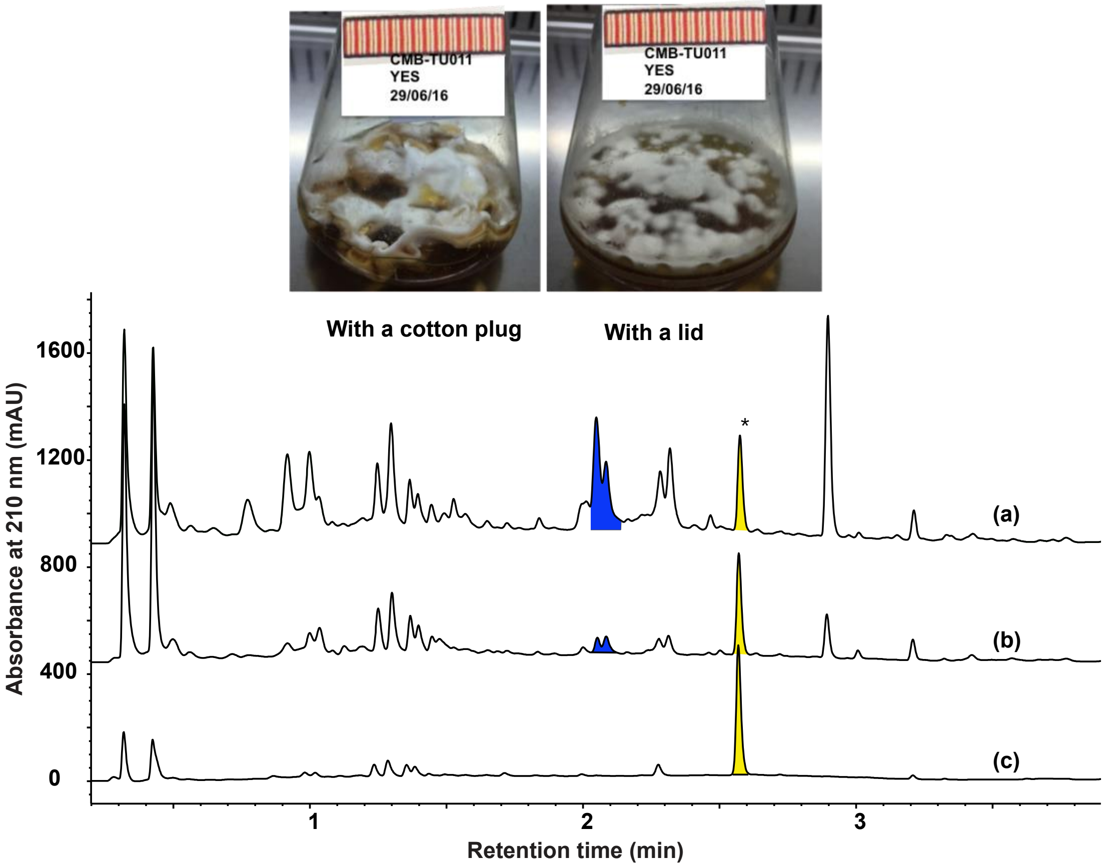

Figure S5. UHPLC-DAD chromatograms at 210 nm of *Talaromyces* sp. CMB-TU011 crude extracts from (a) YES broth static with an air permeable cotton plug (image inset on the left), (b) an air impermeable screw cap (image inset on the right) (c) YES media blank. The metabolites of interest are highlighted in blue. *Internal calibrant.

2 Scale-up cultivation of *Talaromyces* sp. CMB-TU011 and isolation of 2-5

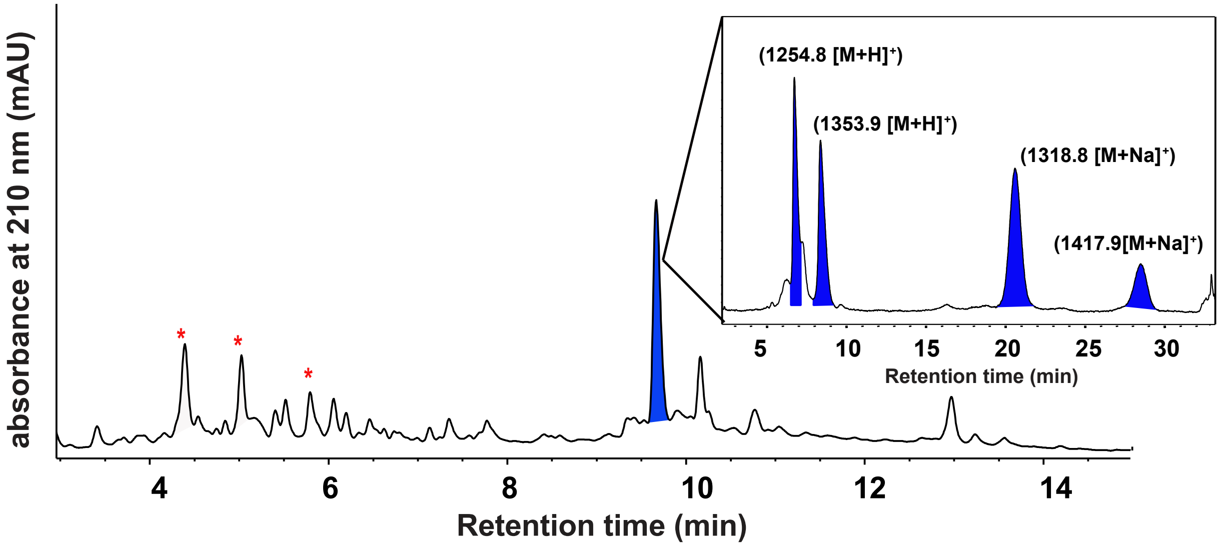

Figure S6. HPLC-DAD (210 nm) chromatogram of the EtOAc crude extract of *Talaromyces* sp. CMB-TU011 cultured on YES static broth eluted using Agilent Zorbax SB-C_8 column, with analytical gradient 90% H_2O/MeCN – 100% MeCN inclusive of an isocratic 0.05% HCO_2H. Inset shows four main peaks resolved by HPLC method using Zorbax SB-C_3 column, with isocratic 40% MeCN inclusive of an isocratic 0.05% HCO_2H modifier. *Media background.

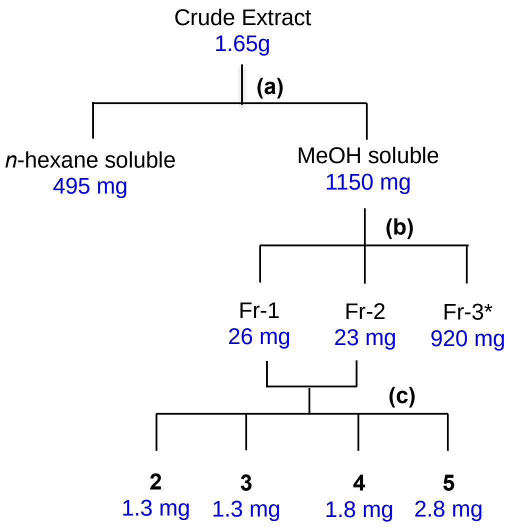

Scheme S1. Isolation scheme for CMB-TU011. (a) *n*-hexane/MeOH solvent partitioning, (b) gel chromatography (Sephadex® LH-20, MeOH) (c) Semi-preparative HPLC (Zorbax SB-C_3 column (9.4 mm × 25 cm) column, with isocratic 40% MeCN/H_2O elution at 3.0 mL/min inclusive of an isocratic 0.01% TFA modifier). *This weight accounts for other fractions.

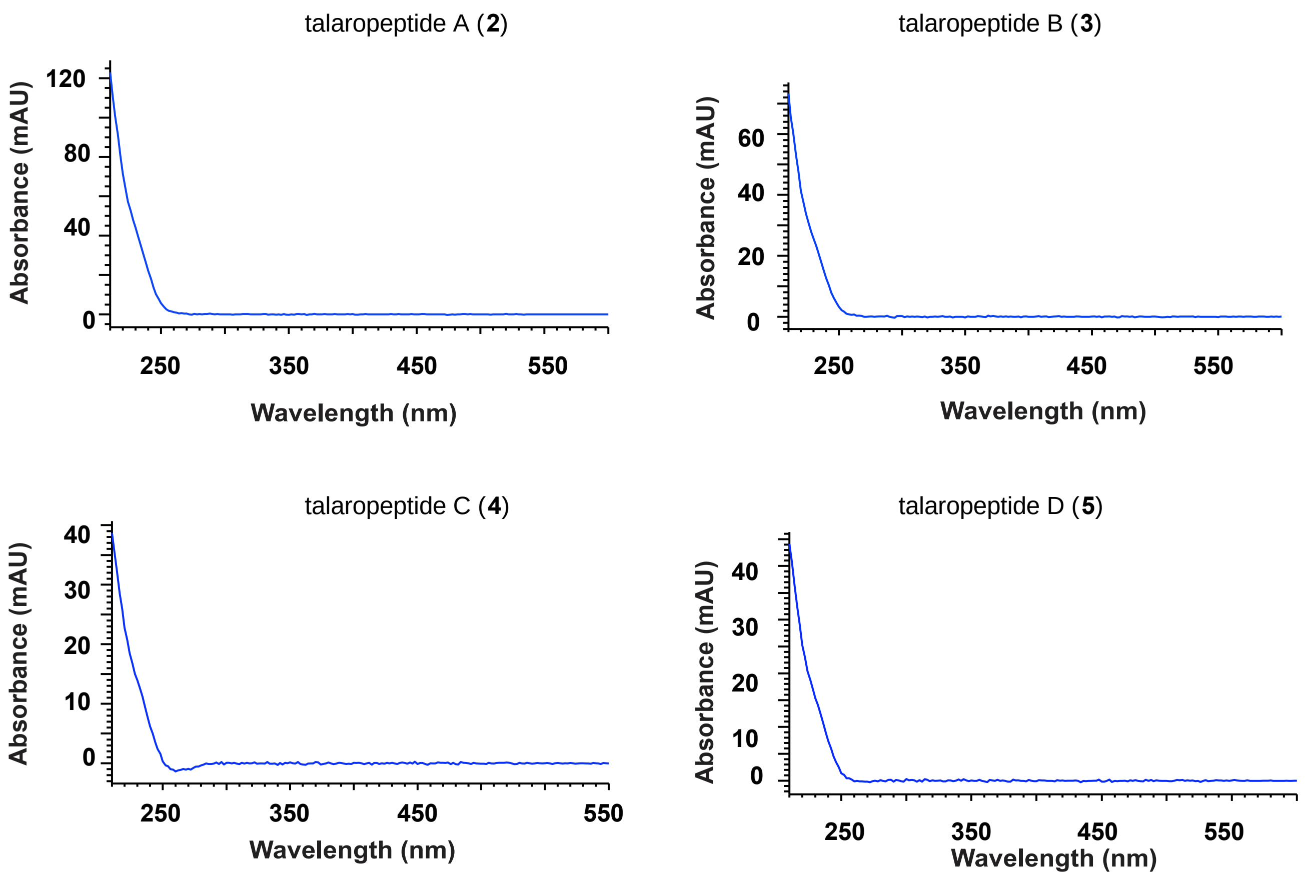

Figure S7. HPLC-DAD (H_2O/MeCN inclusive of 0.05% formic acid) spectra for talaropeptide A – D (**2** – **5**)

3 Talaropeptide A (2)

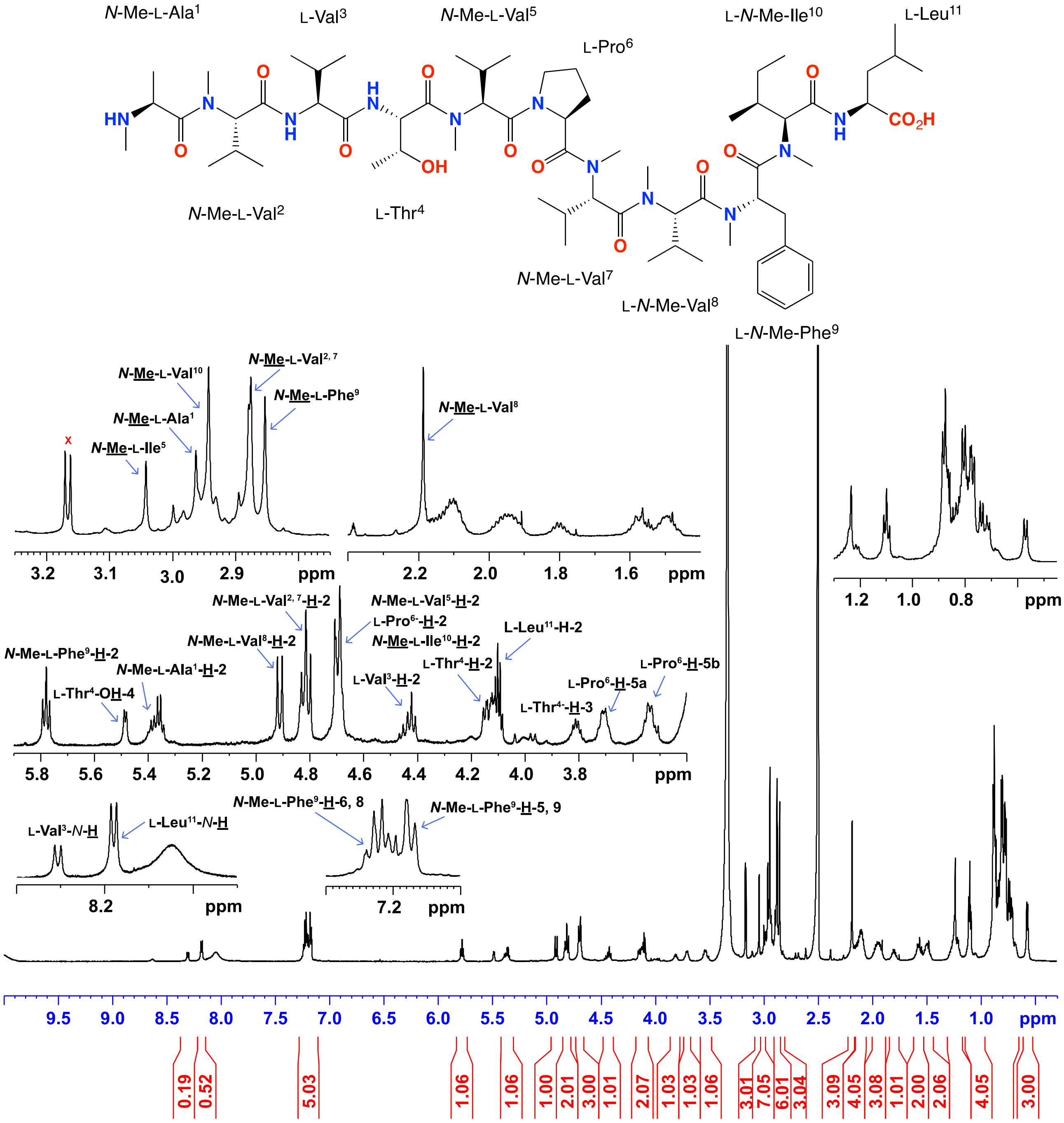

Figure S8. ^{1}H NMR (600 MHz, DMSO-d_6) spectrum of talaropeptide A (**2**).^{x} impurities

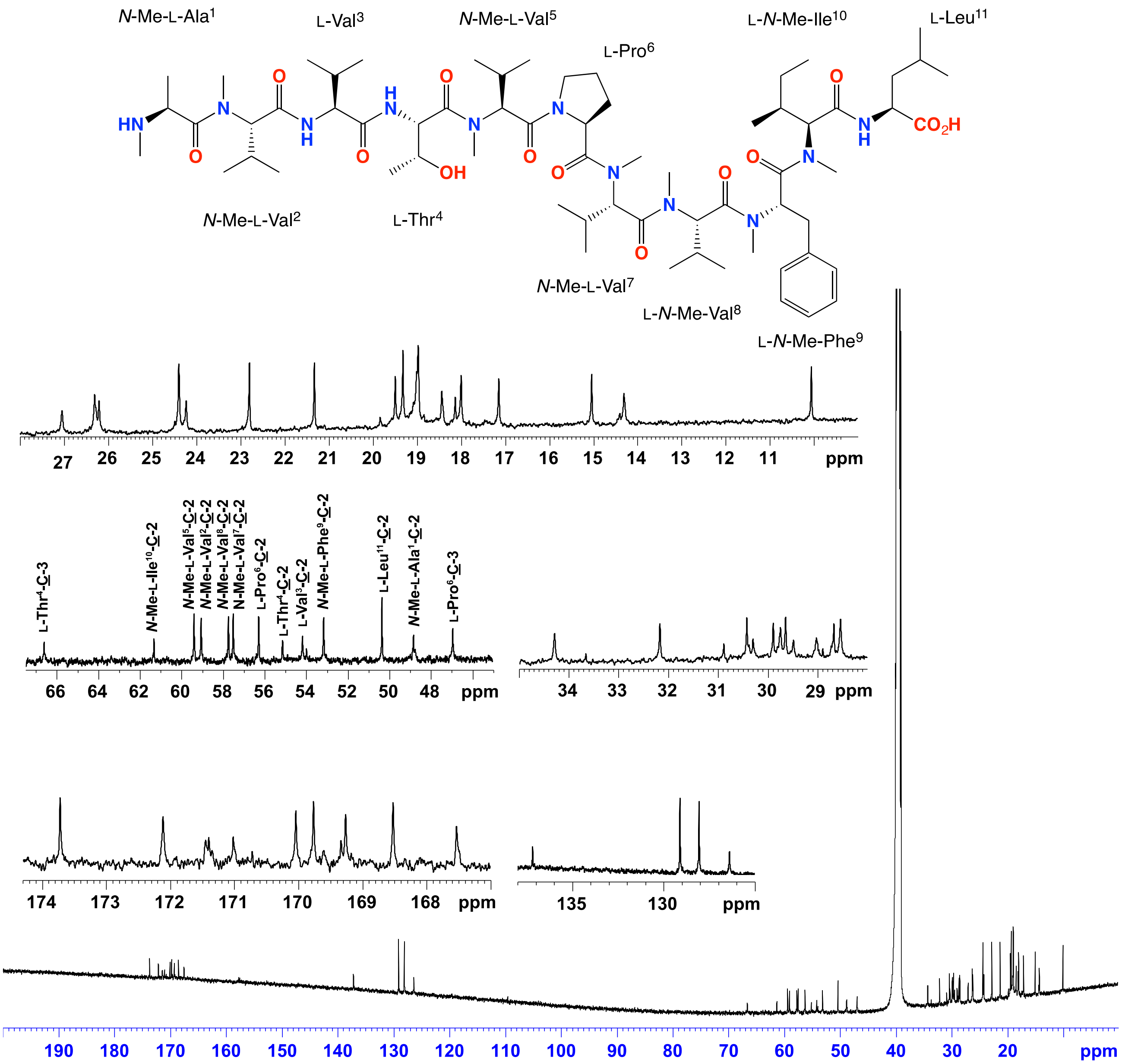

Figure S9: ^{13}C NMR (150 MHz, DMSO-d_6) spectrum of talaropeptide A (**2**).

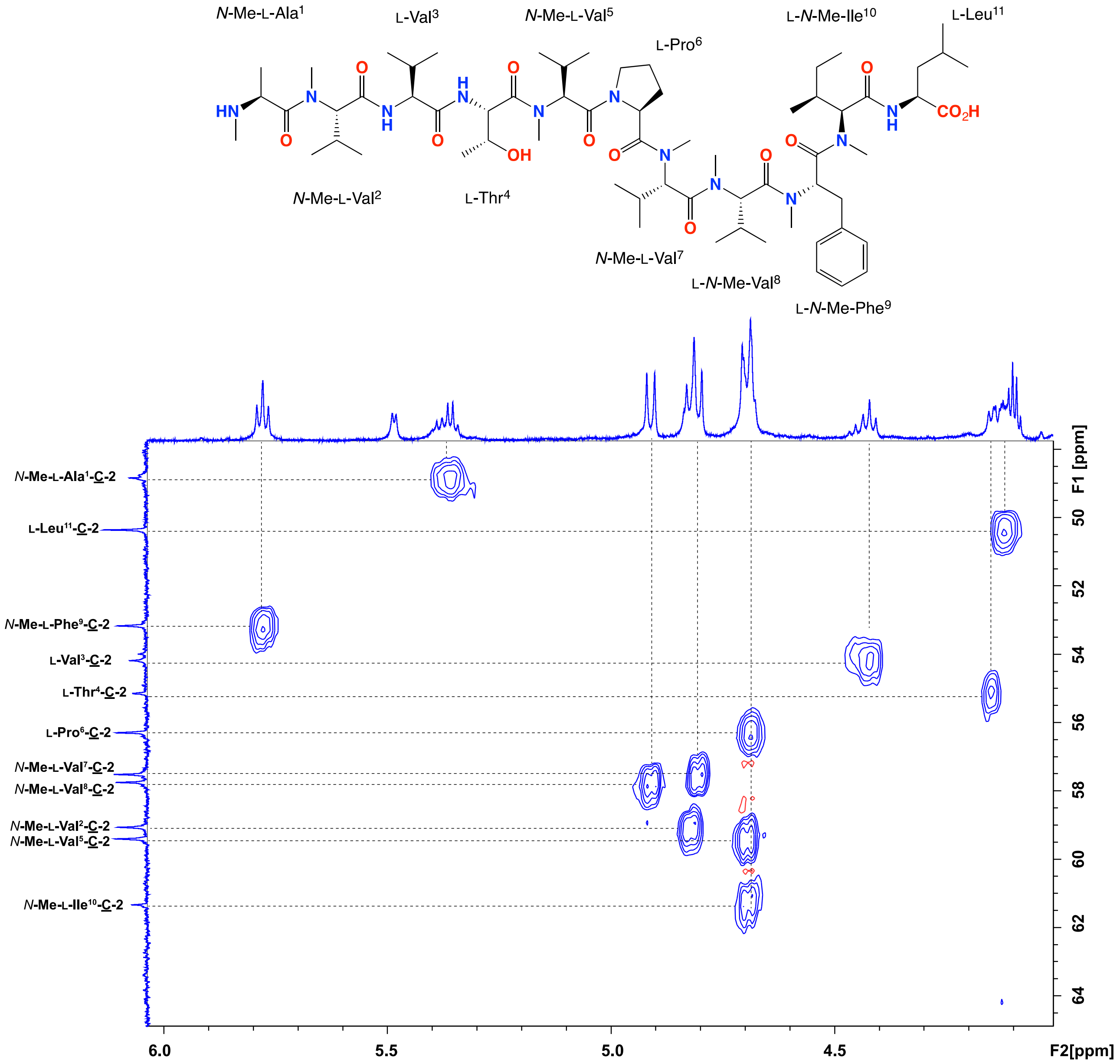

Figure S10: Expanded ^{1}H-^{13}C HSQC (DMSO-d_6) spectrum for talaropeptide A (**2**) showing correlations for 11 amino acid residues.

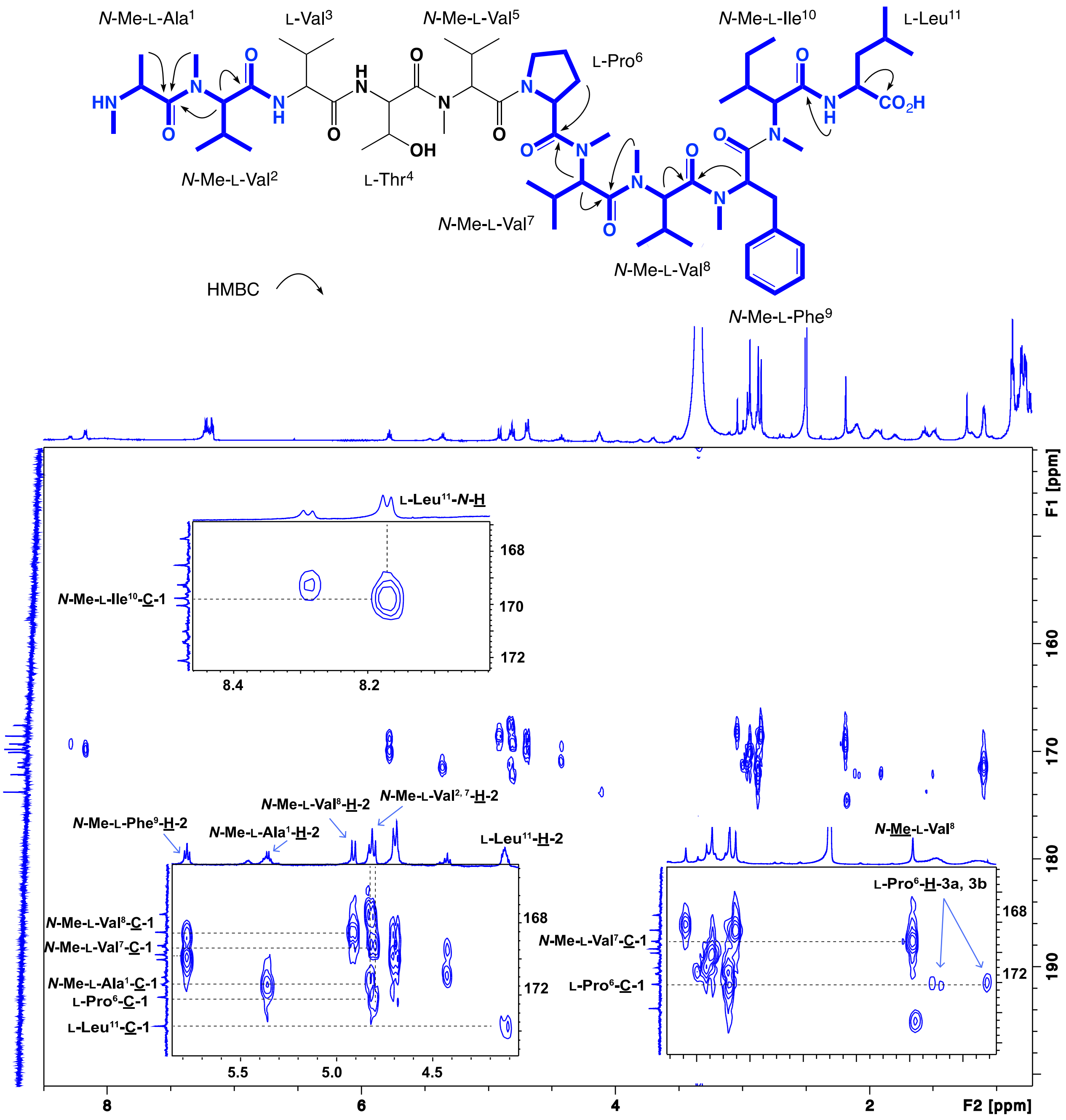

Figure S11: Key 2D NMR correlations (HMBC, DMSO-d_6) of talaropeptide A (**2**). Expansions (inset) show the HMBC correlations for the amino acid residues highlighted in blue.

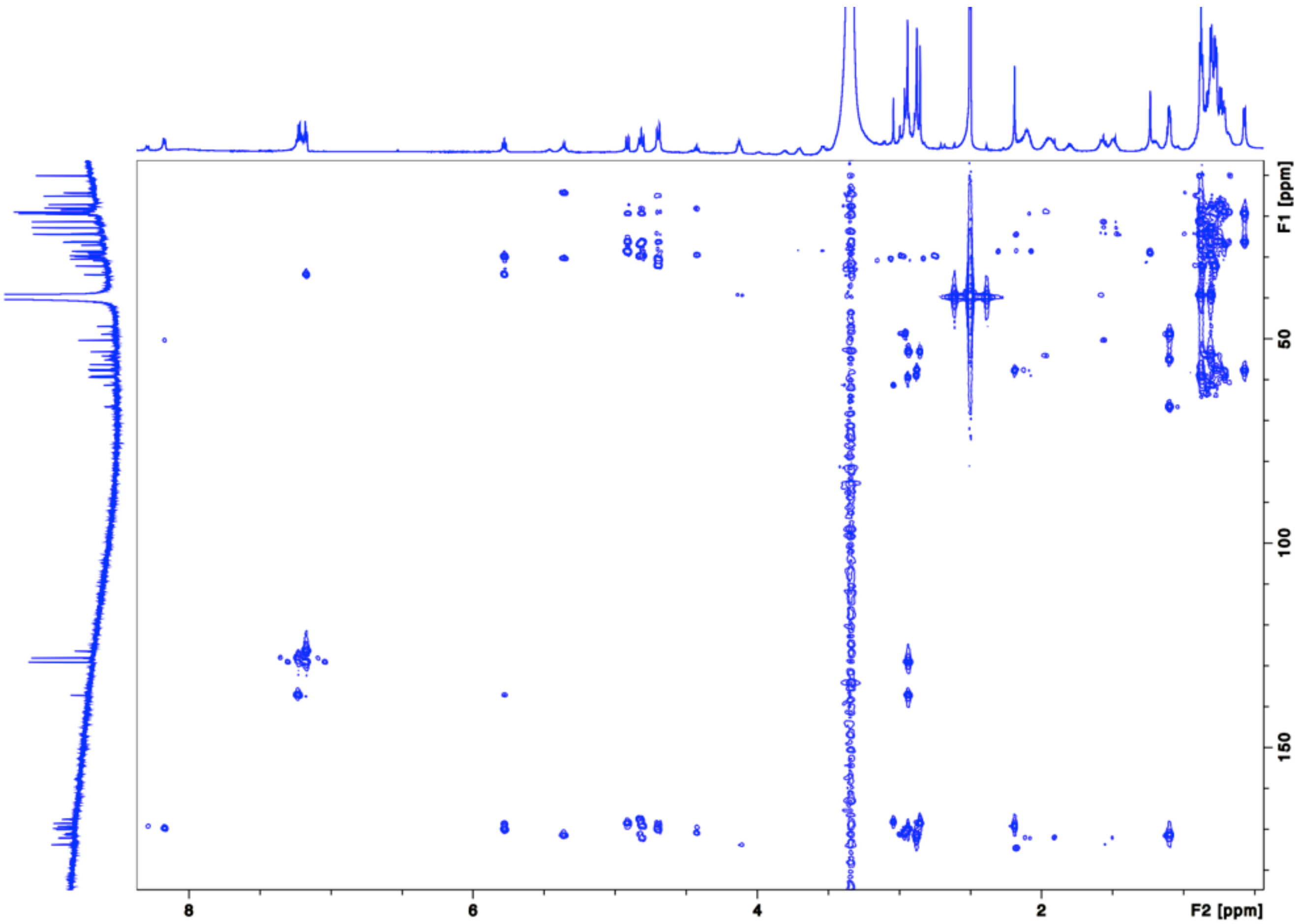

Figure S12: HMBC (DMSO-d_6) full spectrum for talaropeptide A (**2**).

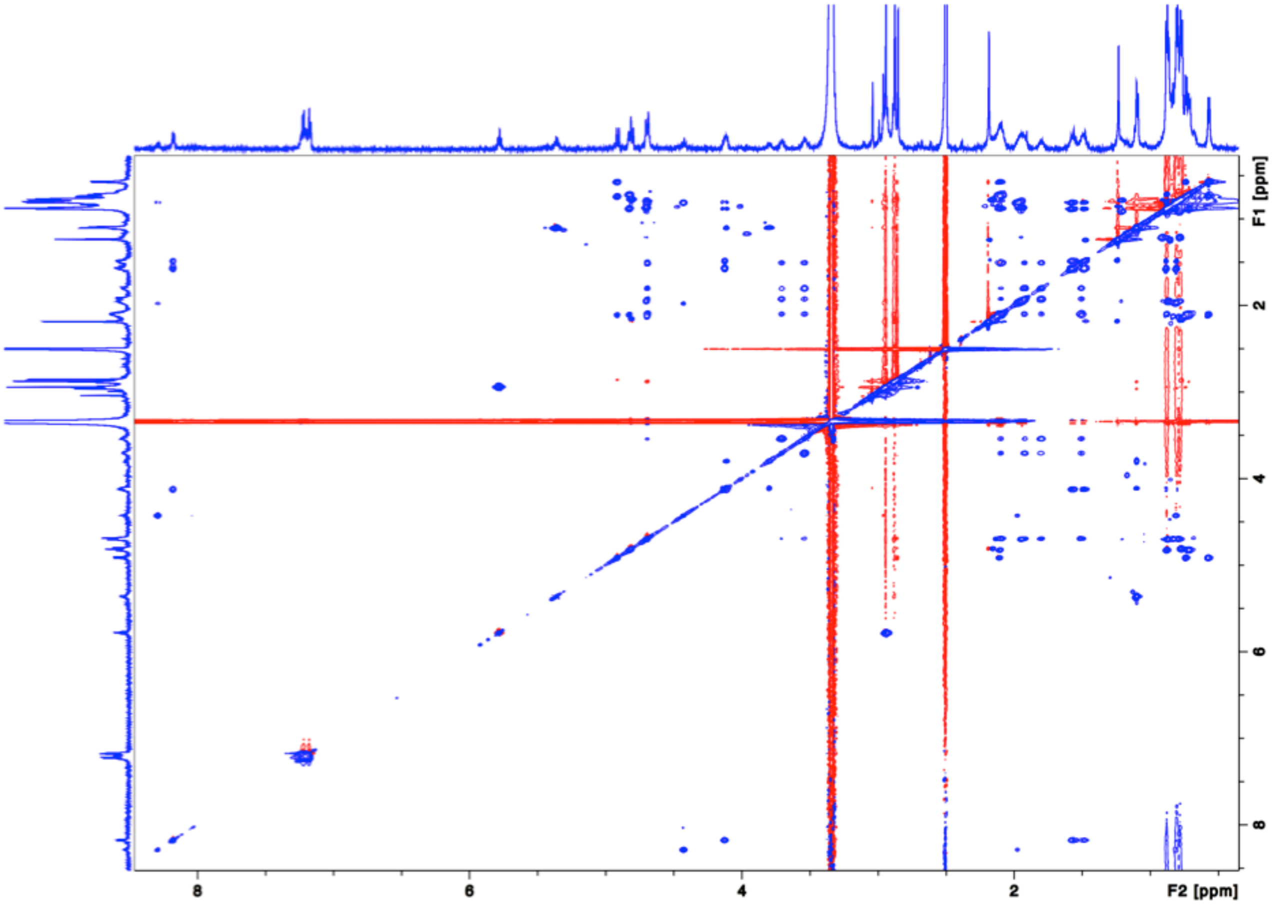

Figure S13: TOCSY data (DMSO-*d*6) for talaropeptide A (**2**).

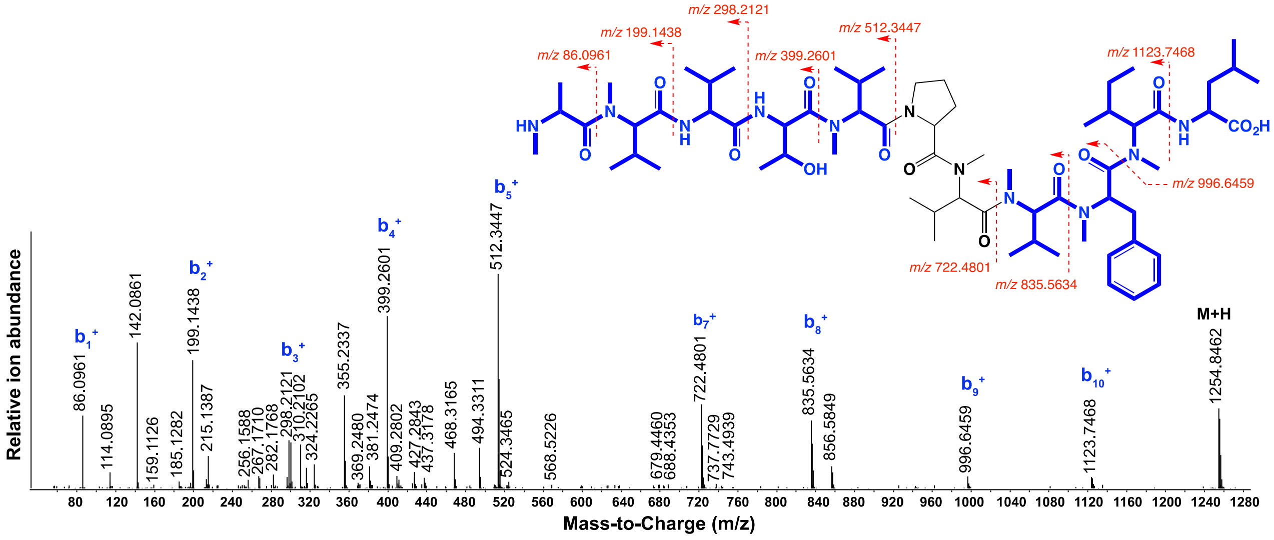

Figure S14: UHPLC-QTOF (MS/MS) fragmentations for talaropeptide A (**2**). The connectivity of amino acid residues confirmed with MS/MS fragmentation is highlighted in blue.

Table S2: NMR (DMSO-d_6) data for talaropeptide A (**2**)

	δ_H, multi, (*J* in Hz)	δ_C	COSY	HMBC	ROESY
***N*-Me-L-Ala[1]**					
1		171.4			
2	5.36, m	48.8	3	1	*N*-Me-L-Val[2]
3	1.09[a]	14.3	2	1, 2	
N-Me	2.96, s	30.3[f]		1, 2	L-Val[3]-H-2
***N*-Me-L-Val[2]**					
1		167.5			
2	4.82, m	59.0	3	1, 3, *N*-Me-L-Ala[1]-C-1	
3	2.10[b]	27.1	2, 4, 5		
4	0.87[c]	18.9[g]		3, 5	
5	0.71, d (*6.4*)	18.1[h]			
N-Me	2.87, s	29.5[i]		2, *N*-Me-L-Ala[1]-C-1	
**L-Val[3]**					
1		171.0			
2	4.42, m	56.2	3, *N*-H	1, 3, 4/5	*N*-Me-L-Ala[1]
3	1.97, m	29.4[i]	2, 4/5		
4, 5	0.80[d]	18.1[h]			
N-H	8.30, d (*8.6*)		2	1	
**L-Thr[4]**					
1		nd			
2	4.15, m	55.1	3		*N*-Me-L-Val[5]
3	3.80, m	66.6	2, 4		
4	1.10[a]	19.3[g]	3	2, 3	
4-OH	5.84, d (*4.6*)		4		
N-H	nd				
***N*-Me-L-Val[5]**					
1		nd			
2	4.68[e]	61.3			
3	2.10[b]	26.3[j]			
4	0.87[c]	18.9[g]			
5	0.82[d]	19.2[g]			
N-Me	3.04, s	30.4[f]		2	L-Thr[4]-H-2
**L-Pro[6]**					
1		172.1			
2	4.69[e]	56.3	3a, 3b	4	*N*-Me-L-Val[7]
3	a 2.09[b]	28.5[k]	2, 4a, 4b, 3b	1, 5	
	b 1.50, m		2, 4a, 4b, 3a	1, 5	
4	a 1.92, m	24.4[l]	3a, 3b, 5a, 5b	2, 3	
	b 1.79, m		3a, 3b, 5a, 5b	2, 3	
5	a 3.70, m	47.0	4a, 4b	3, 4	
	b 3.53, m		4a, 4b	3, 4	

***N*-Me-L-Val^{7}**					
1		169.3			
2	4.80 m	57.5	3	1, 4/5, L-Pro^{6}-C-1	*N*-Me-L-Val^{8}
3	2.15, m	26.2[j]	2		
4, 5	0.76, m	18.0[h]	3	2	
N-Me	2.87, s	29.7[i]		2, L-Pro^{6} -C-1	L-Pro^{6}-H-2
***N*-Me-L-Val^{8}**					
1		168.5			
2	4.91, d (*10.5*)	57.7	3	1, 4, 5, *N*-Me	*N*-Me-L-Phe^{9}
3	2.10[b]	26.3[j]	2	4, 5	
4	0.73, d (*6.5*)	19.1[g]	3		
5	0.56, d (*6.5*)	17.1		2, 3, 5	
N-Me	2.18, s	28.6[k]		2, *N*-Me-L-Val^{7}-C-1	2, *N*-Me-L-Val^{7}
***N*-Me-L-Phe^{9}**					
1		170.0			
2	5.77, t (*7.6*)	53.1	3	1, 3, *N*-Me, *N*-Me-L-Val^{8}-C-1	5/9
3	2.94, m	34.3	2	1, 4, 5	
4		137.3			
5/9	7.71, m	129.1	6, 8	5, 7	
6/8	7.22, m	128.0	5, 9	4, 8	
7	7.17, m	126.3			
N-Me	2.85, s	29.9[i]		1, 2, *N*-Me-Val^{8}-C-1	2, *N*-Me-L-Val^{8}
***N*-Me-L-Ile^{10}**					
1		169.7			
2	4.69[e]	59.4	3	3, 4	
3	1.94, m	32.1	4, 6		
4	a 1.20, m	24.3[l]	3, 5		
	b 0.90, m				
5	0.78, m	10.0	4	2, 3	
6	0.86[c]	15.1	3	2, 3	
N-Me	2.94, s	30.4[f]		2	L-Leu^{11}-H-2
**L-Leu^{11}**					
1		173.7			
2	4.11, m	50.3	3	1, 3, 4, 6, 5	
3	a 1.56, m	39.2	2, 3b, 4	1, 2, 4, 6, 5	
	b 1.48, m		2, 3a, 4	1	
4	1.57, m	24.2[l]	5, 6		
5	0.88[c]	22.8	4		
6	0.80[d]	21.3	4		
N-H	8.17, d (*7.4*)		2	2, 3, *N*-Me-L-Ile^{10}-C-1	
OH	nd				

[a-e]Assignments with the same superscript are overlapping. [f-l] Assignments with the same superscript may be interchanged. [nd] resonances not detected.

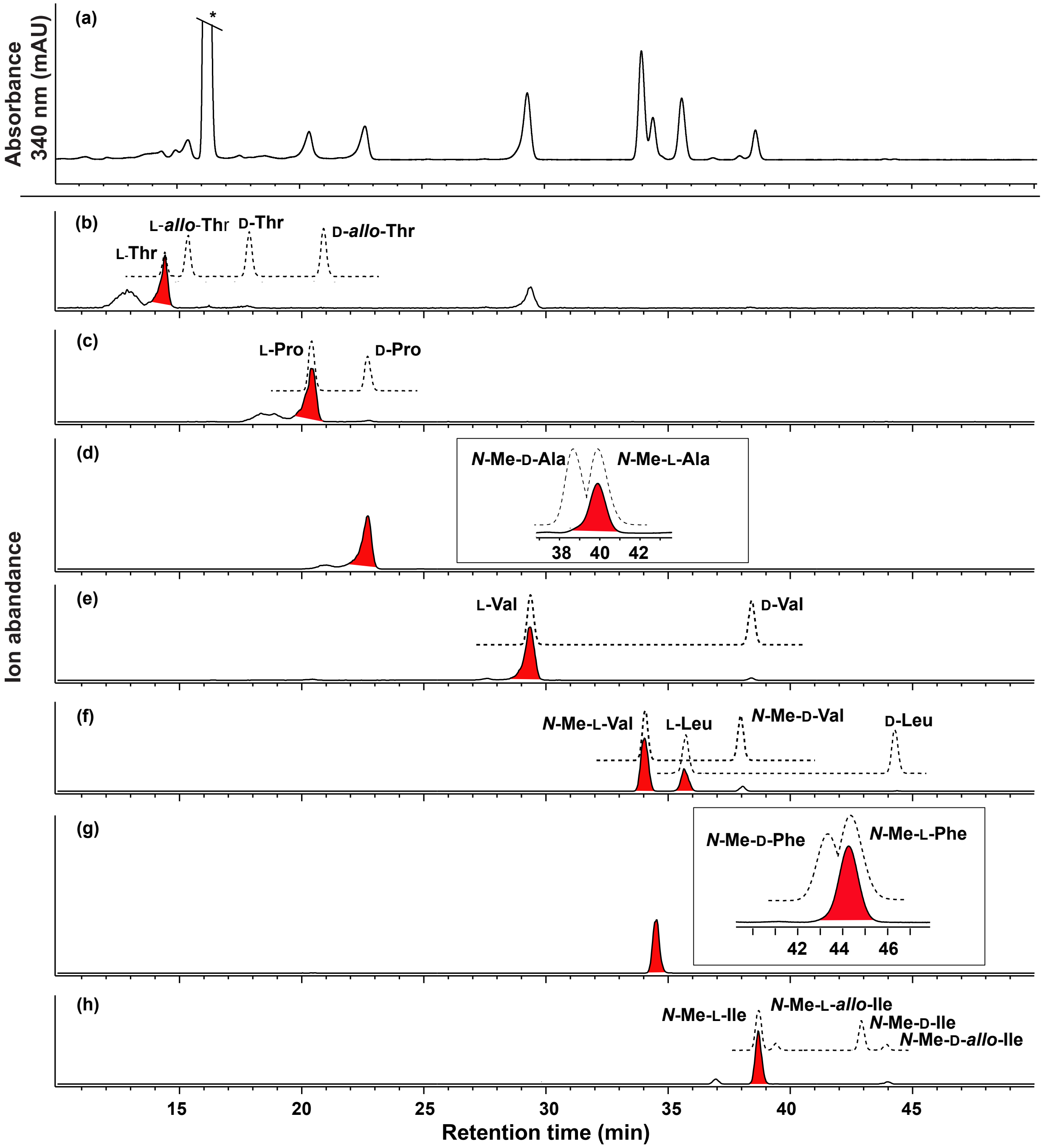

Figure S15: C3 Marfey's analysis for talaropeptide A (**2**). Trace (a) is the C3 HPLC-DAD (340 nm) chromatogram that exhibits L-FDAA amino acid derivatives. Traces (b-h) are C3 HPLC-MS-SIE chromatograms for L-FDAA derivatives of amino acid standards (broken lines) and the acid hydrolysate of **2** (shaded peaks). The inset in (d and g) shows the C18 HPLC-DAD (340 nm) chromatogram. Traces confirm that **2** incorporates (b) L-Thr (SIE *m/z* 372), (c) L-Pro (SIE *m/z* 368), (d) *N*-Me- L-Ala (SIE *m/z* 356) (e) L-Val (SIE *m/z* 370) (f) *N*-Me- L-Val and L-Leu (SIE *m/z* 384), (g) *N*-Me- L-Phe (SIE *m/z* 432) and (h) *N*-Me- L-Ile (SIE *m/z* 398). *Residual Marfey's reagent.

4 Talaropeptide B (3)

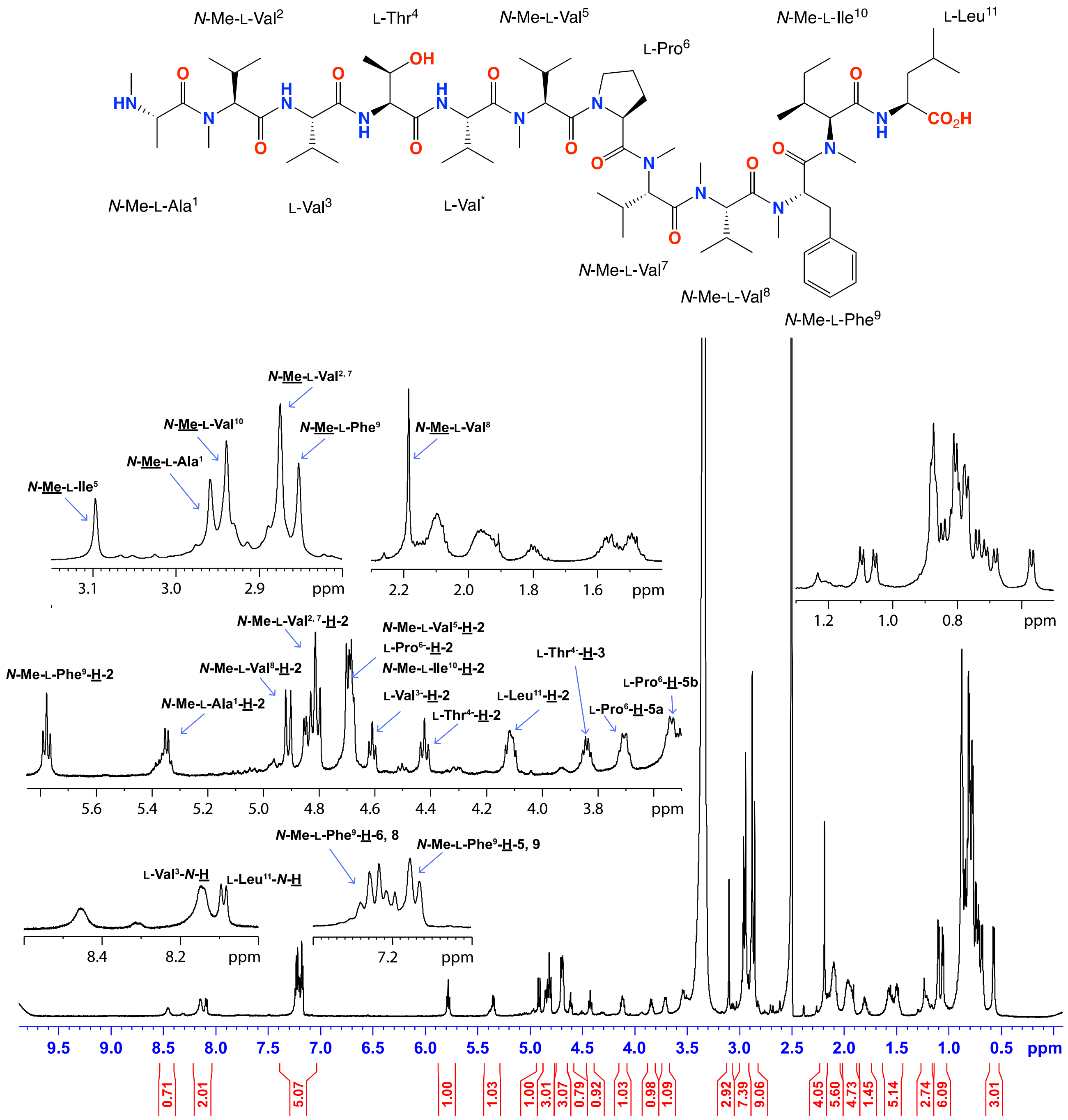

Figure S16: ^{1}H NMR (600 MHz, DMSO-d_6) spectrum of talaropeptide B (**3**).

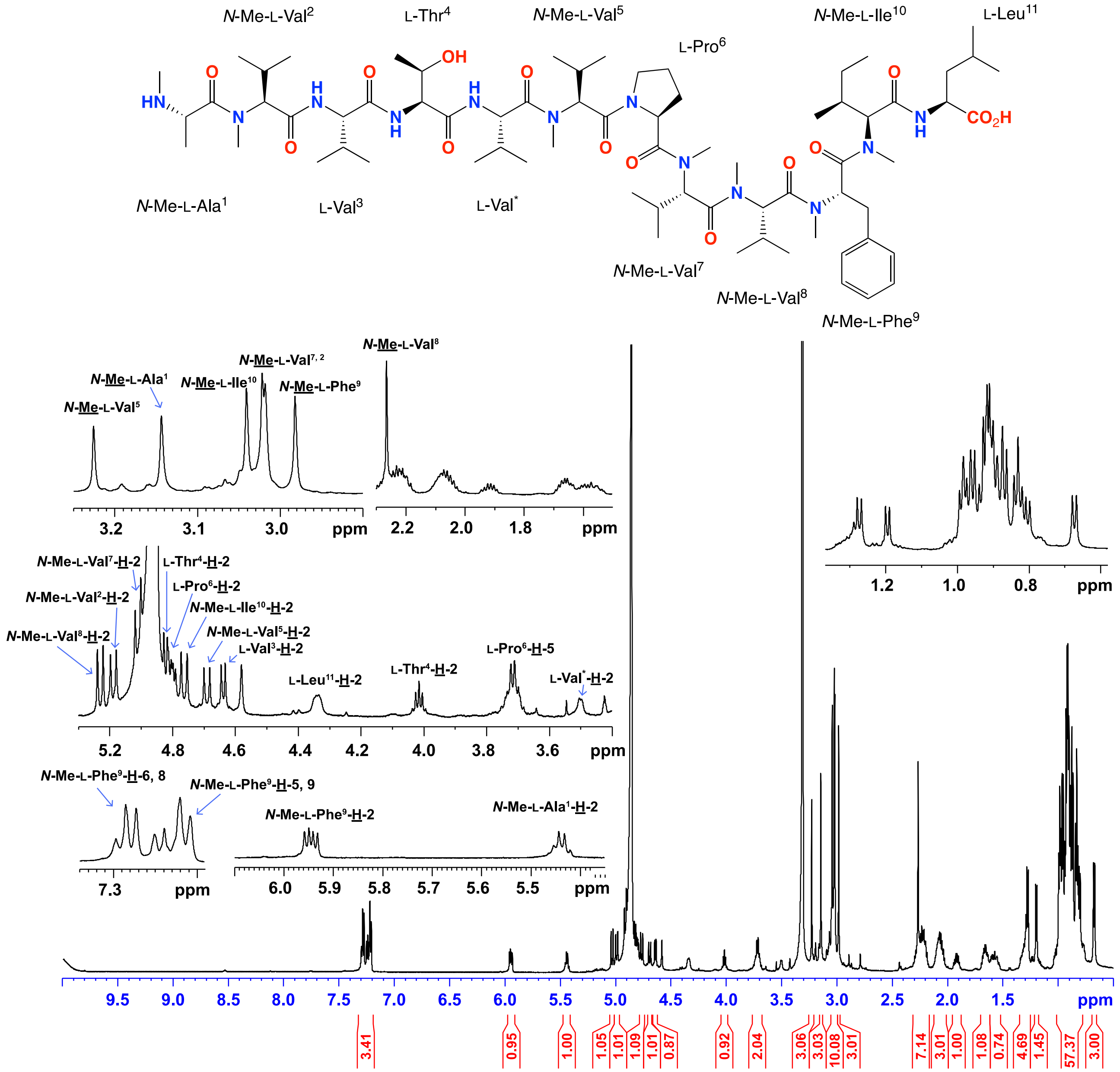

Figure S17: ^{1}H NMR (600 MHz, methanol-d_4) spectrum of talaropeptide B (**3**).

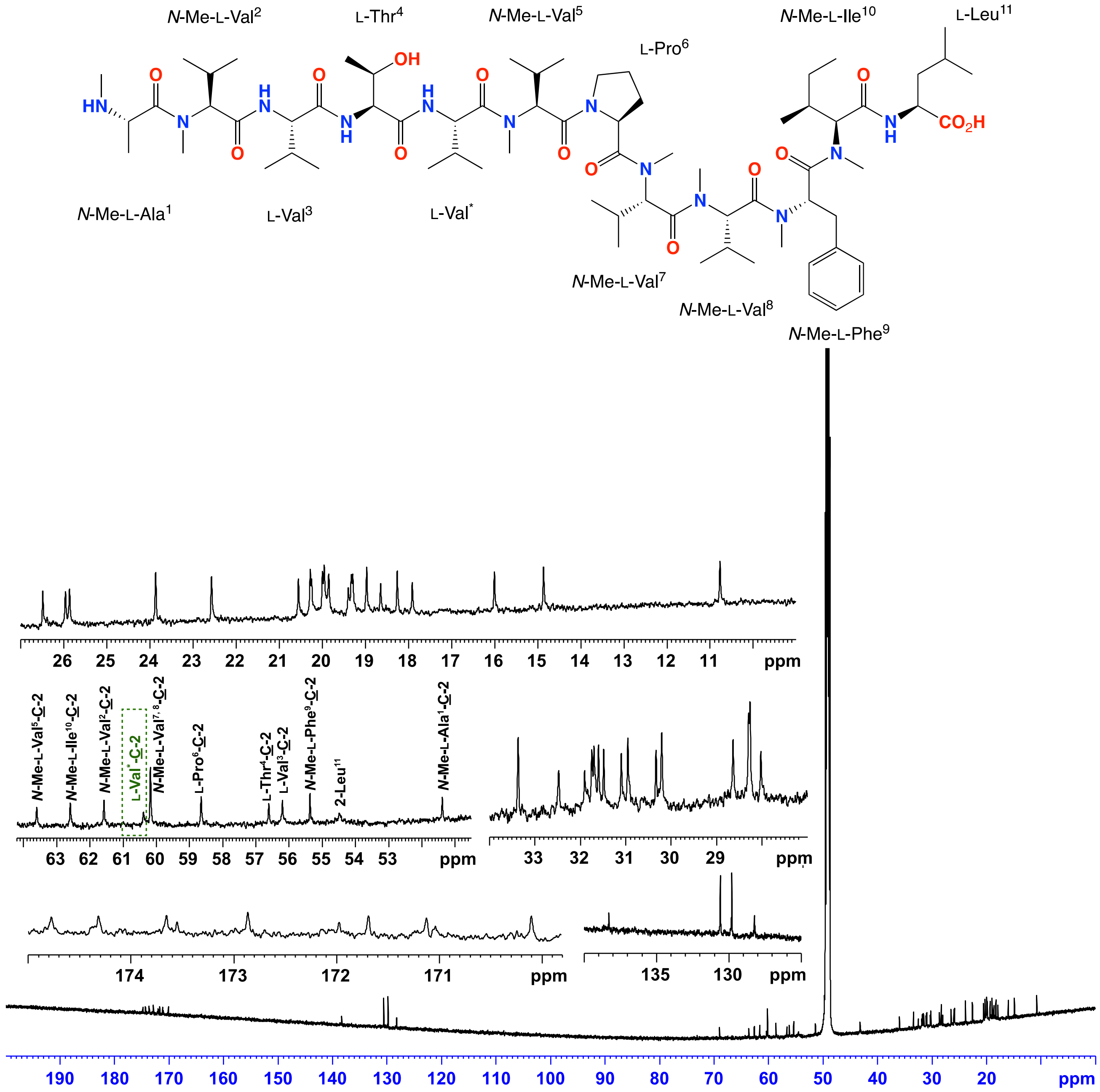

Figure S18: ^{13}C NMR (150 MHz, methanol-d_4) spectrum of talaropeptide B (**3**)

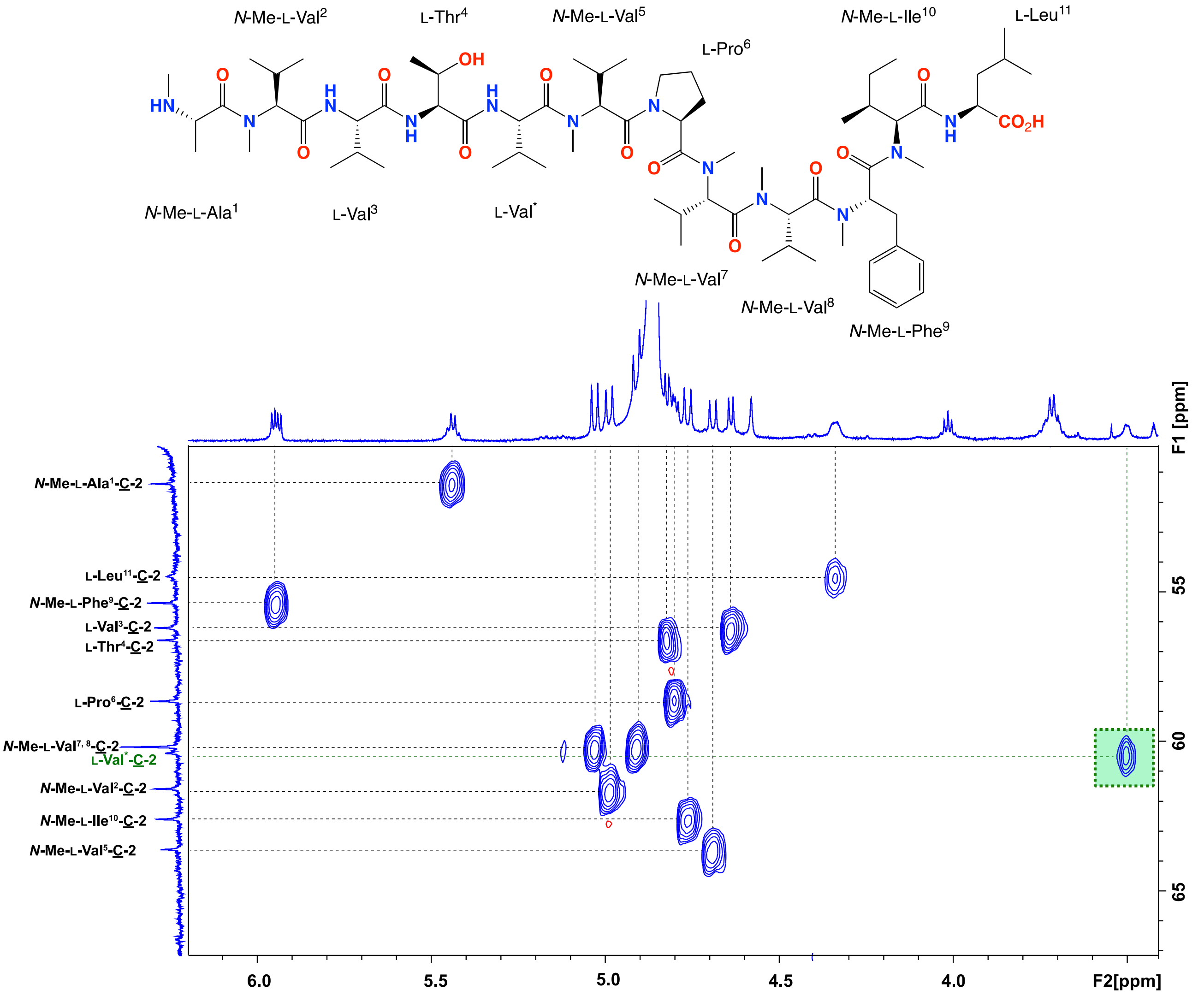

Figure S19: ^1H-^{13}C HSQC (methanol-d_4) spectrum for talaropeptide B (**3**), which shows resonances for the L-Val* residue (highlighted in green)

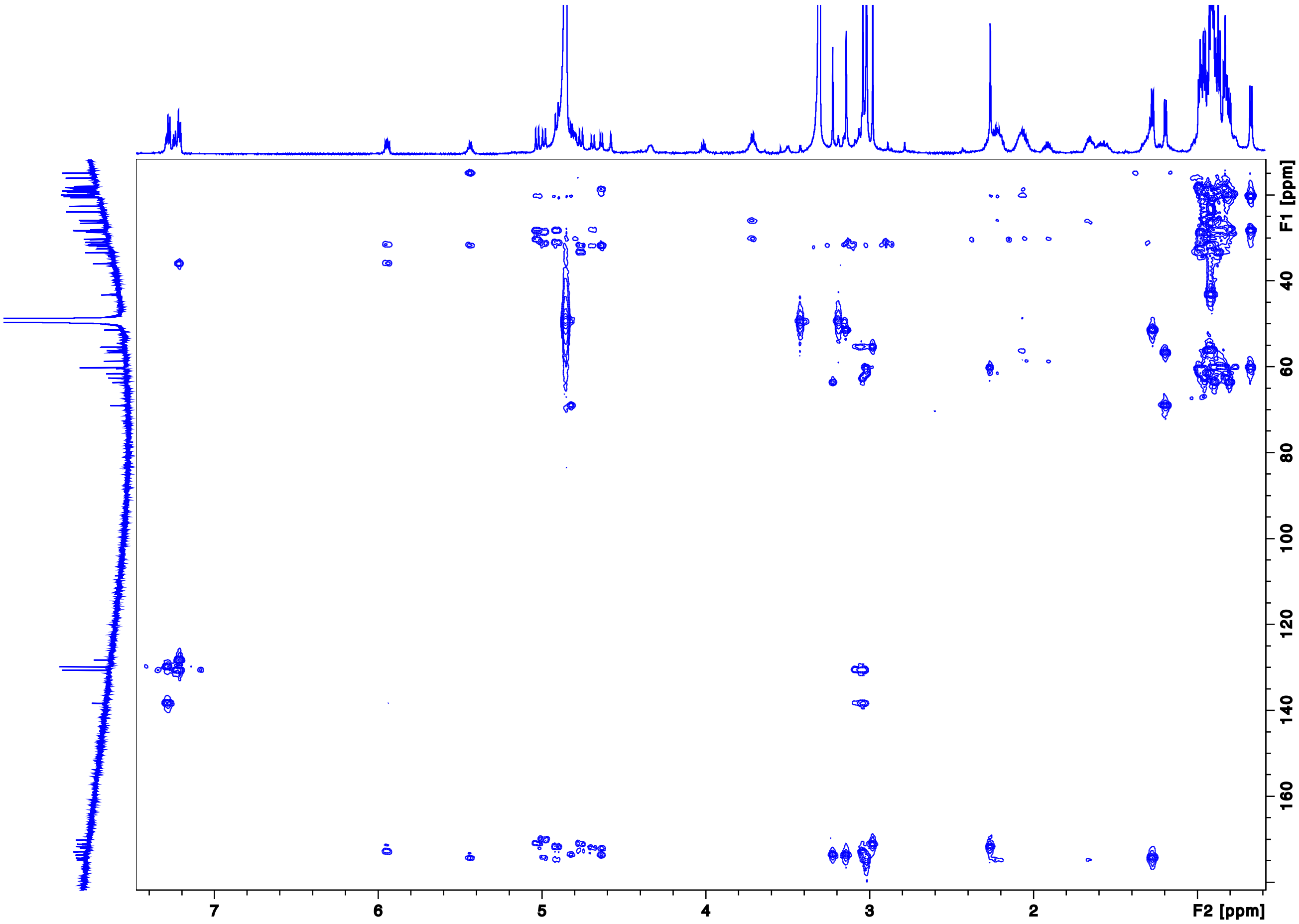

Figure S20: HMBC (methanol-d_4) spectrum for talaropeptide B (**3**).

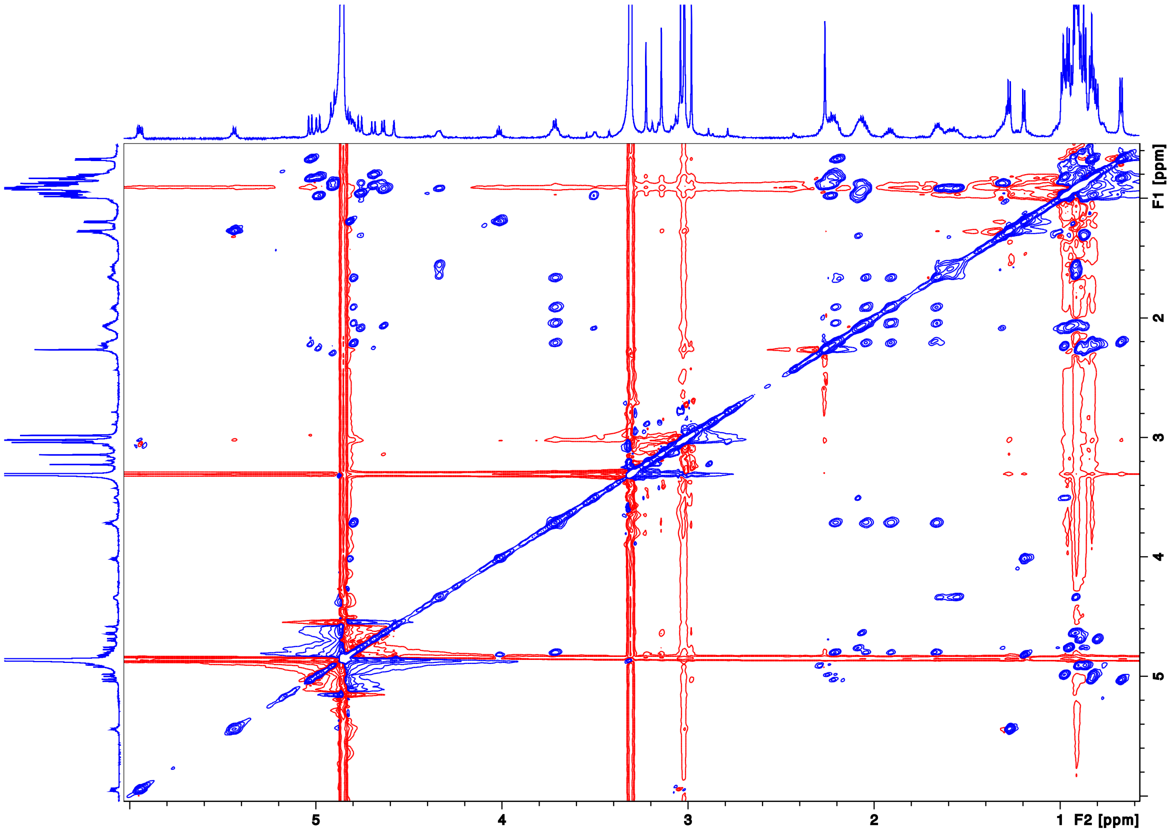

Figure S 21: TOCSY (methanol-d_4) spectrum for talaropeptide B (**3**).

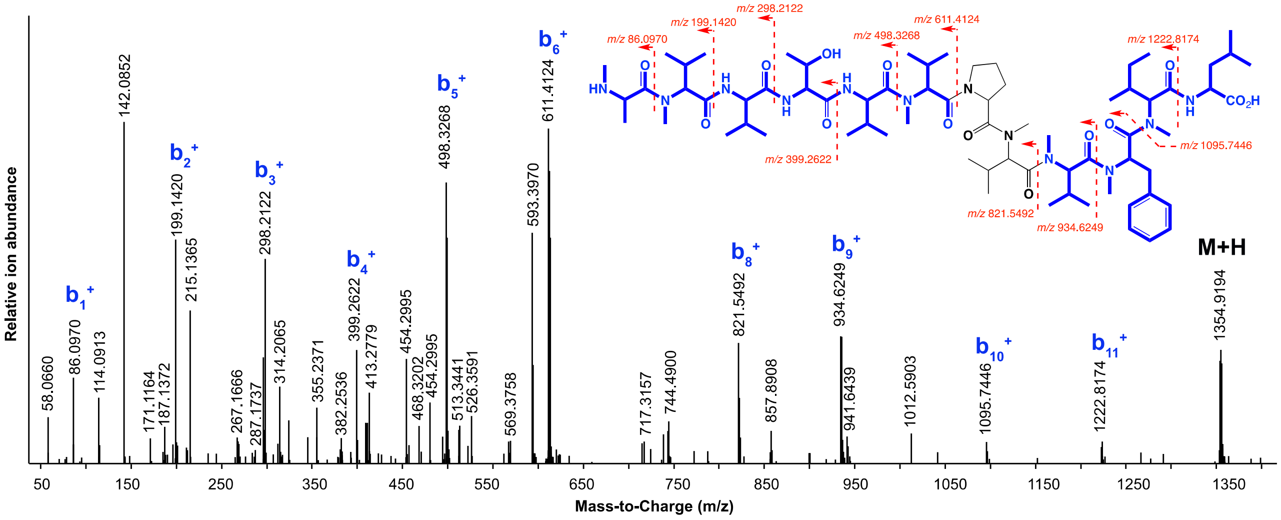

Figure S22: UHPLC-QTOF (MS/MS) fragmentation for talaropeptide B (**3**). The connectivity of amino acid residues confirmed with MS/MS fragmentation is highlighted in blue.

Table S3: NMR (methanol-d_4) data of talaropeptide B (**3**)

	δ_H, mult, (*J* in Hz)	δ_C	COSY	HMBC	ROESY
***N*-Me-L-Ala^1**					
1		174.3			
2	5.43, m	51.4	3	1, 3, *N*-Me	*N*-Me-L-Val^2
3	1.27, d (*6.9*)	14.8	2	2	
N-Me	3.14, s	31.7[j]		2	L-Val^3-H-2
***N*-Me-L-Val^2**					
1		170.1			
2	4.98, d (*10.8*)	61.6	3	1, 3, 4, 5, *N*-Me-L-Ala^1-C-1	
3	2.24[a]	28.6	2, 4		
4	0.97[b]	19.8[k]	3, 5	2, 3, 5	
5	0.82, d (*6.6*)	19.4[l]	4	2	
N-Me	3.01, s	31.1[m]		2, *N*-Me-L-Ala^1-C-1	*N*-Me-L-Ala^1-H-2
**L-Val^3**					
1		173.6[n]			
2	4.63, d (*7.6*)	56.2	3	1	*N*-Me-L-Ala^1
3	2.05[c]	31.7[j]	2, 4/5		
4	0.92[d]	18.6[o]	3	2	
5	0.93[e]	18.9[o]	3		
**L-Thr^4**					
1		173.6[n]			
2	4.82[f]	56.6	3	1, 3, 4	
3	4.01, m	68.9	2, 4		
4	1.19, d (*6.3*)	20.3[k]	3	2, 3	
**L-Val***					
1		nd			
2	3.50, br s	60.3	3, 4/5		
3	2.08, m	32.4	2, 4/5		
4	0.93[e]	19.3[l]			
5	0.96[b]	17.9	2		
***N*-Me-L-Val^5**					
1		171.9			
2	4.69, d (*11.0*)	63.6	3	1, 3, *N*-Me	
3	2.23[a]	28.0	2, 5	2	
4	0.89[g]	19.9[k]			
5	0.80, d (*6.5*)	19.2[l]	3	2, 4	
N-Me	3.22, s	31.9[j]		2	
**L-Pro^6**					
1		174.7			
2	4.80[f]	58.6	3a, 3b	4	*N*-Me-L-Val^7
3	a 2.21[h]	30.2[p]	2, 3b, 4a, 4b	1, 2	
	b 1.66, m		2, 3a, 4a, 4b	1, 2, 4	
4	a 2.04[c]	25.8[q]	3a, 3b, 4b, 5	2	

	b 1.91, m		3a, 3b, 4a, 5	2, 3	
5	3.71, m	49.0	4a, 4b	3, 4	
***N*-Me-L-Val^{7}**					
1		171.6			
2	4.91, d (*10.6*)	60.2	3	1, 3, *N*-Me, L-Pro^{6}-C-1	
3	2.27.m	28.2	2, 4, 5		
4	0.89[g]	20.5	3		
5	0.86[i]	18.6[o]	3		
N-Me	3.02, s	31.1[m]		2, L-Pro^{6}-C-1	L-Pro^{6}-H-2
***N*-Me-L-Val^{8}**					
1		171.0[r]			
2	5.03, d (*10.5*)	60.1	3	1, 3	5, *N*-Me-L-Phe^{9}
3	2.22[h]	28.4	2, 4, 5		
4	0.83, d (*6.7*)	20.2[k]	3		
5	0.67, d (*6.7*)	18.2	3	3, 4	
N-Me	2.26, s	30.3[p]		2, *N*-Me-L-Val^{7}-C-1	*N*-Me-L-Val^{7}-H-2
***N*-Me-L-Phe^{9}**					
1		172.8			
2	5.94, dd (*10.3, 5.4*)	55.4	3	1, 3, *N*-Me, *N*-Me-Val^{8}-C-1	*N*-Me-L-Ile^{10}
3	3.04, m	35.9	2	4, 5/9	
4		138.3	3		
5/9	7.21, m	130.5	6	7, 9	
6/8	7.28, m	129.7	7, 5	4, 8	
7	7.24, m	128.2	6, 8	5, 9	
N-Me	2.98, s	31.5[j]		2, *N*-Me-L-Val^{8}-C-1	*N*-Me-L-Val^{8}-H-2
***N*-Me-L-Ile^{10}**					
1		171.1[r]			
2	4.76, d (*11.4*)	62.6	3	3, *N*-Me	
3	2.09, m	33.4	2, 4, 6		
4	a 1.31, m	25.9[q]	3, 5		
	b 1.02, m		3		
5	0.87[i]	10.7	4a, 4b	3	
6	0.94 d (*6.6*)	16.0	3	2, 3	
N-Me	3.04, s	31.6[j]		2, *N*-Me-L-Phe^{9}-C-1	*N*-Me-L-Phe^{9}-H-2
**L-Leu^{11}**					
1		[nd]			
2	4.34, br s	54.5	3a, 3b		
3	a 1.64, m	43.1	2, 3		
	b 1.55, m				
4	1.59, m	26.5	3b, 5, 6		
5	0.92[d]	23.8	4		
6	0.91[d]	22.6			

[a-i]Assignments with the same superscript are overlapping. [j-r] Assignments with the same superscript may be interchanged.
[nd] resonances not detected

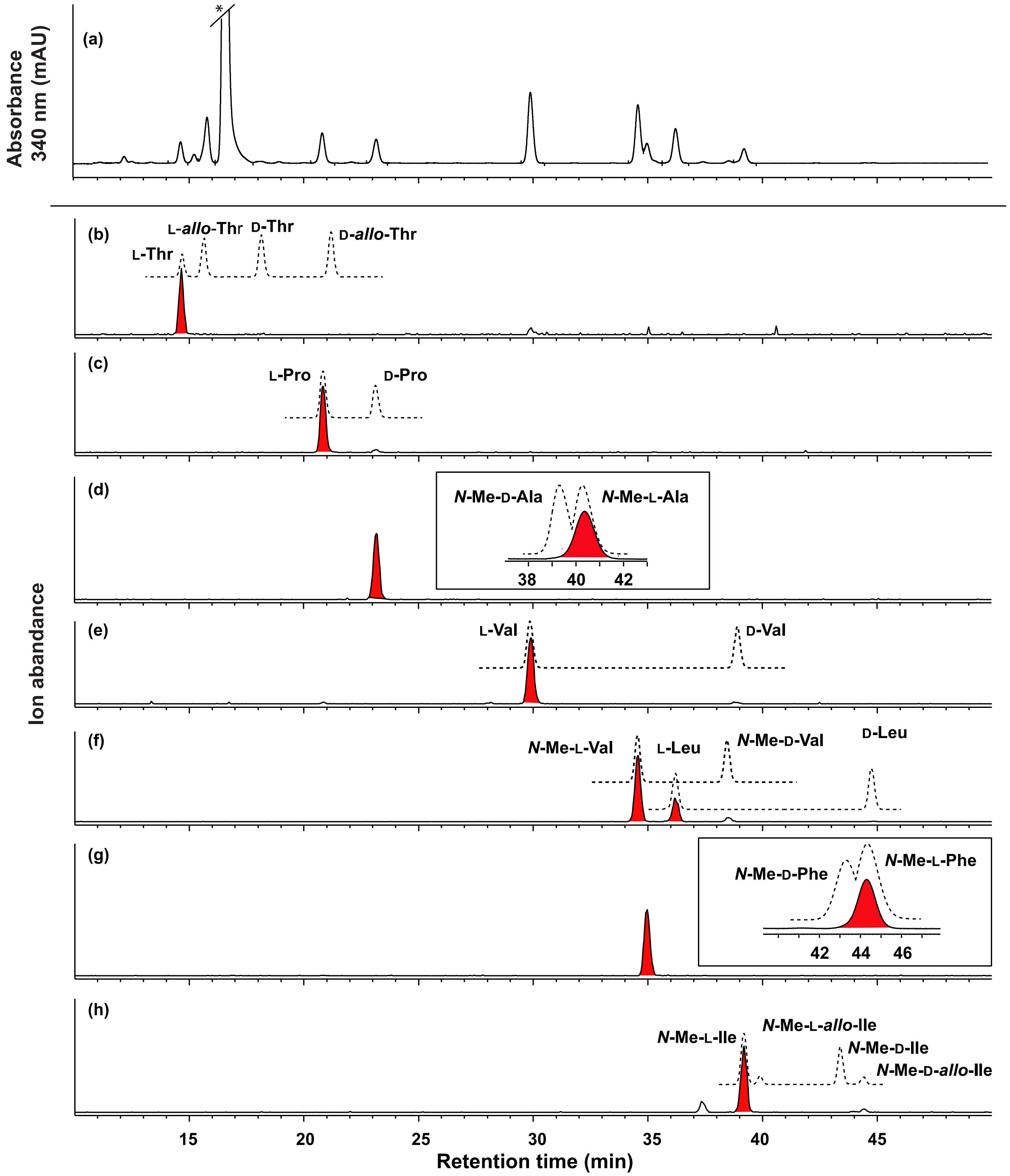

Figure S23: C_3 Marfey's analysis for talaropeptide B (**3**). Trace (a) is the C_3 HPLC-DAD (340 nm) chromatogram that exhibits L-FDAA amino acid derivatives. Traces (b-h) are C_3 HPLC-MS-SIE chromatograms for L-FDAA derivatives of amino acid standards (broken lines) and the acid hydrolysate of **3** (shaded peaks). The inset in (d and g) shows the C_{18} HPLC-DAD (340 nm) chromatogram. Traces confirm that **3** incorporates (b) L-Thr (SIE *m/z* 372), (c) L-Pro (SIE *m/z* 368), (d) *N*-Me-L-Ala (SIE *m/z* 356) (e) L-Val (SIE *m/z* 370) (f) *N*-Me-L-Val and L-Leu (SIE *m/z* 384), (g) *N*-Me-L-Phe (SIE *m/z* 432) and (h) *N*-Me-L-Ile (SIE *m/z* 398). * residual Marfey's reagents

5 Talaropeptide C (4)

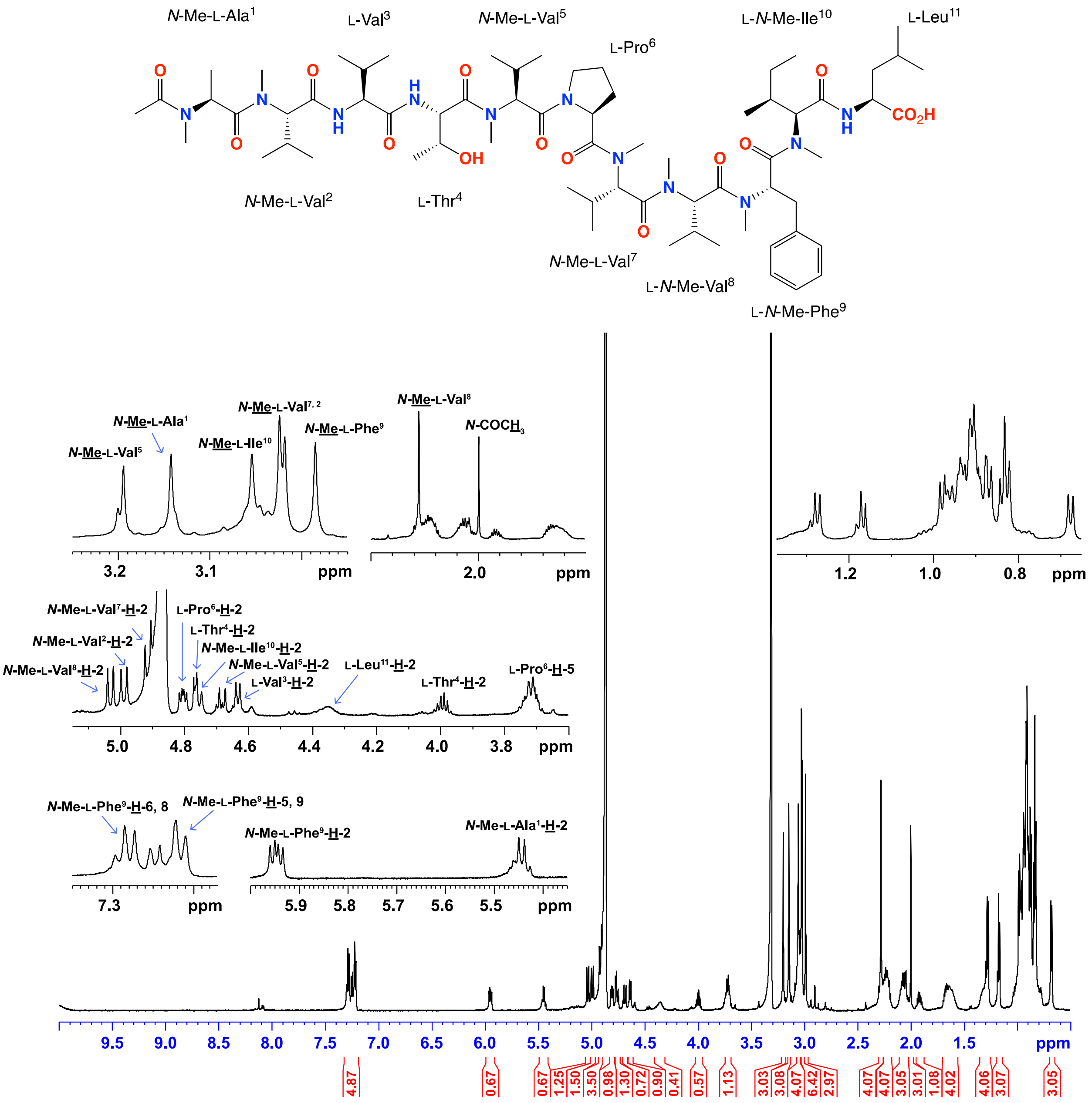

Figure S24: ^{1}H NMR (methanol-d_4) spectrum for talaropeptide C (**4**).

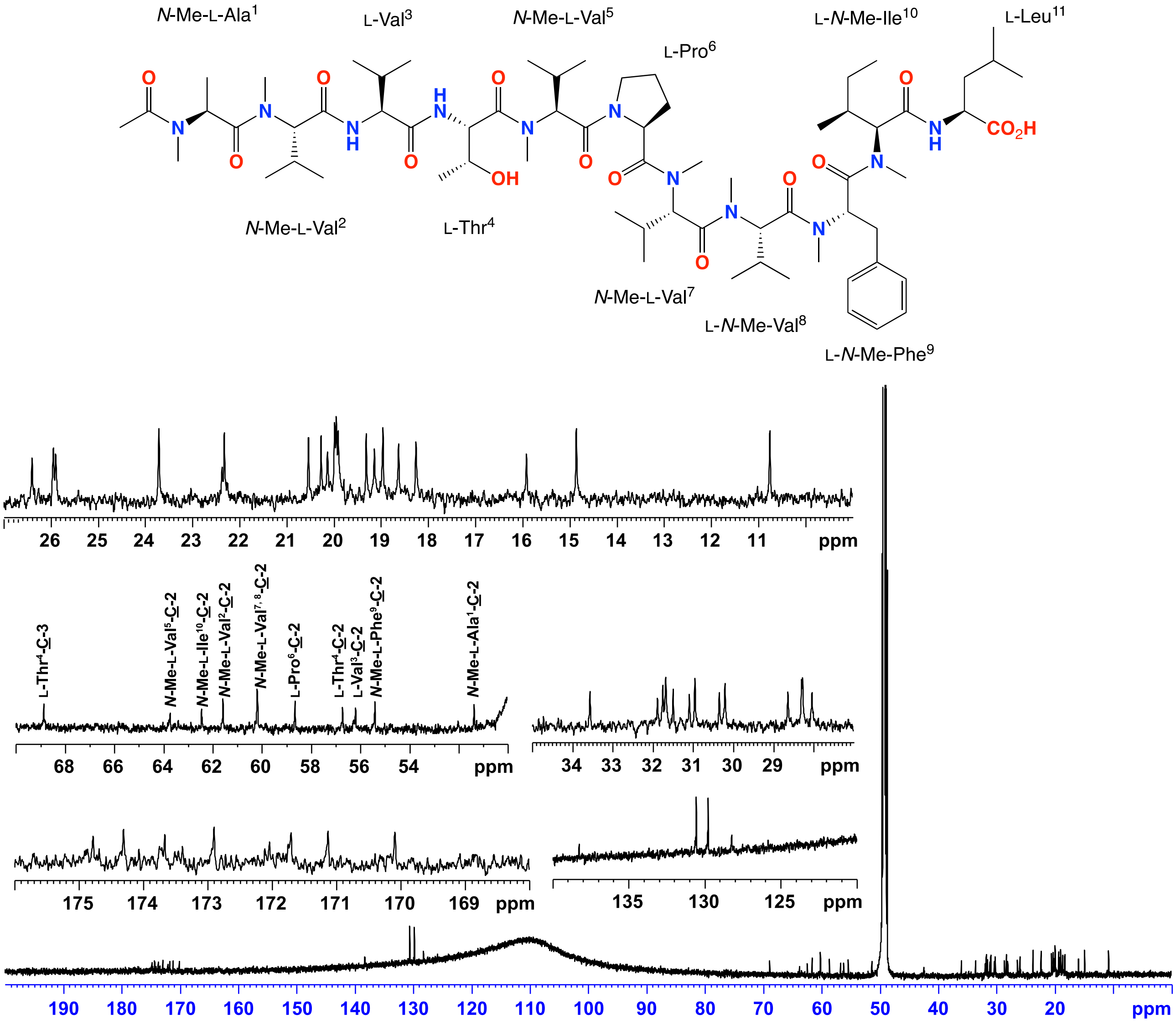

Figure S25: ^{13}C NMR (150 MHz, methanol-d_4) spectrum for talaropeptide C (**4**).

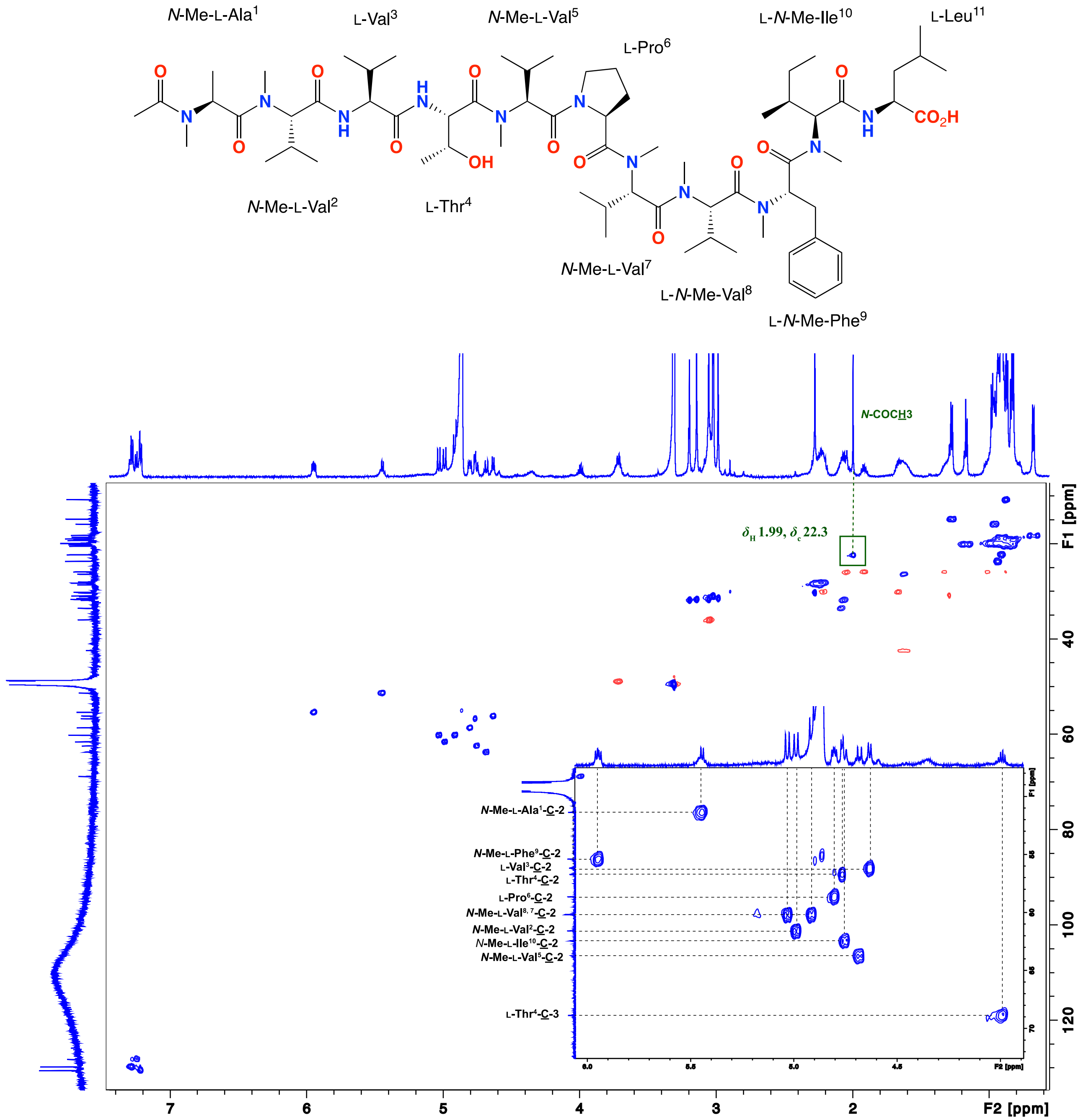

Figure S 26: ^{1}H-^{13}C HSQC (methanol-d_4) spectrum for talaropeptide C (**4**). Expansion shows resonances for 11 amino acid α-protons. *N*-CO<u>CH</u>3 of *N*-Me-Ala[1] is highlighted in green.

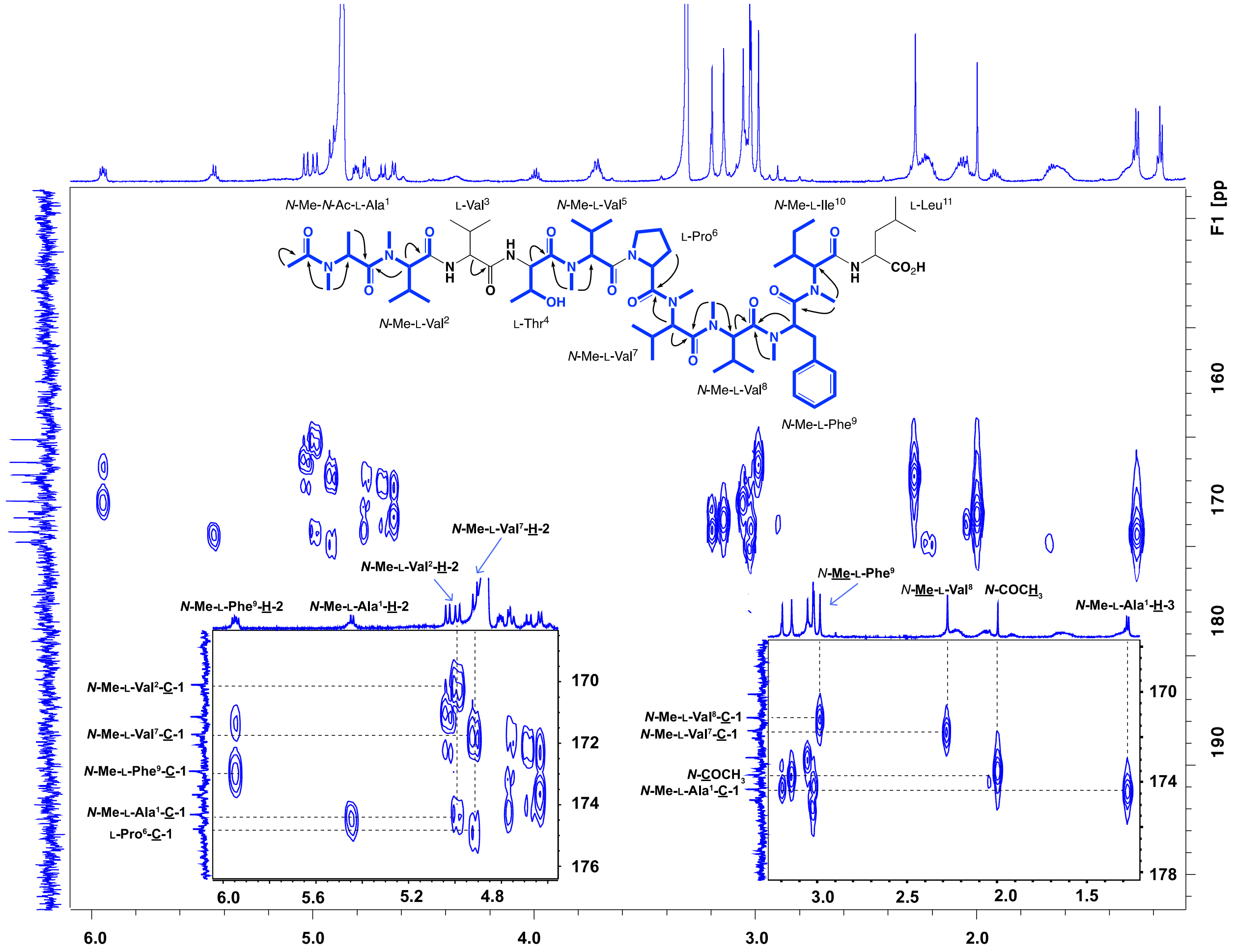

Figure S27: Expanded HMBC (methanol-d_4) spectrum that shows key HBMC correlations for talaropeptide C (**4**).

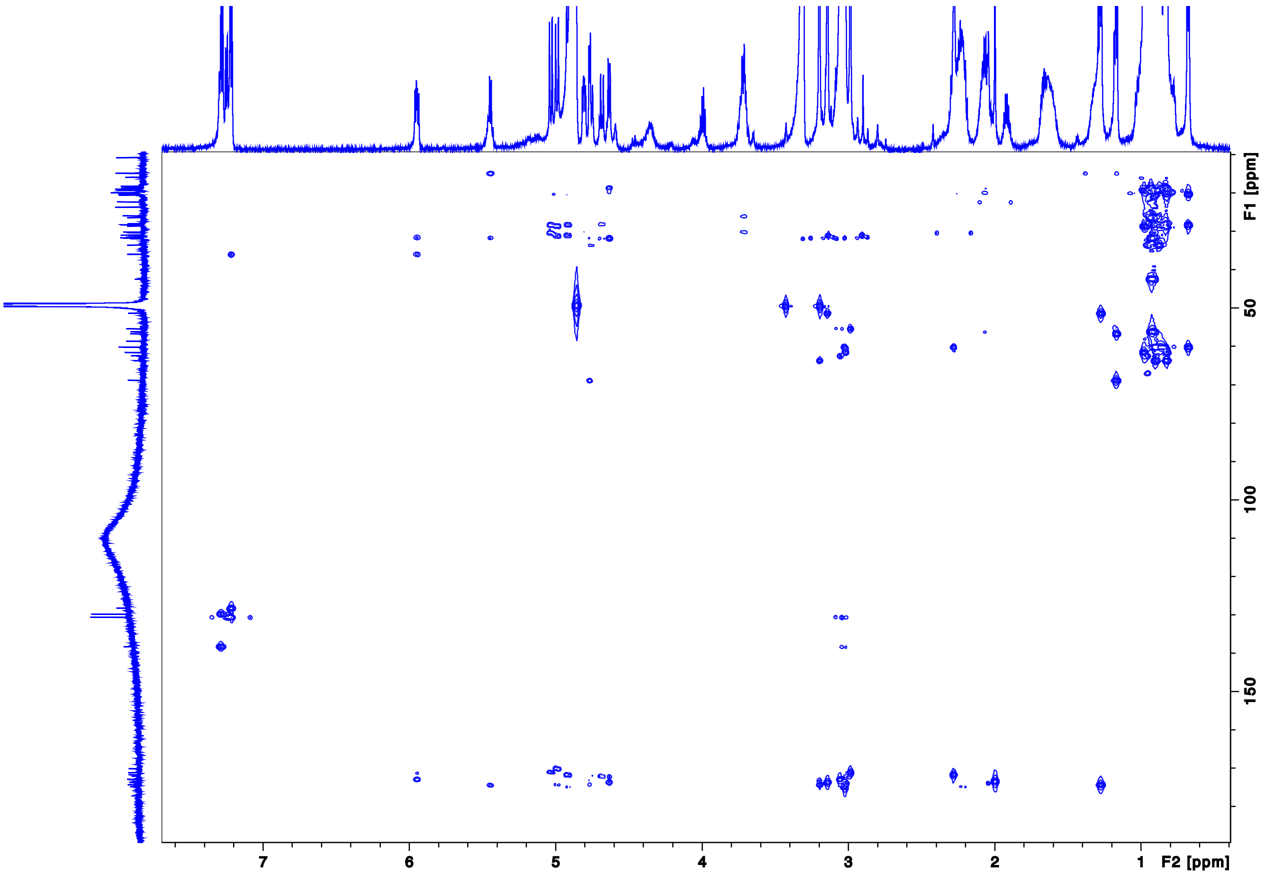

Figure S28: Full HMBC (methanol-*d*4) spectrum of talaropeptide C (**4**).

Supplementary Material

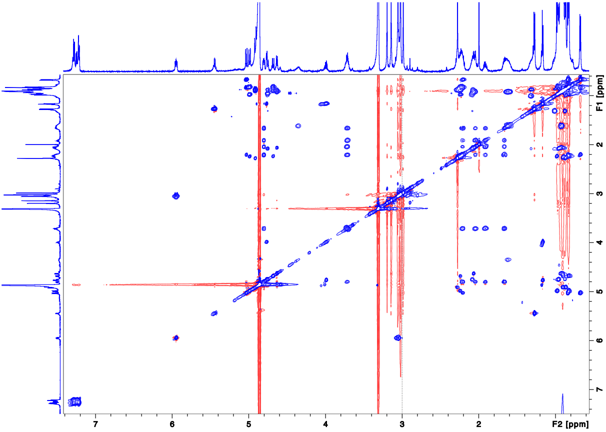

Figure S29: TOCSY (methanol-d_4) spectrum for talaropeptide C (**4**).

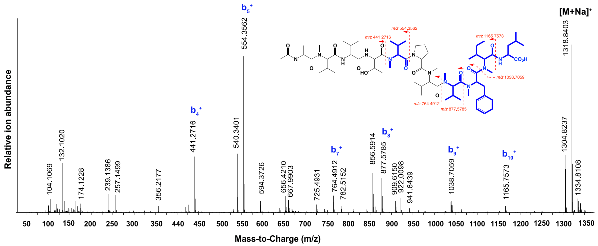

Figure S30: UHPLC-QTOF (MS/MS) fragmentation for talaropeptide C (**4**). The connectivity of amino acid residues confirmed with MS/MS fragmentation is highlighted in blue.

32

Table S4: NMR (methanol-d_4) data for talaropeptide C (**4**)

	δ_H, mult, (*J* in Hz)	δ_C	COSY	HMBC	ROESY
***N*-Me-L-Ala^{1}**					
1		174.3			
2	5.44, m	51.4	3	1, 3, *N*-Me	*N*-Me-L-Val^{2}
3	1.27, d (*6.9*)	14.8	2	1, 2	
N-Me	3.14, s	31.7[i]		2, *N*-COCH$_3$	
N-COCH$_3$		173.6[j]			
N-COCH$_3$	1.99, s	22.3[k]		*N*-COCH$_3$	
***N*-Me-L-Val^{2}**					
1		170.1			
2	4.98, d (*10.9*)	61.5	3	1, 3, *N*-Me, *N*-Me-L-Ala^{1}-C-1	
3	2.24[a]	28.6	2, 4		
4	0.97, d (*6.6*)	19.9[l]	3	2, 3, 5	
5	0.82, d (*6.6*)	19.3		2, 3	
N-Me	3.01, s	31.1[m]		2, *N*-Me-L-Ala^{1}-C-1	N-Me-L-Ala^{1}-H-2
**L-Val^{3}**					
1		173.7[j]			
2	4.63, d (*7.6*)	56.2	3	1	*N*-Me-L-Ala^{1}
3	2.05[b]	31.8[i]	2, 4/5		
4	0.91[c]	19.9[l]	3	2	
5	0.93[c]	18.9[n]	3		
**L-Thr^{4}**					
1		174.0			
2	4.76[d]	56.6	3	1, 3, 4, L-Val^{3}-C-1	*N*-Me-L-Val^{5}
3	3.99, m	68.9	2, 4	1, 4	
4	1.17, d (*6.3*)	20.0[l]	3	2, 3	
***N*-Me-L-Val^{5}**					
1		172.0			
2	4.69, d (*11.1*)	63.6	3	1, 3, *N*-Me, *N*-Me-L-Thr^{4}-C-1	
3	2.24[a]	28. 0			
4	0.89[e]	19.9[l]		2, 3, 5	
5	0.82, d (*6.5*)	19.1[n]	3	2, 3, 5	
N-Me	3.20, s	31.8[i]		2, L-Thr^{4}-C-1	L-Thr^{4}-H-2
**L-Pro^{6}**					
1		174.8			
2	4.80, dd (*8.4*, *4.9*)	58.6	3a, 3b	3, 4	
3	a 2.21[f]	30.2[o]	2, 3b, 4a 4b	1, 2	
	b 1.66, m		2, 3b, 4b 4b	1, 2, 4	
4	a 2.05[b]	25.8[p]	3a, 3b, 5	2	
	b 1.92, m		3a, 3b, 5	2, 3	
5	3.71, m	48.9	4a, 4b	2, 3, 4	

***N*-Me-L-Val^{7}**					
1		171.7			
2	4.91, d (*10.6*)	60.2	3	3, 4, 5, *N*-Me, L-Pro^{6}-C-1	*N*-Me-L-Val^{8}
3	2.28, m	28.2[q]	4, 5		
4	0.90[e]	20.6			
5	0.86[g]	18.6			
N-Me	3.02, s	31.0[m]		2, L-Pro^{6}-C-1	L-Pro^{6}-H-2
***N*-Me-L-Val^{8}**					
1		171.1[r]			
2	5.03, d (*10.6*)	60.1	3	1, 3, 4, 5	*N*-Me-L-Phe^{9}
3	2.20[f]	28.3[q]	2, 4, 5		
4	0.84, d (*6.7*)	20.3	3		
5	0.67, d (*6.7*)	18.2	3	3, 4	
N-Me	2.27, s	30.3[o]		2, *N*-Me-L-Val^{7}-C-1	*N*-Me-L-Val^{7}-H-2
***N*-Me-L-Phe^{9}**					
1		172.9			
2	5.94, dd (*9.8,5.7*)	55.4	3	1, 3, *N*-Me, *N*-Me-L-Val^{8-}C-1	*N*-Me-L-Ile^{10}
3	3.05, m	35.9	2	4, 5/9	
4		138.2	3		
5/9	7.21, m	130.5	6/8	7, 5/9	
6/8	7.28, m	129.7	7, 5/9	4, 6/8	
7	7.24, m	128.2	6/8	5, 5/9	
N-Me	2.98, s	31.5[i]		2, *N*-Me-L-Val^{8-}C-1	*N*-Me-L-Val^{8}-H-2
***N*-Me-L-Ile^{10}**					
1		171.7[r]			
2	4.75[d]	62.5	3	3, 4, *N*-Me	
3	2.08, m	33.6	2, 4a, 4b		
4	a 1.32, m	25.9[p]	3, 4b		
	b 1.00, m		3, 4a		
5	0.87[g]	10.7		2	
6	0.96, d (*6.6)*	16.6	3		
N-Me	3.05, s	31.7[i]		2, *N*-Me-L-Phe^{9}-C-1	*N*-Me-L-Phe^{9}-H-2
**L-Leu^{11}**					
1		[nd]			
2	4.36, br s	[nd]	3		
3	1.62, m	42.4	2, 4		
4	1.61, m	26.5	3, 5/6		
5	0.93[c]	23.7			
6	0.92[c]	22.3[k]			

[a-h]Assignments with the same superscript are overlapping. [i-r] Assignments with the same superscript may be interchanged.
[nd] resonances not detected.

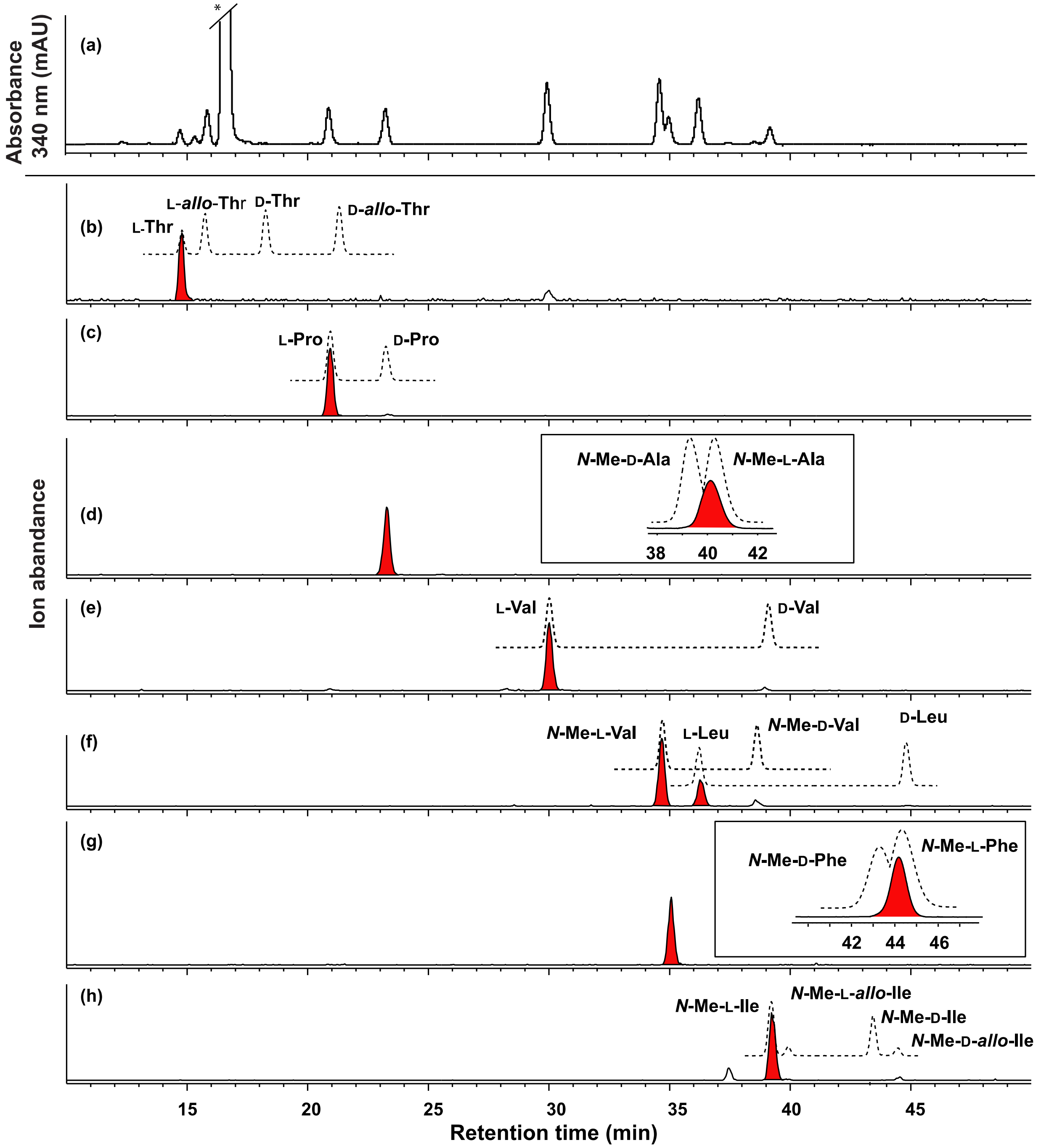

Figure S31: C3 Marfey's analysis for talaropeptide C (**4**). Trace (a) is the C3 HPLC-DAD (340 nm) chromatogram that exhibits L-FDAA amino acid derivatives. Traces (b-h) are C3 HPLC-MS-SIE chromatograms for L-FDAA derivatives of amino acid standards (broken lines) and the acid hydrolysate of **4** (shaded peaks). The inset in (d) shows the C18 HPLC-DAD (340 nm) chromatogram. Traces confirm that **4** incorporates (b) L-Thr (SIE *m/z* 372), (c) L-Pro (SIE *m/z* 368), (d) *N*-Me-L-Ala (SIE *m/z* 356) (e) L-Val (SIE *m/z* 370) (f) *N*-Me-L-Val and L-Leu (SIE *m/z* 384), (g) *N*-Me-L-Phe (SIE *m/z* 432) and (h) *N*-Me-L-Ile (SIE *m/z* 398). *Residual Marfey's reagent.

6 Talaropeptide D (5)

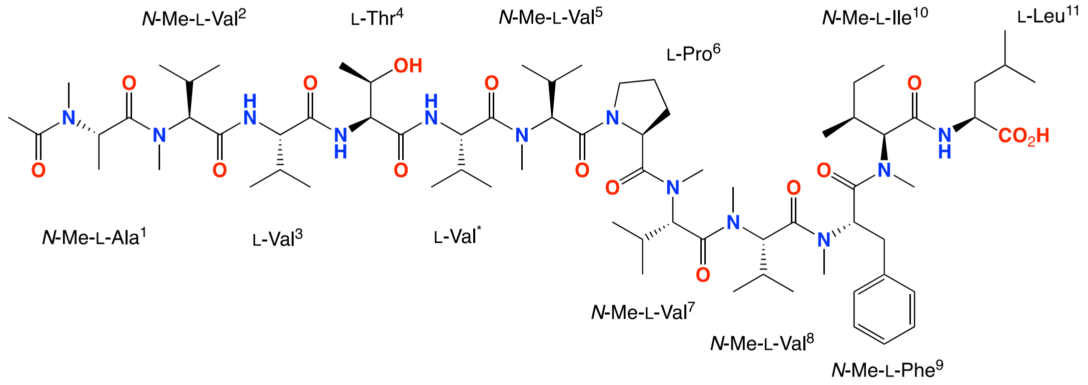

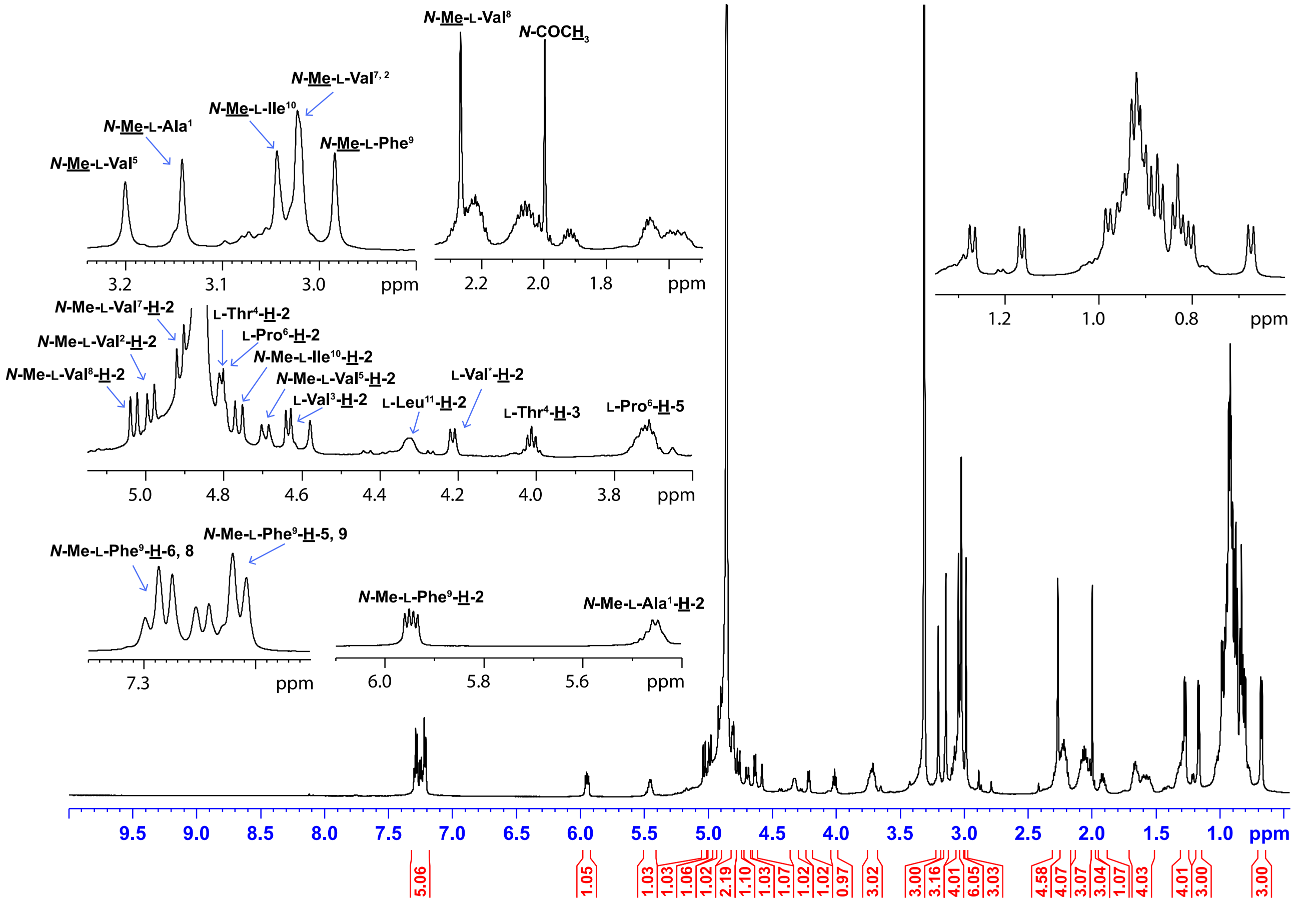

Figure S32: ^{1}H NMR (methanol-d_4) spectrum for talaropeptide D (**5**).

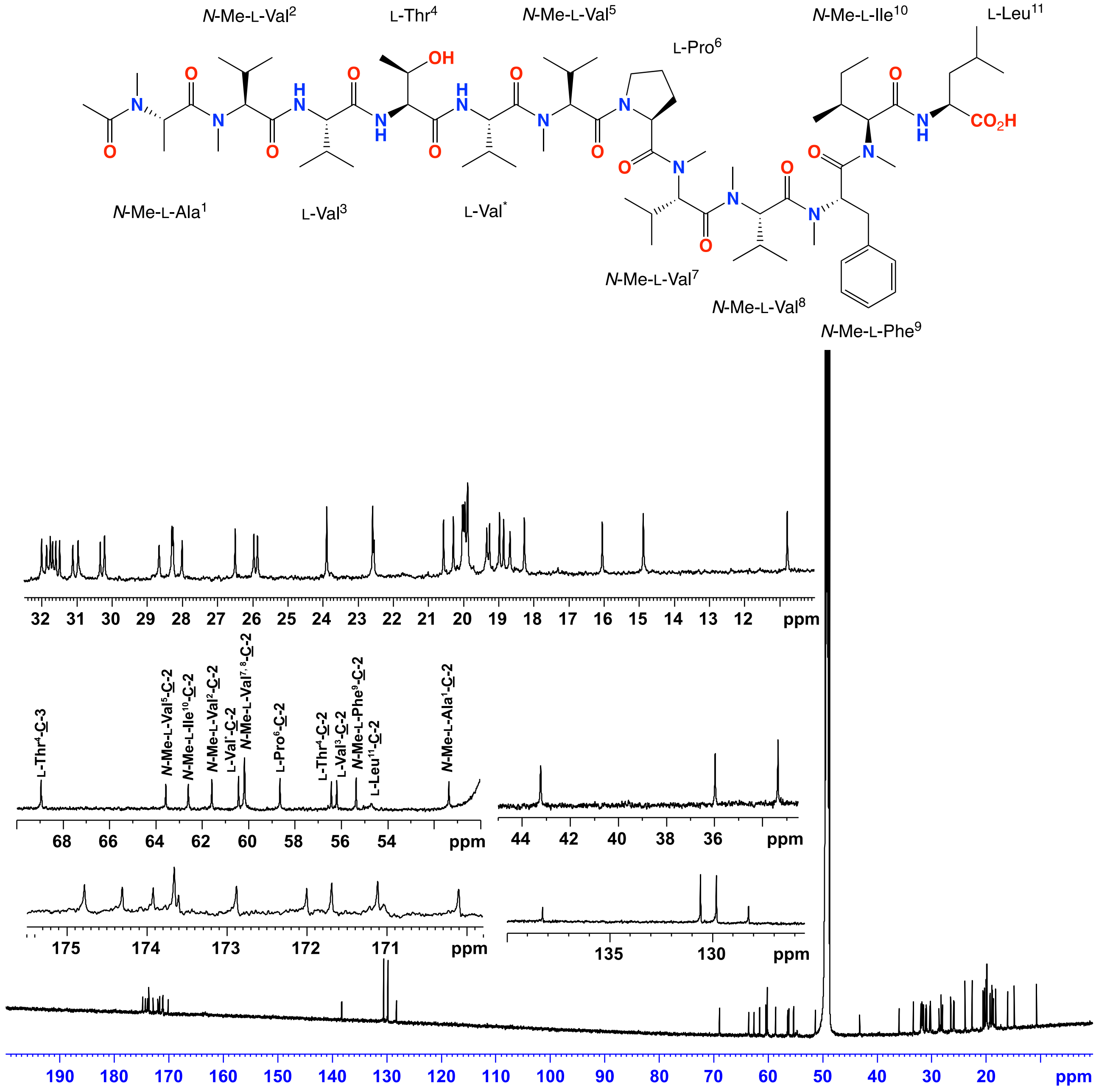

Figure S33: ^{13}C NMR (methanol-d_4) spectrum for talaropeptide D (**5**).

Supplementary Material

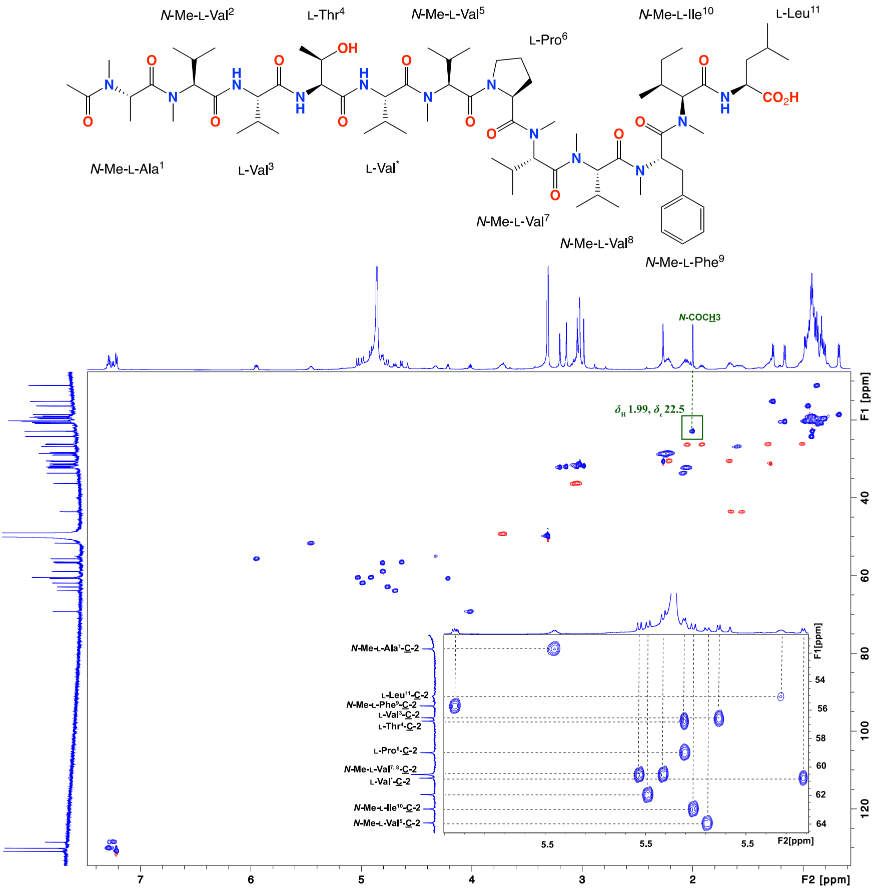

Figure S34: ^{1}H-^{13}C HSQC (methanol-d_4) spectrum for talaropeptide D (**5**). Expansion shows resonances for 12 amino acid α-protons. *N*-COC<u>H</u>$_3$ of *N*-Me- L-Ala[1] is highlighted in green.

38

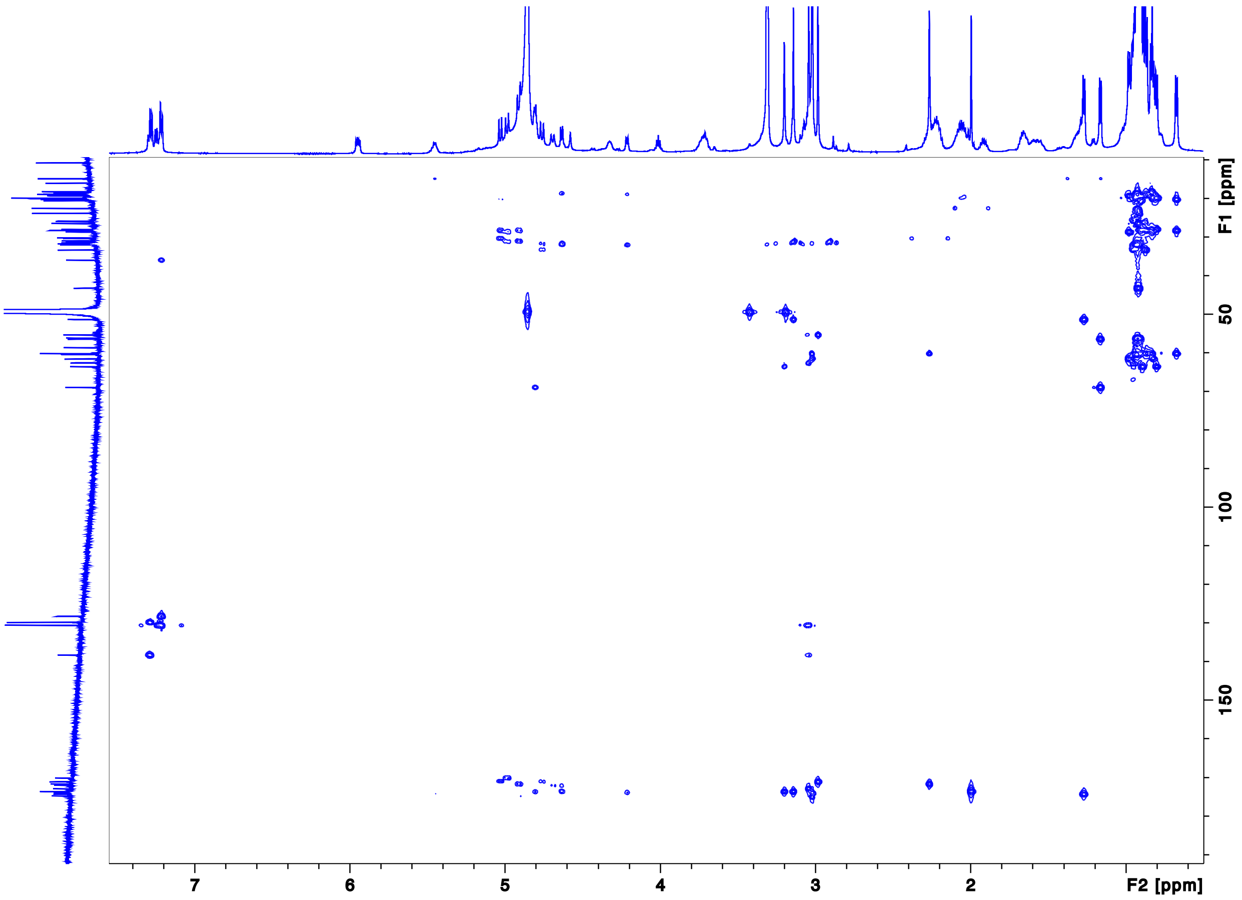

Figure S35: HMBC (methanol-*d*4) spectrum for talaropeptide D (**5**).

Supplementary Material

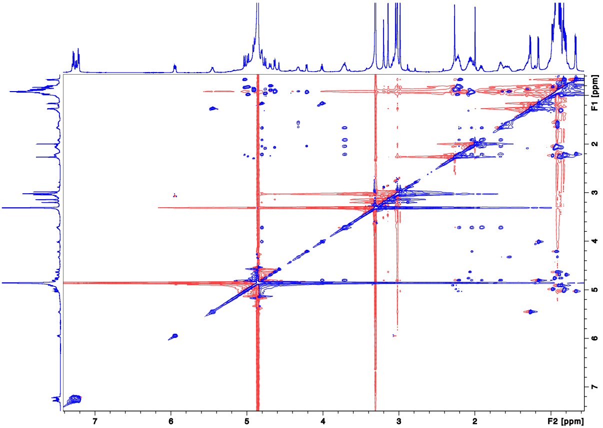

Figure S 36: TOCSY (methanol-*d*4) spectrum for talaropeptide D (**5**).

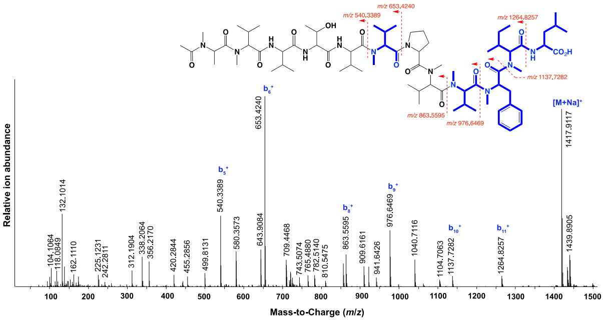

Figure S37: UHPLC-QTOF (MS/MS) spectrum of talaropeptide D (**5**).

40

Table S5: NMR data (methanol-d_4) for talaropeptide D (**5**)

	δ_H, mult, (*J* in Hz)	δ_C	COSY	HMBC	ROESY
***N*-Me-L-Ala[1]**					
1		174.3			
2	5.45, m	51.3	3	1, 3, *N*-Me	*N*-Me-L-Val[2]
3	1.27, d (*6.9*)	14.8	2	1, 2	
N-Me	3.14, s	31.8[g]		2, *N*-COCH$_3$	
N-COCH$_3$		173.6[h]			
N-COCH$_3$	1.99, s	22.5[i]		*N*-COCH$_3$	L-Val[2]-H-2
***N*-Me-L-Val[2]**					
1		170.1			
2	4.98, d (*10.9*)	61.5	3	1, 3, *N*-Me, *N*-Me-L-Ala[1]-C-1	
3	2.24, m	28.6	2, 4		
4	0.98, d (*6.5*)	19.9[j]	3	2, 3, 5	
5	0.82, d (*6.5*)	19.3[k]		2, 4	
N-Me	3.01, s	31.1[l]		2, *N*-Me-L-Ala[1]-C-1	L-Ala[1]-H-2
**L-Val[3]**					
1		173.7[h]			
2	4.63, d (*7.6*)	56.2	3	1, 3, 4, 5	*N*-Me-L-Ala[1]
3	2.05[a]	31.8[g]	2, 4		
4	0.92[b]	19.8[j]	3	2	
5	0.93[b]	18.9[m]			
**L-Thr[4]**					
1		173.7[h]			
2	4.80[c]	56.4	3	1, 3, 4	
3	4.01, m	68.8	2, 4		
4	1.16, d (*6.3*)	20.0[j]	3	2, 3	
**L-Val***					
1		173.9			
2	4.21, d (7.2)	60.4	3	1, 3, 4, 5	
3	2.03[a]	31.9[g]	2, 4/5		
4	0.93[b]	18.8[m]	3		5
5	0.92[b]	19.8[j]			
***N*-Me-L-Val[5]**					
1		172.0			
2	4.69, d (*11.0*)	63.6	3	1, 3, *N*-Me	
3	2.21[d]	28. 0	2, 5		
4	0.89, d (*6.5*)	19.8[j]			
5	0.80, d (*6.5*)	19.2[k]	3	2, 4	
N-Me	3.20, s	31.8[g]		2, L-Val*-C-1	
**L-Pro[6]**					
1		174.8			
2	4.80[c]	58.6	3a, 3b	4	
3	a 2.21[d]	30.2[n]	2, 3b, 4a, 4b	1, 2	
	b 1.66[e]		2, 3a, 4a, 4b	1, 2, 4	
4	a 2.05[a]	25.8[o]	3a, 3b, 4b, 5	2	

	b 1.92, m		3a, 3b, 4a, 5	2, 3	
5	3.71, m	48.9	4a, 4b	3, 4	
***N*-Me-L-Val^{7}**					
1		171.7			
2	4.91, d (*10.6*)	60.2	3	1, 3, 4, 5, *N*-Me, L-Pro^{6}-C-1	*N*-Me-L-Val^{8}
3	2.28, m	28.2^{p}	2, 4, 5		
4	0.90^{b}	18.6^{m}	3		
5	0.86^{f}	20.6	3		
N-Me	3.02, s	31.0^{l}		2, L-Pro^{6}-C-1	
***N*-Me-L-Val^{8}**					
1		171.0^{q}			
2	5.03, d (*10.6*)	60.2	3, 5	1, 2, 3	*N*-Me-L-Phe^{9}
3	2.20^{d}	28.3^{p}	2, 4, 5		
4	0.83, d (*6.7*)	20.3	3	5	
5	0.67, d (*6.7*)	18.2	2, 3	2, 3, 4	
N-Me	2.27, s	30.3^{n}		2, *N*-Me-L-Val^{7}-C-1	*N*-Me-L-Phe^{9}-H-2
***N*-Me-L-Phe^{9}**					
1		172.9			
2	5.94, dd (*9.8,5.7*)	55.4	3	1, 3, *N*-Me	*N*-Me-L-Phe^{9}
3	3.05, m	35.9	2	4, 5/9	
4		138.3	3		
5/9	7.21, m	130.5	6/8	7, 5/9	
6/8	7.28, m	129.7	7, 5/9	4, 6/8	
7	7.24, m	128.2	6/8	5/9	
N-Me	2.98, s	31.6^{g}		2, *N*-Me-L-Val^{8}-C-1	*N*-Me-L-Val^{8}-H-2
***N*-Me-L-Ile^{10}**					
1		171.1^{q}			
2	4.76, d (*11.2*)	62.6	3	3, 5, *N*-Me	
3	2.08, m	33.3	2, 4a, 4b, 6		
4	a 1.32, m	25.9^{o}	3, 4b		
	b 1.00, m		3, 4a		
5	0.87^{f}	10.7		2	
6	0.95, d (*6.5*)	16.0			
N-Me	3.05, s	31.7^{g}		2, *N*-Me-L-Phe^{10}-C-1	*N*-Me-L-Phe^{9}-H-2
**L-Leu^{11}**					
1		nd			
2	4.32, br s	54.7	3a, 3b		
3	a 1.65^{e}	43.2	2, 3b		
	b 1.55, m		2, 3a, 4	5/6	
4	1.59, m	26.5	5/6		
5	0.92^{b}	23.8	4		
6	0.91^{b}	22.5^{i}	4		

$^{a-f}$ Assignments with the same superscript are overlapping. $^{g-q}$ Assignments with the same superscript may be interchanged. nd resonances not detected.

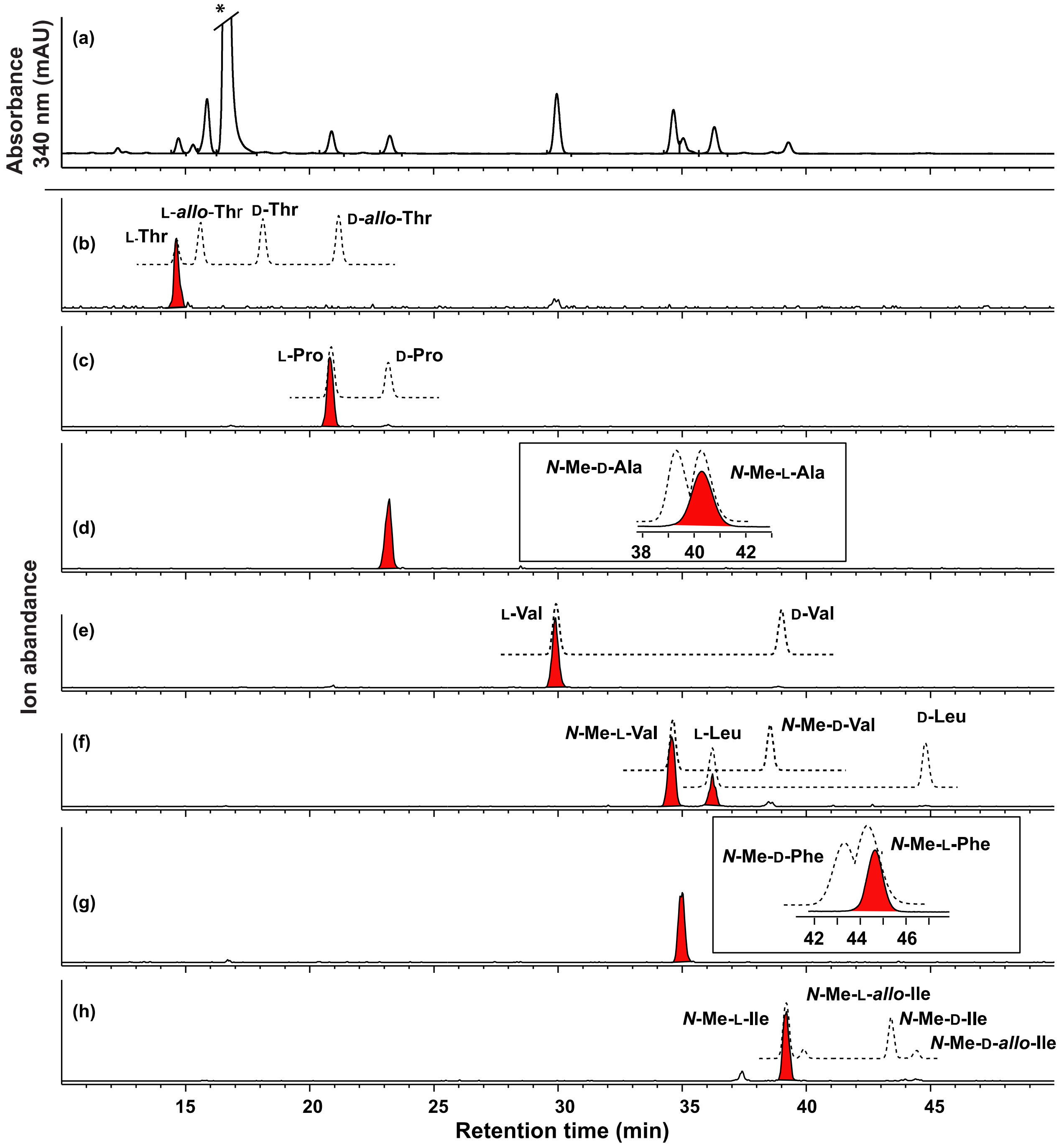

Figure S38: C3 Marfey's analysis for talaropeptide D (**5**). Trace (a) is the C3 HPLC-DAD (340 nm) chromatogram that exhibits L-FDAA amino acid derivatives. Traces (b-h) are C3 HPLC-MS-SIE chromatograms for L-FDAA derivatives of amino acid standards (broken lines) and the acid hydrolysate of **5** (shaded peaks). The inset in (d and g) shows the C18 HPLC-DAD (340 nm) chromatogram. Traces confirm that **5** incorporates (b) L-Thr (SIE *m/z* 372), (c) L-Pro (SIE *m/z* 368), (d) *N*-Me- L-Ala (SIE *m/z* 356) (e) L-Val (SIE *m/z* 370) (f) *N*-Me-L-Val and L-Leu (SIE *m/z* 384), (g) *N*-Me- L-Phe (SIE *m/z* 432) and (h) *N*-Me-L-Ile (SIE *m/z* 398). * residual Marfey's reagents

7 Plasma stability

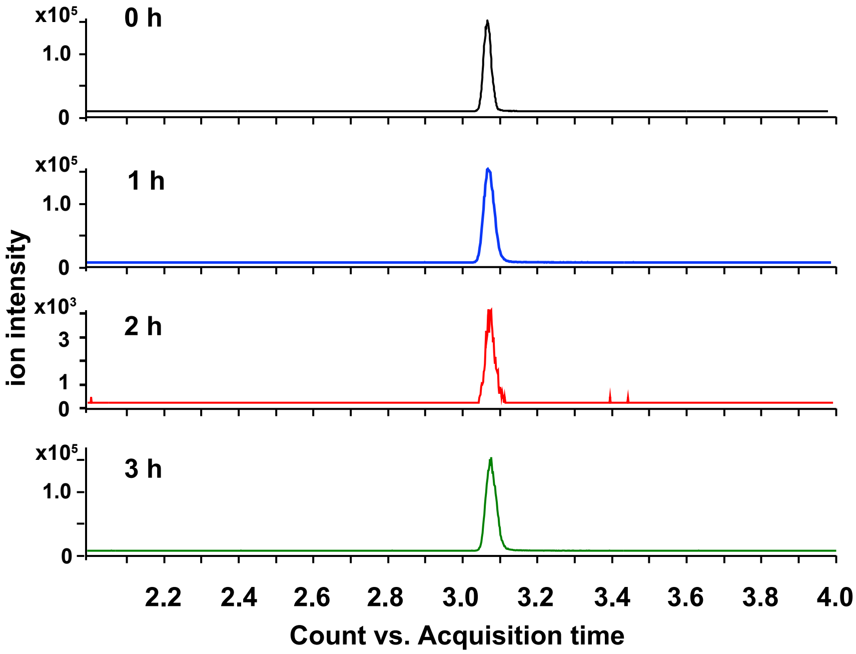

Figure S39: UHPLC-QTOF (MS) single ion extraction [1417.9, $(M+Na)^+$] chromatograms for talaropeptide D (**5**) incubated (37 °C) in rat plasma at different time points (0 – 3 h).

8 Biological assays

8.1 Cytotoxicity assay

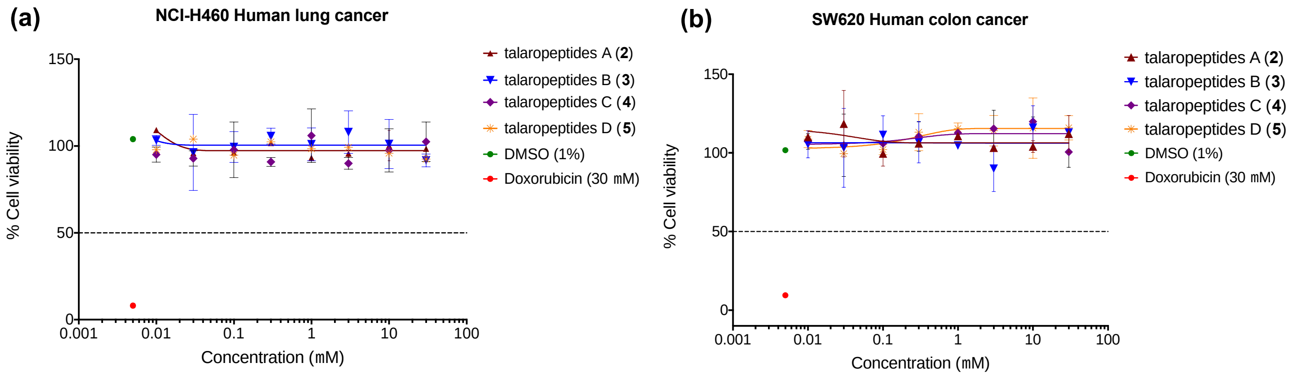

Figure S40: Screening of talaropeptides A-D (**2**-**5**) cytotoxicity against human cancer cells

Table S6: Cytotoxic activities (IC_{50}, μM) of 1 and 2

Cell line	**2**	**3**	**4**	**5**
SW620	>30	>30	>30	>30
NCI-H460	>30	>30	>30	>30

SW620: human colon carcinoma cell line

NCI-H460: human lung carcinoma cell line

8.2 Antibacterial assay

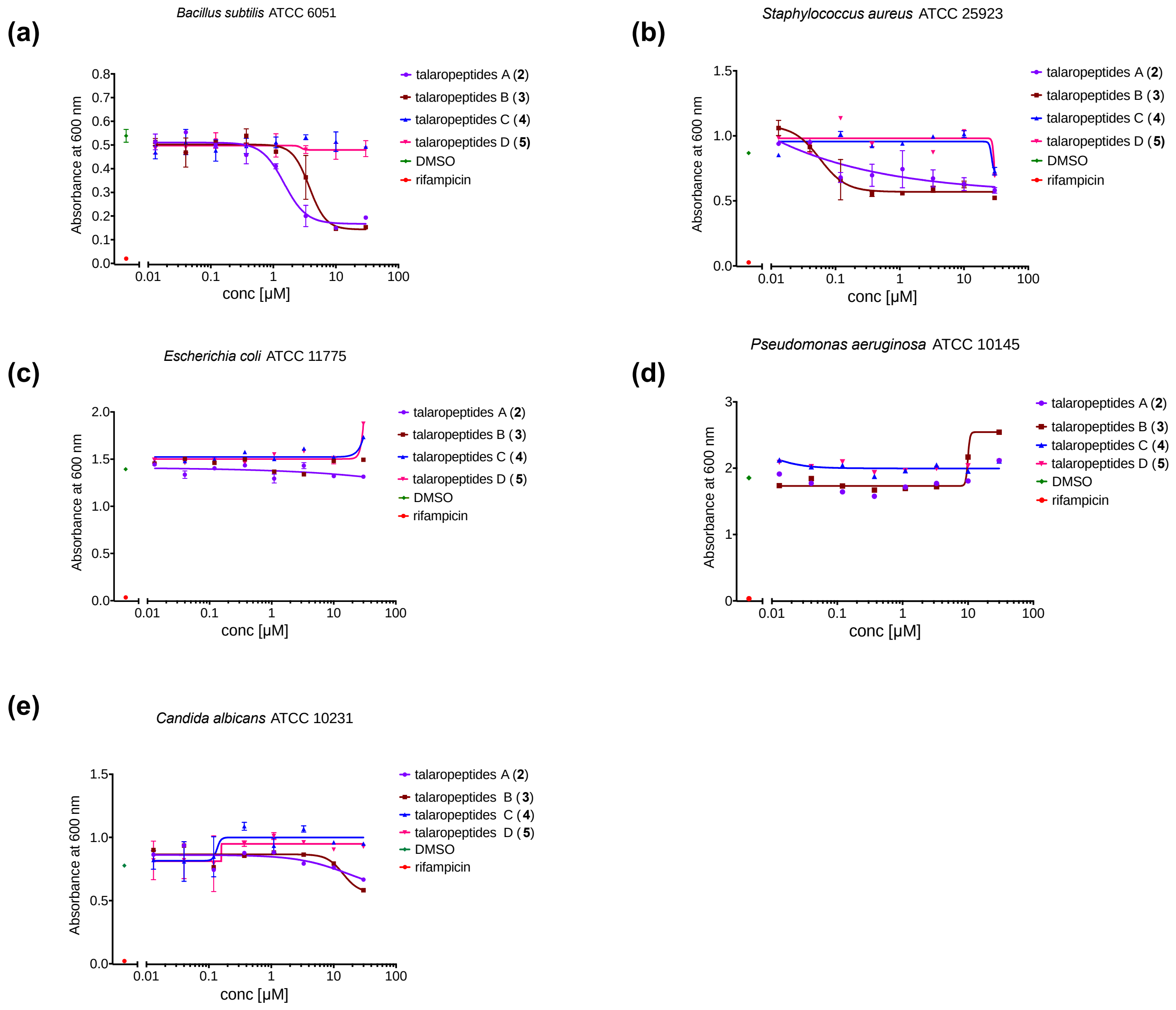

Figure S41: Growth inhibitory activity of talaropeptide A-D (**2-5**)

9 Genome mining

Table S7: Biosynthetic gene clusters identified from the genome of *Talromyces* sp. CMB-TU011

BGC	Class	Length (bases)
Cluster 1	Nrps	54341
Cluster 2	T1pks	25867
Cluster 3	T1pks	58657
Cluster 4	Terpene	14498
Cluster 5	T1pks-Nrps	17407
Cluster 6	Nrps	54562
Cluster 7	T1pks-Nrps	59596
Cluster 8	Indole	11095
Cluster 9	T1pks	9202
Cluster 10	Terpene	20118
Cluster 11	T1pks	4917
Cluster 12	Nrps	47837
Cluster 13	Other	25557
Cluster 14	Other	25196
Cluster 15	Other	30346
Cluster 16	Other	26175
Cluster 17	Other	41913
